# The Non‐Ancillary Nature of Trimethylsilylamide Substituents in Boranes and Borinium Cations

**DOI:** 10.1002/chem.202200698

**Published:** 2022-04-01

**Authors:** Christopher J. Major, Zheng‐Wang Qu, Stefan Grimme, Douglas W. Stephan

**Affiliations:** ^1^ Department of Chemistry University of Toronto 80 St. George St Toronto ON, M5S3H6 Canada; ^2^ Mulliken Center for Theoretical Chemistry University of Bonn Beringstr. 4 53115 Bonn Germany

**Keywords:** borenium cation, boranes, borinium cation, reaction mechanism, silylium cation

## Abstract

The known boranes (R(Me_3_Si)N)_2_BF (R=Me_3_Si **1** *, t*Bu **2**, C_6_F_5_ **3**, *o*‐tol **4**, Mes **5**, Dipp **6**) and borinium salts (R(Me_3_Si)N)_2_B][B(C_6_F_5_)_4_] (R=Me_3_Si **7**, *t*Bu **8**) are prepared and fully characterized. Compound **7** is shown to react with phosphines to generate [R_3_PSiMe_3_]^+^ and [R_3_PH]^+^ (R=Me, *t*Bu). Efforts to generate related borinium cations via fluoride abstraction from (R(Me_3_Si)N)_2_BF (R=C_6_F_5_ **3**, o‐tol **4**, Mes **5**) gave complex mixtures suggesting multiple reaction pathways. However for R=Dipp **6**, the species [(μ‐F)(SiMe_2_N(Dipp))_2_BMe][B(C_6_F_5_)_4_] was isolated as the major product, indicating methyl abstraction from silicon and F/Me exchange on boron. These observations together with state‐of‐the‐art DFT mechanistic studies reveal that the trimethylsilyl‐substituents do not behave as ancillary subsitutents but rather act as sources of proton, SiMe_3_ and methyl groups.

## Graphical abstract

**A series of borinium cations were generated** from boranes by fluoride extraction. The authors investigated the reaction mechanism using observational and density functional calculations and discuss how silyl‐amide substituents in boranes and borinium cations are shown not to be ancillary substituents, but rather act as sources of protons, SiMe_3_ and methyl groups.

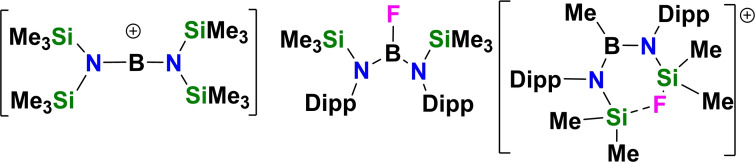

Since the 2005 review by Piers^[1]^ on boron cations, three‐coordinated borenium cations[^1^, ^2^] have been exploited as electrophiles in aromatic and aliphatic borylations, borylations of arylsilanes, and hydroboration of alkenes,^[3]^ chiral analogs have also been employed in enantioselective catalysis.^[4]^ In addition, NHC‐ and triazolylium‐borane derived borenium cations have been exploited in FLP‐hydrogenation^[5]^ as well as hydroborations^[6]^ of imines and enamines. In contrast, two‐coordinated borinium cations have garnered little attention. The classic 1982 work of Parry and coworkers^[7]^ and Nöth et al.^[8]^ reported the first examples of related diamido, alkyl/amido and aryl/amido‐substituted borinium cations (Figure 1), along with the first crystallographic evidence of their formation. However, little was known about their reactivity apart from reactions with solvent,^[9]^ halide sources,[^9a^, ^9d^] and Lewis bases.[^9c^, ^10^] Subsequent studies described the generation of borinium cations in the gas phase and their reactions with alcohols and amides.^[11]^ In 2002, we reported^[12]^ an “extended” borinium cation [(*t*Bu_3_PN)_2_B]^+^ (Figure 1). In a breakthrough work, Shoji et al. reported the first diaryl‐borinium, [Mes_2_B]^+^ in 2014 (Figure 1).^[13]^ These authors explored the reactivity of this species with CO_2_, CS_2_ and alkyne.^[14]^ In the latter case, an isolable divinylborinium cation was obtained (Figure 1). Recently, Inoue described the first example of a silicon‐substituted borinium cation [(HCNMe)_2_C=NBSi(SiMe_3_)_3_]^+^ (Figure 1).^[15]^

**Figure 1:**
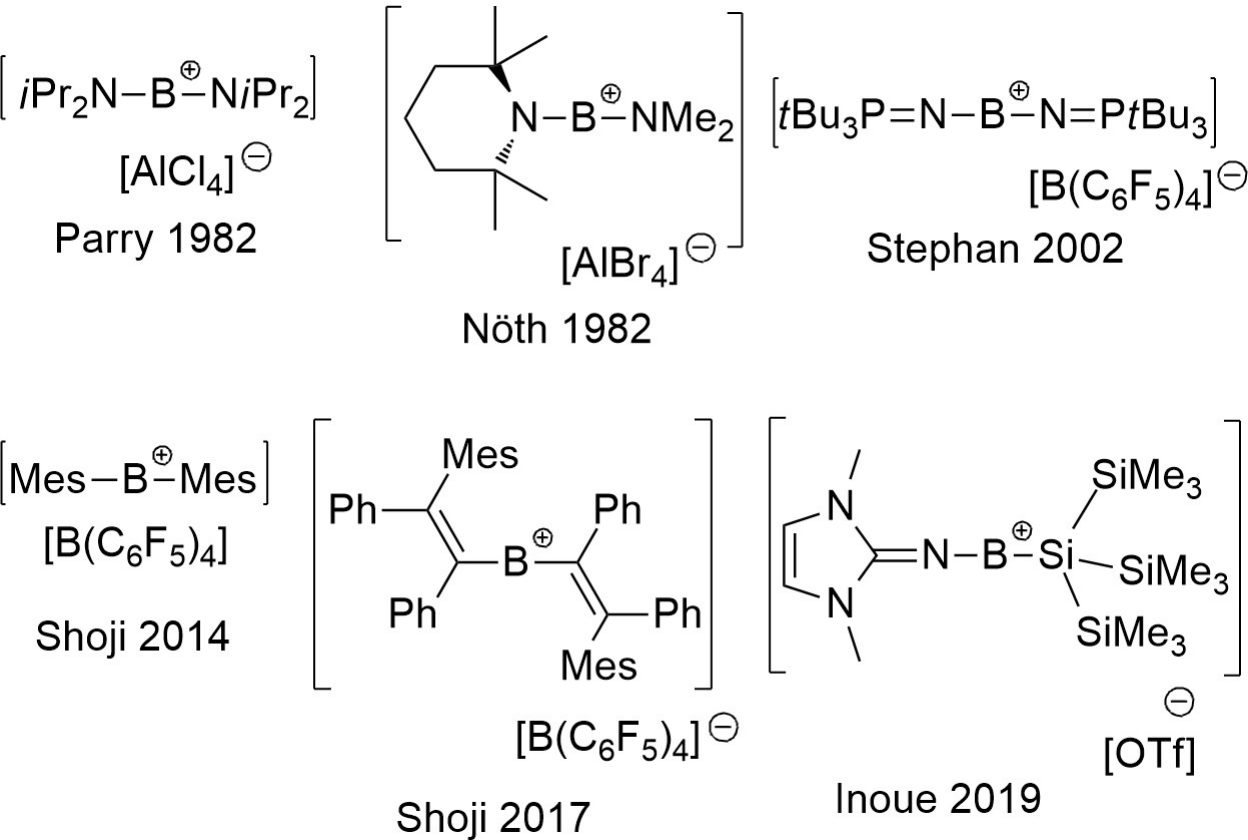
Selected examples of borinium cations.

In our own work,^[16]^ we have probed the reactivity of the di(amido)borinium cation, [(*i*Pr_2_N)_2_B][B(C_6_F_5_)_4_] demonstrating the unusual hydroboration of benzophenone, benzonitrile, diphenyldiazomethane, and phenylacetylene occurs with oxidation of one of the amide ligands to imine. We also probed the reactions of the Shoji cation, [Mes_2_B]^+^ with dihydrogen, silane, and borane uncovering routes to the unusual diboranium and triboron(8) cations, [B_2_H_2_Mes_3_]^+^ and [B_3_H_6_Mes_2_]^+^, respectively.^[17]^ Most recently, we reported the reactivity of aryl‐amido‐borenium cation [MesBN^*i*^Pr_2_]^+^ with isothiocyanate and carbodiimides which affords synthetic routes to nitrilium, mono‐amidinate borenium and bis‐amidinate boronium salts. In the present study, we probe the synthesis and reactivity of silyl‐amide substituted borinium cations. The results demonstrate that the silyl substituents are not innocent, acting as sources of silyl groups, proton and methyl fragments. The nature of this reactivity is probed experimentally and via detailed DFT computations.

A series of silyl‐aryl amides were reacted with (OEt_2_)BF_3_, affording the compounds (R(Me_3_Si)N)_2_BF (R=Me_3_Si **1** *, t*Bu **2**, C_6_F_5_ **3**, *o*‐tol **4**, Mes **5**, Dipp **6**) (Scheme 1) via a minor modification of the known literature protocol.^[18]^ These compounds exhibited ^11^B NMR signals at ca. 23–24 ppm, characteristic of three‐coordinated boron centers and showed ^19^F signals attributable to the BF fragment in the range from −85.5 to −107.7 ppm. Additionally, in the case of **3**, resonances at −147.2, −160.8 −165.2 ppm arose from the C_6_F_5_ rings. Compounds **3** and **6** were characterized by X‐ray crystallography (Figure 2) exhibiting the expected three‐coordinated geometry at boron. The N−B−N angles in **3** and **6** were found to be 128.9(7)° and 135.5(2)°, respectively. The wider angle in **6** reflects the steric demands of the aryl substituents. The corresponding B−N distances average 1.418(3) Å and 1.424(5) Å, while the B−F distances are 1.362(9) Å and 1.377(3) Å, respectively. The significantly shorter B−F bond in **3** is consistent with the presence of the electron withdrawing C_6_F_5_ rings. It is interesting to note the π‐stacking of the aryl rings as this dictates that the SiMe_3_ groups flank the B−F units.

**Scheme 1:**
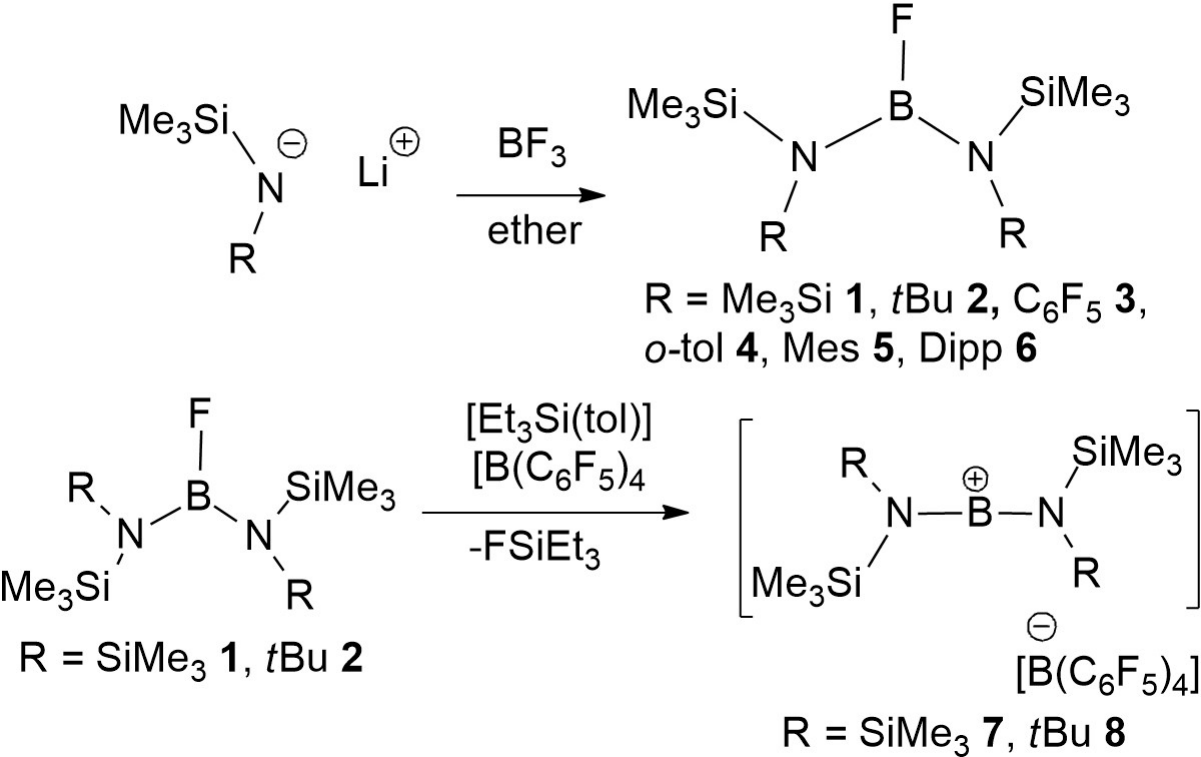
Synthesis of **1**–**8**.

**Figure 2:**
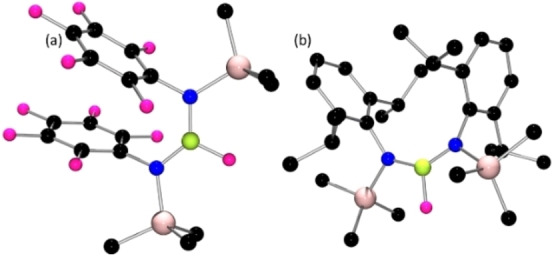
POV‐ray depiction of the molecular structures (a) **3**, (b) **6**; All hydrogen atoms have been omitted for clarity. C: black, N: blue, Si: pale pink, B: yellow‐green; F: pink.

The borinium cation salt [((Me_3_Si)_2_N)_2_B][B(C_6_F_5_)_4_] **7**, was prepared using the reaction of [Et_3_Si(tol)][B(C_6_F_5_)_4_] with **1** in *o*‐difluorobenzene ultimately affording a white product in 43 % yield. The ^1^H and ^13^C{^1^H} NMR data for the cation were consistent with previously reported data, while the ^11^B and ^19^F NMR spectra were consistent with the formulation of **7**. In addition, a crystallographic study confirmed the connectivity of this salt (Figure 3). The central boron of the cation is a linear and two‐coordinated with B−N bond distances of 1.330(4) and 1.328(4) Å. The compound **7** is directly analogous to the [BBr_4_]^−^ salt originally reported by Kölle and Nöth in 1986.^[19]^ The DFT‐computed Wiberg bond index of 1.52 for this cation of **7** is consistent with some degree of B−N π‐bonding.

**Figure 3:**
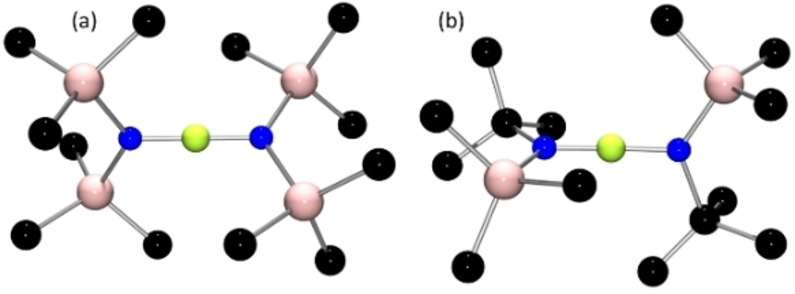
POV‐ray depiction of the molecular the cations of (a) **7**, (b) **8**. All hydrogen atoms have been omitted for clarity. C: black, N: blue, Si: pale pink, B: yellow‐green.

In a similar fashion, the analogous reaction of [*t*Bu(Me_3_Si)N]_2_BF afforded the species [(*t*Bu(Me_3_Si)N)_2_B][B(C_6_F_5_)_4_] **8**. This product exhibited the expected ^1^H and ^13^C NMR spectra as well as the ^11^B resonances at 34.6 and −16.6 ppm, corresponding to the anion and cation, respectively. Crystallographic data confirmed the connectivity (Figure 3). One of the amido‐groups is disordered via interchange of the *t*Bu and Me_3_Si groups. Nonetheless, the other amide group is not disordered, revealing N−C and N−Si distances of 1.538(5) Å and 1.820(3) Å with two B−N distances of 1.324(6) Å and 1.346(6) Å, respectively.

To gain mechanistic insights, extensive dispersion‐corrected DFT calculations were performed at the PW6B95‐D3+COSMO‐RS//TPSS−D3/def2‐TZVP+COSMO level in CHCl_3_ solution and the final free energies were reported in kcal/mol, at 298 K and 1 mol/L concentration^[20]^ (Figure 4). The separated ions of [Et_3_Si(tol)]^+^ and [B(C_6_F_5_)_4_]^−^ in solution provide a low barrier to fluoride abstraction from **1** of 11.9 kcal/mol (via transition state **TS1^+^**) providing the borinium cation of **7**, toluene and Et_3_SiF in an overall exergonic process (−23.5 kcal/mol). Interestingly, methyl abstraction from **1** with [Et_3_Si(tol)]^+^ (via **TS2^+^**) was computed to be nearly neutral in free energy over a low barrier of 12.6 kcal/mol affording the boryliminium cation **A^+^**, from which an intramolecular fluoride shift (via **TS3^+^**) is almost barrierless and −23.0 kcal/mol exergonic to form another borinium cation **7F^+^**. While not directly observed experimentally, this alternative reaction pathway could account for the moderate yield of **7**.

**Figure 4:**
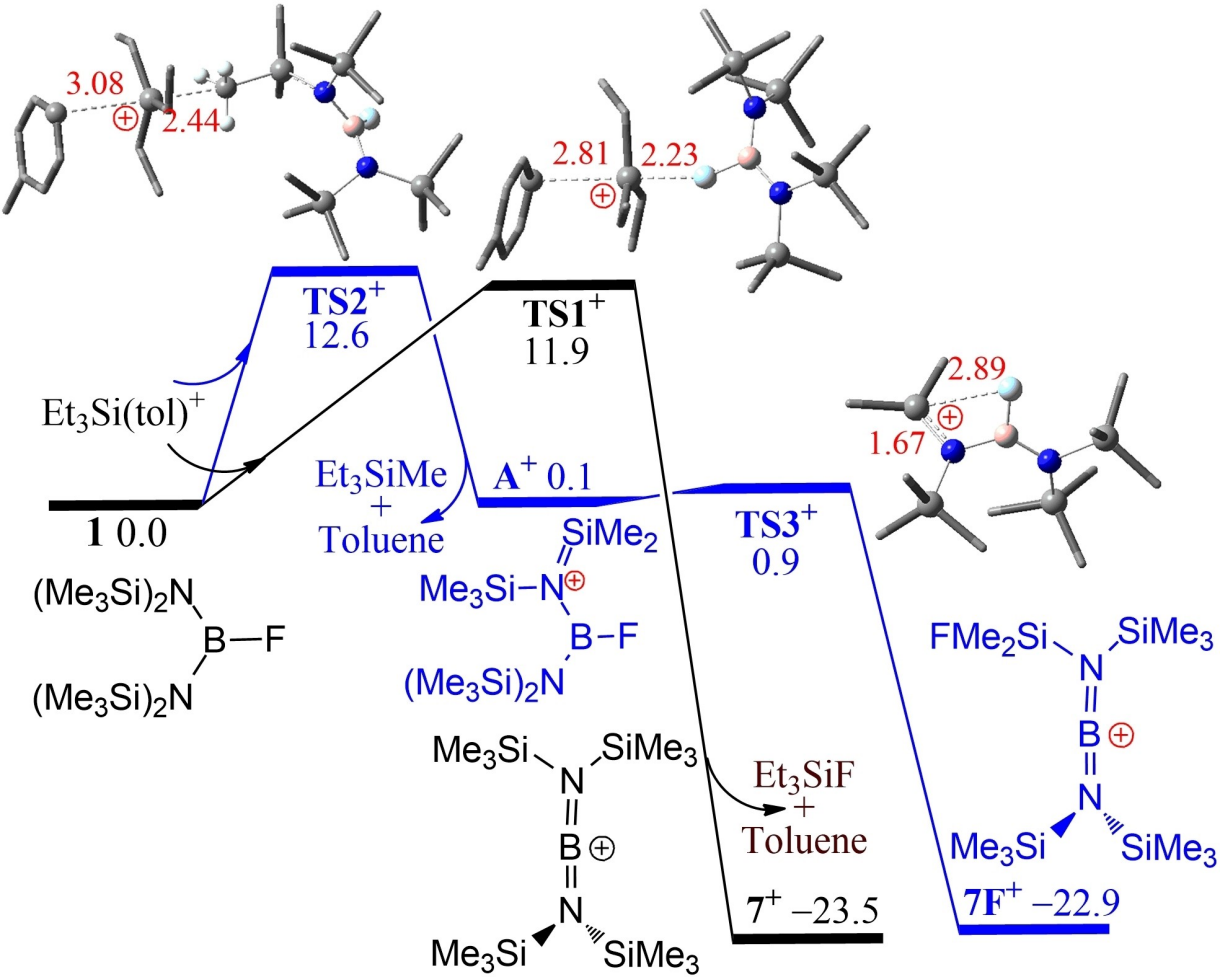
DFT‐computed free energy pathways (in kcal/mol, at 298 K and 1 M concentration) for the reaction of **1** with Et_3_Si(toluene)^+^. Crucial B, C, N, F and Si atoms are highlighted as pink, grey, blue, cyan and sky‐blue balls in ball‐and‐stick model, with selected bond lengths (in Å) shown in red. The transition states are labelled with the prefix TS.

DFT calculations were also used to probe the stabilizing effect of hyperconjugation between the silyl groups and the cationic boron center in **7** and **8**. The free energies of the isodesmic reactions with NH_3_ to give [(H_2_N)_2_B]^+^ and HN(SiMe_3_)(R) (R=SiMe_3_ and *t*Bu) were found to be 15.8 (4 SiMe_3_ groups) and 12.8 (2 SiMe_3_ groups) kcal/mol respectively. These results are consistent with about 3.9 and 2.4 kcal/mol smaller than a N−H bond for each NSiMe_3_ and N*t*Bu group, respectively.

Initial monitoring of the reaction of **7** with PMe_3_ showed a broad ^11^B peak at ca. 34.3 ppm while the ^31^P resonance was observed as a broad signal at −49.2 ppm. These data suggest the coordination of phosphine to boron, generating the proposed species [((Me_3_Si)N)_2_BPMe_3_][B(C_6_F_5_)_4_]. This view was supported by DFT computed ^11^B and ^31^P signals at 33.5 and −43.0 ppm, respectively. However, this species was not isolable, as it proved transient. Nonetheless, cooling to −30 °C afforded an insoluble product **9** which was isolated as colourless crystals albeit in low yield. A crystallographic study confirmed the formulation of **9** as [Me_3_PSiMe_3_][B(C_6_F_5_)_4_]. The metric parameters of the cation [Me_3_PSiMe_3_]^+^ are identical to those reported for its triflate salt,^[21]^ although it is noteworthy that the cation of **9** adopts an conformation in which the methyl groups on P and Si are staggered (Scheme 2). Also of interest, the ^31^P shift of **9** is ca. 26 ppm downfield of the triflate salt,^[21]^ consistent with the coordination of the triflate in solution. The formation of **9** results from the net loss of [SiMe_3_]^+^ from **1** generating the by‐product [(Me_3_Si)_2_NB(NSiMe_3_)]_2_ **10** a species previously prepared by dehydrohalogenation of (Me_3_Si)_2_NBCl(NHSiMe_3_) with BuLi.^[22]^ The observed ^11^B resonance at 27.1 ppm is consistent with this formulation, and corroborated by a recent report,^[23]^ although exhaustive efforts to isolate this by‐product were unsuccessful.

**Scheme 2:**
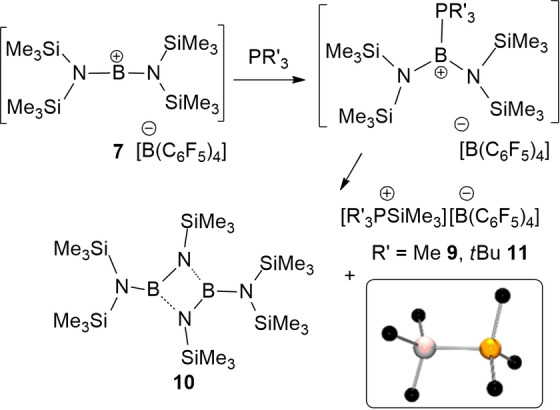
Synthesis of **9**–**11**. POV‐ray depiction of the cation of **9**, hydrogen atoms are omitted for clarity. C: black, N: blue, Si: pale pink, P: orange.

DFT calculations also revealed that the coordination of PMe_3_ to the borenium cation **7^+^** is 6.8 kcal/mol endergonic, to give the kinetically accessible but thermodynamically unstable borenium cation [((Me_3_Si)N)_2_BPMe_3_]^+^, **B^+^** (Figure 5), analogous to that proposed on the basis of experimental data. Alternatively, PMe_3_ may abstract a silylium cation [SiMe_3_]^+^ from **7^+^**, which is 4.6 kcal/mol endergonic over a low barrier of 8.9 kcal/mol (via **TS4^+^**) to form the observed cation **9^+^** and neutral (Me_3_Si)_2_NB=NSiMe_3_. Subsequent dimerization of the latter species is energetically favoured affording the product **10**, making the overall reaction −12.7 kcal/mol exergonic. In contrast, borinium‐assisted methyl deprotonation of the cation of **7** with PMe_3_ is 1.3 kcal/mol endergonic over a sizable barrier of 24.0 kcal/mol (via **TS5^+^**), which is thus both kinetically and thermodynamically disfavored. The use of bulky *t*Bu_3_P evidently increases the silylium abstraction barrier to 18.6 kcal/mol, while the methyl deprotonation channel becomes −5.0 kcal/mol exergonic though kinetically still less competitive. The corresponding reaction of **7** with *t*Bu_3_P gave a complex mixture of products. The observation of ^31^P resonances at 61.4 and 30.4 ppm (DFT: 53.2 and 21.0 ppm) were consistent with the formation of [*t*Bu_3_PH]^+^ and the previously reported cation [*t*Bu_3_PSiMe_3_]^+^,^[24]^ respectively. Crystals of [*t*Bu_3_PSiMe_3_][B(C_6_F_5_)_4_] **11** were isolated from the reaction albeit in low yield and the connectivity was confirmed by preliminary crystallographic data although disorder of the *tert*‐butyl groups precluded publication. The formation of these two phosphonium cations suggests that the borinium cation acts as a source of both proton and SiMe_3_ suggesting the formation of [(Me_3_Si)_2_NB(N(SiMe_3_)SiMe_2_CH_2_] and **10**, respectively. Again despite exhaustive efforts, these species could not be isolated, however these species were proposed based on computational data (Figure 5).

**Figure 5:**
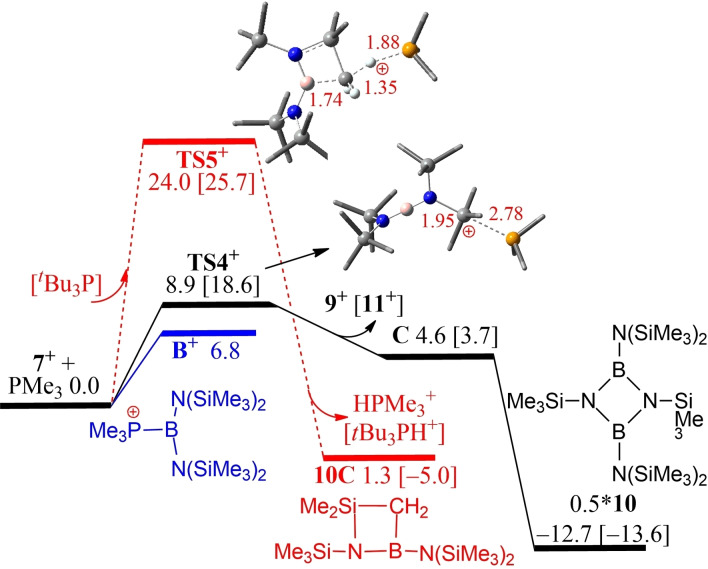
DFT‐computed free energy pathways for the reaction of cation **7** ^+^ with PMe_3_. An additional deprotonation channel (in red) is favoured by the bulky base P*t*Bu_3_ (as shown in bracket) The transition states are labelled with the prefix TS.

Efforts to extend reactivity studies to **3**–**6**, prompted the corresponding reactions with [Et_3_Si(tol)][B(C_6_F_5_)_4_]. In the case of **3** and **4**, NMR data suggested the formation of multiple products that were neither separable nor isolable. In the case of **5**, a small amount of crystalline material was obtained and while the quality of the crystals was poor, preliminary X‐ray data were consistent with its formulation as [(μ‐F)(SiMe_2_N(Mes))_2_BMe][B(C_6_F_5_)_4_]. However, the corresponding reaction of **6** afforded a cleaner reaction, with the major product **12** exhibiting a ^11^B resonances at 42.7 and −16.6 ppm. ^19^F revealed a resonance at −107.1 ppm, while the ^1^H spectrum shows resonances at −0.11, 0.80, 0.82, 1.20 and 1.31 ppm attributable to methyl groups in a 1: 2: 2: 4: 4 ratio. The precise structure of **12** was confirmed crystallographically to be [(μ‐F)(SiMe_2_N(Dipp))_2_BMe][B(C_6_F_5_)_4_] (Scheme 3). This species contains a three‐coordinated boron bound to two amides and a methyl substituent (Figure 6). A fluorine atom bridges two silicon atoms each of which have two methyl groups, affording an overall cationic charge. The B−N bond distances are 1.448(4) Å and 1.460(4) Å while the B−C bond is 1.566(4)Å, with the N−B−N 117.5(2)°. The Si−N distances were 1.717(2) Å and 1.706(2) Å with Si−F distances of 1.742(2) Å and 1.772(2) Å and a Si′−F−Si angle of 129.61(9)°.

**Scheme 3:**
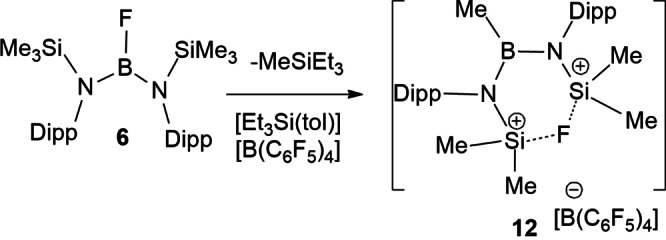
Synthesis of **12**.

**Figure 6:**
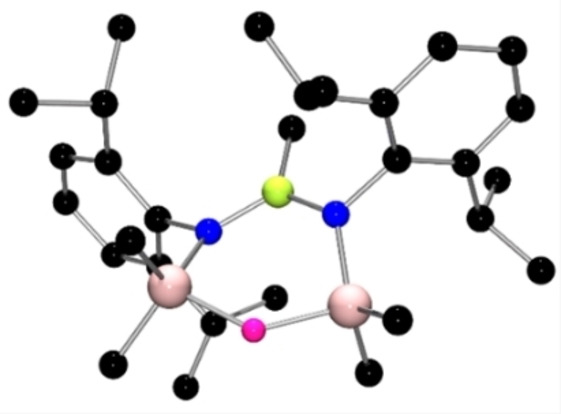
POV‐ray depiction of the molecular structure of the cation of **12**; All hydrogen atoms have been omitted for clarity. C: black, N: blue, Si: pale pink, B: yellow‐green; F: pink.

The formation of **12** is consistent with silylium abstraction of a methyl group from Si, and a fluoride for methyl from boron to silicon. In a very recent report by Chiu^[25]^ and coworkers, the transient generation of [MesBN(SiMe_3_)_2_]^+^ was observed to prompt methyl migration from Si to B. In that case, it gave the four‐membered‐ring species (Me_3_Si)N(SMe_2_)(μ‐Mes)BMe. To probe the present reaction further, DFT computations were performed. These revealed that selective fluoride‐assisted methyl‐abstraction from aryl‐substituted **6** by [Et_3_Si(tol)]^+^ is −8.9 kcal/mol exergonic over a low barrier of 8.3 kcal/mol (via **TS6^+^**) affording the intermediate cation **D^+^** with loss of Et_3_SiMe and toluene (Figure 7). Further a Si‐to‐B methyl‐shift (via **TS7^+^**) and even faster ring‐closing through B−N bond rotation of **E^+^** (via **TS8^+^**) is −12.0 kcal/mol exergonic over a higher barrier of 13.3 kcal/mol eventually affording the Si−F−Si bridged cation **12^+^**. In contrast, direct fluoride abstraction from **6** with [Et_3_Si(tol)]^+^ is −10.1 kcal/mol exergonic over a sizable barrier of 24.7 kcal/mol (via **TS6a^+^**, see Supporting Information) and is thus kinetically much less favorable, mainly due to enhanced steric hindrance and restrained B−N bond rotation of the bulky Dipp groups.

**Figure 7:**
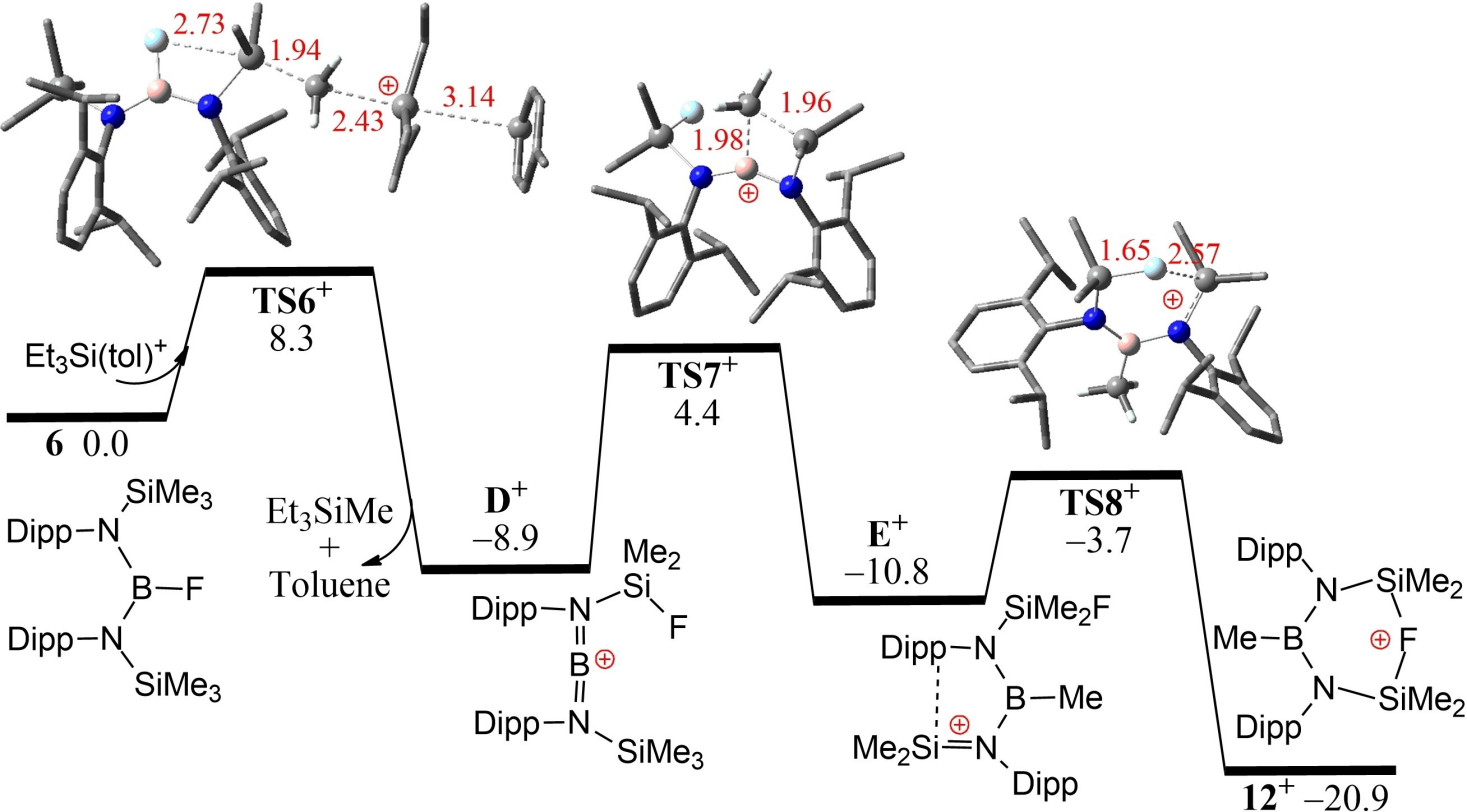
DFT‐computed free energy pathways for the reaction of **6** with [Et_3_Si(toluene)]^+^ The transition states are labelled with the prefix TS.

The above reactivity demonstrates that borinium cations are accessible via fluoride abstraction from **1** and **2**. These species react with phosphines readily undergo competitive desilylation or silylmethyl deprotonation reactions. In contrast, efforts to generate related borinium cations from **3**–**5** gave complex mixtures, suggesting multiple reaction pathways. The isolation of the heterocyclic cation **12** from the corresponding reaction of **6** demonstrates methyl abstraction from silicon as an alternative reaction pathway. Generally, these observations demonstrate that the silylamides in borane and borinium precursors are not ancillary ligands acting as sources of SiMe_3_^+^, H^+^ and Me groups. We are continuing to probe the reactivity of two‐coordinated boron cations, their Lewis acidity and potential use in catalysis.

## Crystallographic details

Deposition Numbers 2125379 (for **1**) 2125380 (for **12**) 2125381 (for **2**) 2125382 (for **6**) 2125383 (for **9**) contain the supplementary crystallographic data for this paper. These data are provided free of charge by the joint Cambridge Crystallographic Data Centre and Fachinformationszentrum Karlsruhe Access Structures service.

## Conflict of interest

The authors declare no conflict of interest.

## Supporting information

As a service to our authors and readers, this journal provides supporting information supplied by the authors. Such materials are peer reviewed and may be re‐organized for online delivery, but are not copy‐edited or typeset. Technical support issues arising from supporting information (other than missing files) should be addressed to the authors.

Supporting Information

## Data Availability

The data that support the findings of this study are available in the supplementary material of this article.
