# Supplementary material for: The Non‐Ancillary Nature of Trimethylsilylamide Substituents in Boranes and Borinium Cations
Source: Chemistry. 2022 Apr 1;28(27):e202200698. doi: 10.1002/chem.202200698 (PMC9324859; doi:10.1002/chem.202200698)
Supplement: Supplementary file 1 — Supporting Information [file CHEM-28-0-s001.pdf]

Chemistry–A European Journal

Supporting Information

The Non-Ancillary Nature of Trimethylsilylamide Substituents in Boranes and Borinium Cations

Christopher J. Major, Zheng-Wang Qu,* Stefan Grimme, and Douglas W. Stephan*

Materials and Methods **2**
General Considerations *2*

Syntheses **2**
Generation of FB(N(R)SiMe$_3$}$_2$)$_2$ R = C_6F_5 3, o-tol 4 and Mes 5 .... *2*
Synthesis of [((SiMe$_3$)$_2$N)$_2$B][B(C_6F_5)$_4$] 7 and [((tBu)SiMe$_3$)$_2$N)$_2$B][B(C_6F_5)$_4$] 8: *3*
Reaction of [({SiMe$_3$}$_2$N)$_2$B][B(C_6F_5)$_4$] with PMe$_3$ *4*
Reaction of [({SiMe$_3$}$_2$N)$_2$B][B(C_6F_5)$_4$] with PtBu$_3$ *4*
Independent synthesis of [Me$_3$PSiMe$_3$][B(C_6F_5)$_4$] 9 and [tBu$_3$PSiMe$_3$][B(C_6F_5)$_4$] 11: *4*
Synthesis of (μ-F)(SiMe$_2$N(Dipp))$_2$BMe][B(C_6F_5)$_4$] 12: *5*
Synthetic References *6*

NMR Spectra of Compounds. **7**
Figure 1. ^{1}H NMR spectrum of 3 in C_6D_6 at 298 K. *7*
Figure 2. ^{11}B{^{1}H} NMR spectrum of 3 in C_6D_6 at 298 K. *7*
Figure 3. ^{19}F{^{1}H} NMR spectrum of 3 in C_6D_6 at 298 K. *7*
Figure 4. ^{1}H NMR spectrum of 4 in C_6D_6 at 298 K. *8*
Figure 5. ^{11}B{^{1}H} NMR spectrum of 5 in C_6D_6 at 298 K. *8*
Figure 6. ^{19}F{^{1}H} NMR spectrum of 5 in C_6D_6 at 298 K. *8*
Figure 7. ^{1}H NMR spectrum of 5 in C_6D_6 at 298 K. *9*
Figure 8. ^{11}B{^{1}H} NMR spectrum of 5 in C_6D_6at 298 K. *9*
Figure 9. ^{19}F{^{1}H} NMR spectrum of 5 in C_6D_6 at 298 K. *9*
Figure 10. ^{1}H NMR spectrum of 7 in $CDCl_3$ at 298 K. *10*
Figure 11. ^{11}B{^{1}H} NMR spectrum of 7 in $CDCl_3$ at 298 K. *10*
Figure 12. ^{13}C{^{1}H} NMR spectrum of 7 in $CDCl_3$ at 298 K. *11*
Figure 13. ^{19}F{^{1}H} NMR spectrum of 7 in $CDCl_3$at 298 K. *11*
Figure 14. ^{1}H NMR spectrum of 8 in $CDCl_3$ at 298 K. *12*
Figure 15. ^{11}B{^{1}H} NMR spectrum of 8 in $CDCl_3$ at 298 K. *12*
Figure 16. ^{13}C{^{1}H} NMR spectrum of 8 in $CDCl_3$ at 298 K. *13*
Figure 17. ^{19}F{^{1}H} NMR spectrum of 8 in $CDCl_3$ at 298 K. *13*
Figure 18. ^{11}B{^{1}H} NMR of [({SiMe3}2N)2B][B(C6F5)4] with PMe3 in $CDCl_3$ at 298 K. *14*
Figure 19. ^{31}P{^{1}H} NMR of [({SiMe3}2N)2B][B(C6F5)4] with PMe3 in $CDCl_3$ at 298 K. *14*
Figure 20. ^{11}B{^{1}H} NMR of [({SiMe3}2N)2B][B(C6F5)4] with PtBu3 in CH_2Cl_2 at 298 K. *14*
Figure 21. ^{31}P{^{1}H} NMR of [({SiMe3}2N)2B][B(C6F5)4] with PtBu3 in CH_2Cl_2 at 298 K. *15*
Figure 22. ^{1}H NMR spectrum of 9 in C_6D_5Br at 298 K. *15*
Figure 23. ^{11}B{^{1}H} NMR spectrum of 9 in C_6D_5Br at 298 K. *15*
Figure 24. ^{19}F{^{1}H} NMR spectrum of 9 in C_6D_5Br at 298 K. *16*
Figure 25. ^{31}P{^{1}H} NMR spectrum of 9 in C_6D_5Br at 298 K. *16*
Figure 26. ^{1}H NMR spectrum of 11 in $CDCl_3$ at 298 K. *16*
Figure 27. ^{11}B{^{1}H} NMR spectrum of 11 in $CDCl_3$ at 298 K. *17*
Figure 28. ^{13}C{^{1}H} NMR spectrum of 11 in $CDCl_3$ at 298 K. Exponential apodization of 10.0000 Hz applied to resolve -C_6F_5 signals. *17*
Figure 29. ^{19}F{^{1}H} NMR spectrum of 11 in $CDCl_3$ at 298 K. *18*
Figure 30. ^{31}P{^{1}H} NMR spectrum of 11 in $CDCl_3$ at 298 K. *18*
Figure 31. ^{1}H NMR spectrum of 12 in $CDCl_3$ at 298 K. *18*
Figure 32. ^{11}B{^{1}H} NMR spectrum of 12 in $CDCl_3$ at 298 K. *19*

Figure 33. $^{13}C\{^1H\}$ NMR spectrum of 12 in $CDCl_3$ at 298 K. 19
Figure 34. $^{19}F\{^1H\}$ NMR spectrum of 12 in $CDCl_3$ at 298 K. 19

Computational data **20**
Computational Details: 20
Computational References 20
Table S1. TPSS-D3/def2-TZVP + COSMO computed imaginary frequency (ImF), zero-point energies (ZPE), gas-phase enthalpic (Hc) and Gibbs free-energy (Gc) corrections; the COSMO-RS computed solvation enthalpic (Hsol) and Gibbs free-energy (Gsol) corrections in THF solution; TPSS-D3/def2-QZVP and PW6B95-D3/def2-QZVP single-point energies (TPSS-D3 and PW6B95-D3); the total PW6B95-D3 free energies G_P; the relative electronic energies (ΔE_T and ΔE_P) and Gibbs free-energies (ΔG_T and ΔG_P) at the TPSS-D3 and PW6B95-D3 levels. 22
Table S2. TPSS-D3/def2-TZVP + COSMO optimized Cartesian coordinates (in Å) in $CHCl_3$ solution. Each structure is labeled by the specific name (See Table S1), followed by the number of atoms, the total energy (in hartrees), and the detailed atomic coordinates (in double-column text list). 24

Materials and Methods

General Considerations

All reactions and work-up procedures were performed under an inert atmosphere of dry, oxygen-free N using standard Schlenk techniques or a glovebox (Vac, equipped with a -35 °C freezer) unless otherwise specified. Pentane, dichloromethane, and toluene (Aldrich) were dried using a Grubbs-type Innovative Technologies solvent purification system. Deuterated solvents (C_6D_6, $CDCl_3$, C_6D_5Br) were purchased from Cambridge Isotope Laboratories, Inc. and stored over activated 4Å molecular sieves prior to use, unless otherwise specified. Boron trifluoride etherate was purchased from Alfa Aesar. All other reagents were purchased from Sigma-Aldrich. HN*o*-tolSiMe$_3$,[1] HNMesSiMe$_3$,[2] HN(C_6F_5)SiMe$_3$,[3] FB(N{SiMe$_3$}$_2$)$_2$ **1**,[4,5] FB(N(*t*Bu)SiMe$_3$)$_2$ **2**,[6] and FB(N(Dipp)SiMe$_3$)$_2$ **6**[7] were generated according to literature procedures. Crystals of **6** suitable for X-ray diffractometry were grown by dissolving in warm pentane and cooling to -30°.

NMR spectra were obtained on a Varian MercuryPlus 300 MHz, Bruker Avance III 400 MHz, Agilent DD2 500 MHz, or Agilent DD2 600 MHz spectrometer and spectra were referenced to residual solvent of $CDCl_3$ (^{1}H = 7.26; ^{13}C = 77.2), or externally (^{11}B, (Et_2O)BF_3; ^{19}F, $CFCl_3$; ^{31}P, 85% H_3PO_4). Chemical shifts (δ) are reported in ppm and coupling constants are listed in Hz. High-resolution mass spectra (HRMS) were obtained on an Agilent 6538 Q-TOF (ESI), JEOL AccuTOF Plus 4G (DART) and Bruker Autoflex Speed (MALDI). The extreme sensitivity of **4, 7,** and **12**, necessitating the use of MALDI precluded the acquisition of high-resolution MS data.

Syntheses

Generation of FB(N(R)SiMe$_3$}$_2$)$_2$ R = C_6F_5 3, *o*-tol 4 and Mes 5

These compounds were prepared in a similar fashion and thus only one preparation is detailed. In hexane, HN*o*-tolSiMe$_3$ was treated with one equivalent of *n*-BuLi. The lithium amide precipitated from solution as a white powder that was filtered and washed 3 times with hexane to furnish Li(N(*o*-tol)SiMe$_3$). LiN(*o*-tol)SiMe$_3$ (389.07 mg, 2.1 mmol) was dissolved in 5 mL ether and cooled to -30°. To this mixture was added a cooled

solution of BF_3OEt_2 (0.12 mL, 1.00 mmol) in 0.5 mL ether, at -30°, and stirred overnight. The solution was filtered and volatiles removed to afford an yellow oil, **4**.

3: A purple solid. Product was recrystalized from pentane, which was warmed before cooling to -30°. Due to high solubility of product, a 65% isolated yield. This compound proved to be highly sensitive as NMR data showed traces of $HN(C_6F_5)SiMe_3$. ^1H NMR (400 MHz, C_6D_6) δ 0.02 (s, 18H). ^{11}B NMR (128 MHz, C_6D_6) δ 23.17 (s, br). ^{19}F NMR (377 MHz, Benzene-*d*$_6$) δ -104.80 (s, 1F, FB), -146.42 – -146.64 (m, 4F, *o*-C_6F_5), -158.87 (t, $^3J_{F\text{-}F}$ = 21.7 Hz, *p*-C_6F_5), -163.53 – -163.76 (m, *m*-C_6F_5).

MS (TOF, DART+) m/z 539.10 (high res., calc. for $[C_{18}H_{19}BN_2F_{11}Si_2]^+$: 539.09987)

4: ^1H NMR (400 MHz, C_6D_6) δ 6.81 – 6.16 (m, 8H, ArH), 2.16 (s, 6H, *o*-PhC$\underline{H}_3$), 0.17 (s, 9H -$SiMe_3$), 0.17 (s, 9H -$SiMe_3$). ^{11}B NMR (128 MHz, C_6D_6) δ 23.73 (s, br). ^{19}F NMR (377 MHz, DMSO) δ -104.72 (s, B-F), -107.77 (B-F'). NB. Rotational isomers are observed; this is analogous to that reported for **6**.[7]

5: ^1H NMR (400 MHz, C_6D_6) δ 6.41 (s, 4H, H_a), 2.05 (s, 6H, H_b), 2.02 (s, 12H, H_c), 0.22 (s, 9H, H_d), 0.22 (s, 9H, H_d). ^{11}B NMR (128 MHz, C_6D_6) δ 23.36 (br). ^{19}F NMR (377 MHz, C_6D_6) δ -103.60.

MS (TOF, DARD+) m/z 527.38 (high res., calc. for $[C_{30}H_{53}BN_2F_1Si_2]^+$: 527.38189)

Synthesis of $[((SiMe_3)_2N)_2B][B(C_6F_5)_4]$ 7 and $[((t\text{Bu})SiMe_3)_2N)_2B][B(C_6F_5)_4]$ 8:
These compounds were prepared in a similar fashion and thus only one preparation is detailed. In toluene, equimolar $[CPh_3][B(C_6F_5)_4]$ and $HSiEt_3$ were used to generate 1 mmol $[Et_3Si][B(C_6F_5)_4]$. This was washed with 3 x 3 mL aliquots of pentane and dissolved in 2 mL of 1,2-difluorobenzene (ODFB), resulting in an orange solution. To a solution of (1.05 mmol) $((Me_3Si)_2N)_2BF$ in 3 mL ODFB was added an the $[Et_3Si][B(C_6F_5)_4]$ solution. After stirring 1 hour, volatiles were removed, and 312.4 mg of crude product was recrystalized from $CHCl_3$ at -30°. The supernatant was concentrated, and some pentane added, furnishing 123.9 mg of product at -30°. Crystals were washed with 3 x 2 mL aliquots of cold 1:1 $CHCl_3$ and pentane. ^1H and ^{11}B NMR data were consistent with previously reported values for the cation.[8]

7: 1H NMR (400 MHz, CDCl3) δ 0.46 (s, 36H -SiMe3). 11B{1H} NMR (128 MHz, CDCl3) δ 31.3 (s, br, [B]+), -16.6 (s, [B(C6F5)4]-). 13C{1H} NMR (101 MHz, CDCl3) δ 148.5 (d, br, 1J C-F = 244.50 Hz, m-C6F5), 138.32 (d, br, 1J C-F = 246.42 Hz, p-C6F5), 136.40 (d, br, 1J C-F = 250.95 Hz, o-C6F5), 124.15 (s, br, ipso-C6F5), 1.99 (s, -SiMe3). 19F NMR (377 MHz, CDCl3) δ -136.47 (s, o-C6F5), -166.92 (s, p-C6F5), -170.71 (s, m-C6F5).

8: ^1H NMR (400 MHz, $CDCl_3$) δ 1.52 (s, 18H, -CCH_3), 0.51(s, 18H -$SiMe_3$). $^{11}B\{^1H\}$ NMR (128 MHz, $CDCl_3$) δ 35.0 (s, br, $[B]^+$), -16.7 (s, $[B(C_6F_5)_4]^-$). ^{13}C NMR (101 MHz, $CDCl_3$) δ 148.33 (d, br, $^1J_{C\text{-}F}$ = 242.8 Hz, *m*-C_6F_5), 138.32 (d, br, $^1J_{C\text{-}F}$ = 244.3 Hz, *p*-C_6F_5), 136.39 (d, br, $^1J_{C\text{-}F}$ = 243.2 Hz, *o*-C_6F_5), 124.09 (s, br, *ipso*-C_6F_5), 61.38 (s, -$\underline{C}Me_3$), 32.58 (s, -C$\underline{Me}_3$), 2.42 (s, -$SiMe_3$). ^{19}F NMR (377 MHz, $CDCl_3$) δ -132.49, -163.05 (t, *J* = 20.4 Hz), -166.78 (t, *J* = 19.2 Hz).

MS (TOF, ESI+) m/z 298.2549 (high res., calc. for $[C_{14}H_{36}BN_2Si_2]^+$: 298.2541)

Reaction of [({$SiMe_3$}$_2$N)$_2$B][B(C_6F_5)$_4$] with PMe_3

To a solution of **7** (0.04 mmol, 40.4 mg) in 0.5 mL DCM, was added 0.4 µL of a 1M ODFB solution of PMe_3 (0.04 mmol) and stirred. Analysis of crude solution showed ^{31}P NMR signals corresponding to [Me_3SiPMe_3]$^+$, [({$SiMe_3$}$_2$N)$_2$BPMe$_3$]$^+$ (calculated ^{31}P NMR = -43.0 ppm); the ^{11}B NMR spectrum was suggestive of a mixture of [({$SiMe_3$}$_2$N)$_2$BPMe$_3$]$^+$ (calculated ^{11}B NMR = 33.5 ppm), unreacted [({$SiMe_3$}$_2$N)$_2$B]$^+$, ($SiMe_3$)$_2$N[BNSiMe$_3$]$_2$ **10**,[9] and [B(C_6F_5)$_4$]$^-$. By repeating this reaction and cooling reaction mixture to -30° for several days, crystals of [PMe_3SiMe_3][B(C_6F_5)$_4$] **9** suitable for X-ray diffractometry were isolated.

Reaction of [({$SiMe_3$}$_2$N)$_2$B][B(C_6F_5)$_4$] with P*t*Bu$_3$

A solution of **7** (0.04 mmol, 40.4 mg) in 0.5 mL DCM was added to P*t*Bu$_3$ (0.04 mmol, 8.1 mg) and stirred overnight. Analysis of crude solution showed ^{31}P NMR signals corresponding to [HP*t*Bu$_3$]$^+$, [*t*Bu$_3$PSiMe$_3$]$^+$,[10] unreacted P*t*Bu$_3$ in addition minor unidentified products; the ^{11}B NMR spectrum was suggestive of a mixture of unreacted [({$SiMe_3$}$_2$N)$_2$B]$^+$, **10**,[9] and [B(C_6F_5)$_4$]$^-$. By repeating this reaction and cooling reaction mixture to -30° for several days, crystals were grown for X-ray diffractometry of [*t*Bu$_3$PSiMe$_3$][B(C_6F_5)$_4$] **11**. However, the data are too distorted to discuss bonding parameters.

Independent synthesis of [Me_3PSiMe_3][B(C_6F_5)$_4$] 9 and [*t*Bu$_3$PSiMe$_3$][B(C_6F_5)$_4$] 11:

These compounds were prepared in a similar fashion and thus only one preparation is detailed. A solution of PMe_3 in ODFB was added to DCM, followed by $SiMe_3$OTf and the vial was agitated to mix. After 30 minutes, the solution was added to a stirred suspension of Na[B(C_6F_5)$_4$] in DCM. The resultant white suspension was stirred for 30 minutes before filtration and removal of volatiles. The NMR spectra were consistent with the formation of the desired product contaminated with [$HPMe_3$]$^+$, assumed to be a result of adventitious moisture and the high sensitivity of the cation of 9.

9*:* ^{1}H NMR (400 MHz, C_6D_5Br) δ 0.84 (d, $^2J_{H-P}$ = 11.8 Hz, 9H, -P(CH_3)$_3$), -0.07 (d, $^3J_{H-P}$ = 9.3 Hz, 9H, -Si(CH_3)$_3$). ^{11}B NMR (128 MHz, C_6D_5Br) δ -16.64 (s, br, [B(C_6F_5)$_4$]). ^{19}F NMR (377 MHz, C_6D_5Br) δ -132.14 (s, br, *o*-C_6F_5) -161.74 (t, $^3J_{F-F}$ = 21.1 Hz, *p*-C_6F_5), -165.82 (t, $^3J_{F-F}$ = 18.2 Hz, *m*-C_6F_5). ^{31}P{^{1}H} NMR (162 MHz, C_6D_5Br) δ -26.90 (s, [Me_3PSiMe_3]). NB. The corresponding triflate salt was previously reported to form reversibly from PMe_3 and Me_3SiOTf.[11]

MS (TOF, ESI+) m/z 149.0913 (high res., calc. for [$C_6H_{18}PSi$]$^+$: 149.0910)

11: ^{1}H NMR (400 MHz, $CDCl_3$) δ 1.65 (d, $^3J_{H-P}$ = 14.2 Hz, 27H, PC(CH_3)$_3$), 0.81 (d, $^3J_{H-P}$ = 5.0 Hz, 9H, Si(CH_3)$_3$). ^{11}B NMR (128 MHz, $CDCl_3$) δ -16.65 [B(C_6F_5)$_4$]. ^{13}C NMR (101 MHz, $CDCl_3$) δ 148.32 (d, $^1J_{C-F}$ = 242.4 Hz, *m*-C_6F_5), 138.39 (d, $^1J_{C-F}$ = 228.6 Hz, *p*-C_6F_5), 136.47 (d, $^1J_{C-F}$ = 231.5 Hz, *o*-C_6F_5), 41.26 (d, $^1J_{C-P}$ = 15.8 Hz, P$\underline{C}$(CH_3)$_3$), 31.05 (s, br, PC($\underline{C}H_3$)$_3$), 4.30 (d, $^2J_{C-F}$ = 7.0 Hz, Si(CH_3)$_3$). ^{19}F NMR (377 MHz, $CDCl_3$) δ -132.44 (br, *o*-C_6F_5), -163.17 (t, $^3J_{F-F}$ = 20.9 Hz, *p*-C_6F_5), -166.83 (t, $^3J_{F-F}$ = 18.4 Hz, *m*-C_6F_5), *ipso*-C_6F_5 signal not observed due to limited solubility. ^{31}P{^{1}H} NMR (162 MHz, $CDCl_3$) δ 30.30 (s, [^{t}Bu$_3$PSiMe$_3$]$^+$). NB: The cation of this salt has been previous reported.[10]

Synthesis of [(μ-F)($SiMe_2$N(Dipp))$_2$BMe][B(C_6F_5)$_4$] 12:

In toluene, equimolar [CPh_3][B(C_6F_5)$_4$] and $HSiEt_3$ was used to generate 0.133 mmol [Et_3Si][B(C_6F_5)$_4$]. This was washed with 3 x 3 mL aliquots of pentane and dissolved in 2 mL of ODFB, resulting in an orange solution. This was cooled to -30°. To a pre-cooled solution of **6** (77.8 mg, 0.145 mmol) in 3 mL ODFB was added the [Et_3Si][B(C_6F_5)$_4$] solution. After stirring 1 hour, volatiles were removed, and the product was recrystalized from $CHCl_3$ at -30°, furnishing 65.3 mg (37%). Crystals were washed with 3 x 1 mL aliquots of cold $CHCl_3$.

^{1}H NMR (700 MHz, $CDCl_3$) δ 7.40 – 7.35 (m, 2H, H_f), 7.28 – 7.24 (m, 4H, H_e), 3.08 (hept, *J* = 6.7 Hz, 4H, H_b), 1.31 (d, *J* = 6.9 Hz, 13H, H_a), 1.20 (d, *J* = 6.8 Hz, 13H, $H_{a'}$), 0.82 (s, 6H, -H_i), 0.80 (s, 6H, $H_{h'}$), -0.11 (s, 3H H_g). ^{11}B NMR (128 MHz, $CDCl_3$) δ 42.30 (s, br, [(μ-F)($SiMe_2$N(Dipp))$_2$<u>B</u>Me]$^+$), -16.64 (s, br, [B(C_6F_5)$_4$]$^-$). ^{13}C NMR (126 MHz, $CDCl_3$) δ 145.45 (s, C_d), 133.77 (s, C_c), 129.15 (s, C_f), 125.70 (s, C_e), 28.93 (s, C_b), 24.55 (s, C_a), 24.44 (s, $C_{a'}$), -0.07 (s, C_h), -0.24 (s, $C_{i'}$); C_g and [B(C_6F_5)$_4$]$^-$ signals not observed due to solubility. ^{19}F NMR (377 MHz, $CDCl_3$) δ -106.90 – -107.21 (m, [(μ-F)($SiMe_2$N(Dipp))$_2$BMe]$^+$), -132.54 (s, br, *o*-C_6F_5), -162.87 (t, *J* = 20.6 Hz, *p*-C_6F_5), -166.69 (s, br, *m*-C_6F_5). *MS* (TOF, MALDI+) m/z 511.4 ([$C_{30}H_{52}BN_2F_1Si_2$]$^+$).

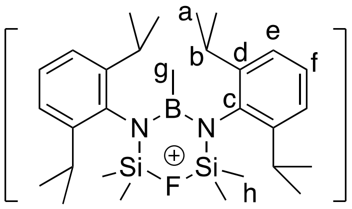

Synthetic References

[1] A. B. Smith, M. Visnick, J. N. Haseltine, P. A. Sprengeler, *Tetrahedron* **1986**, *42*, 2957–2969.

[2] R. Murugavel, V. Chandrasekhar, A. Voigt, H. W. Roesky, H.-G. Schmidt, M. Noltemeyer, *Organometallics* **1995**, *14*, 5298–5301.

[3] A. J. Oliver, W. A. G. Graham, *Journal of Organometallic Chemistry* **1969**, *19*, 17–27.

[4] P. Geymayer, E. G. Rochow, U. Wannagat, *Angew. Chem. Int. Ed. Engl.* **1964**, *3*, 633–633.

[5] P. Geymayer, E. G. Rochow, U. Wannagat, *Angew. Chem.* **1964**, *76*, 499–500.

[6] P. Paetzold, E. Schröder, G. Schmid, R. Boese, *Chem. Ber.* **1985**, *118*, 3205–3216.

[7] G. Elter, A. Meller, W. Luthin, *Z. Anorg. Allg. Chem.* **1988**, *560*, 18–26.

[8] P. Kölle, H. Nöth, *Chem. Ber.* **1986**, *119*, 3849–3855.

[9] A. Matler, M. Arrowsmith, F. Schorr, A. Hermann, A. Hofmann, C. Lenczyk, H. Braunschweig, *Eur. J. Inorg. Chem.* **2021**, *2021*, 4619–4631.

[10] M. A. Dureen, C. C. Brown, D. W. Stephan, *Organometallics* **2010**, *29*, 6594–6607.

[11] A. P. M. Robertson, S. S. Chitnis, S. China, H. J. Cortes S., B. O. Patrick, H. A. Jenkins, N. Burford, *Can. J. Chem.* **2016**, *94*, 424–429.

NMR Spectra of Compounds.

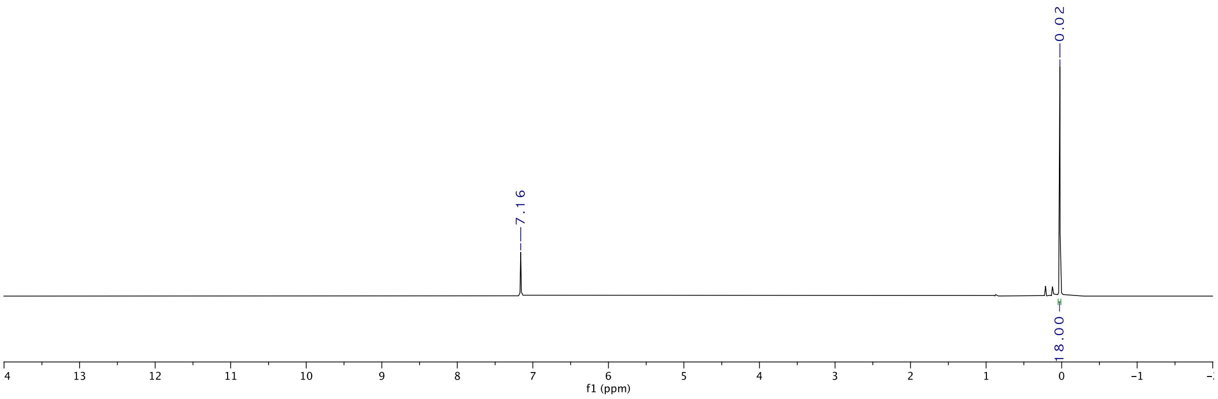

Figure 1. ^{1}H NMR spectrum of 3 in C_6D_6 at 298 K.

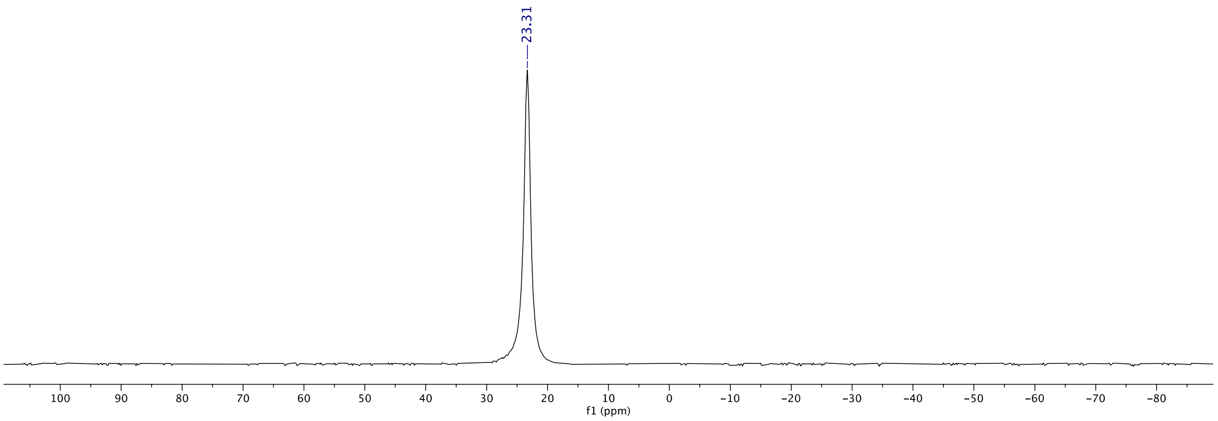

Figure 1. ^{11}B{^{1}H} NMR spectrum of 3 in C_6D_6 at 298 K.

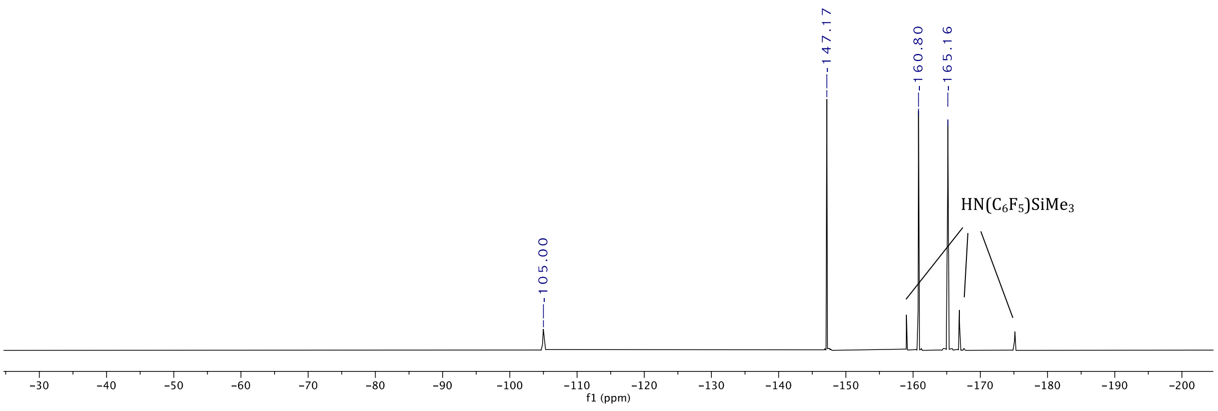

Figure 2. ^{19}F{^{1}H} NMR spectrum of 3 in C_6D_6 at 298 K.

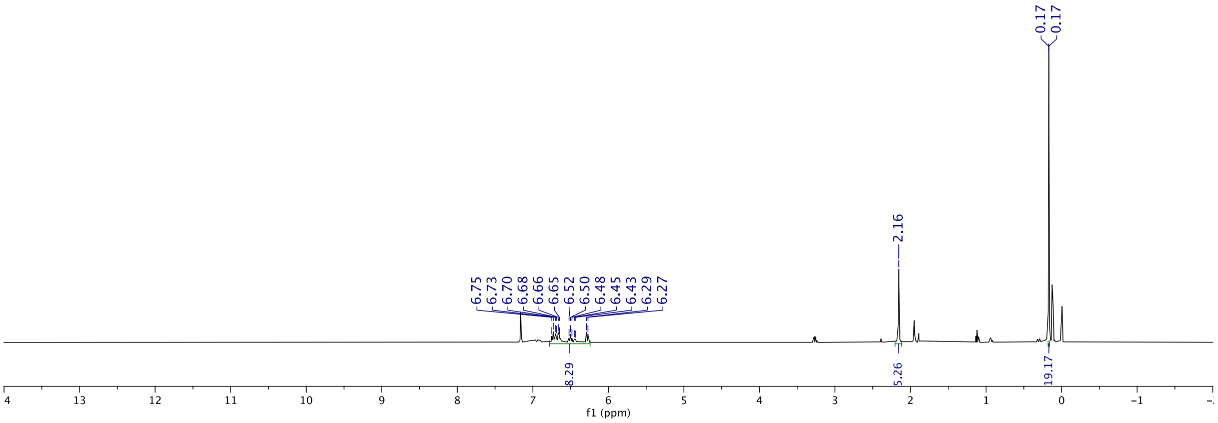

Figure 3. ^{1}H NMR spectrum of 4 in C_6D_6 at 298 K.

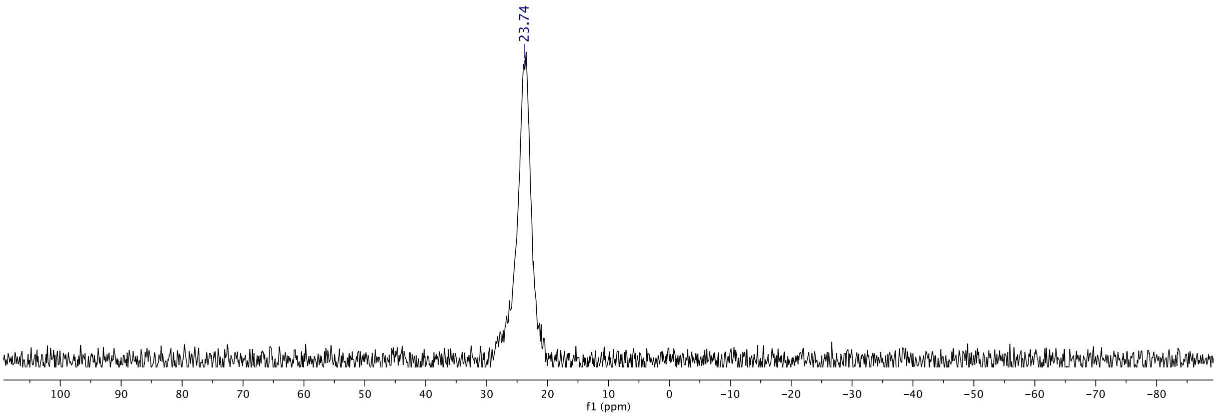

Figure 4. $^{11}B\{^{1}H\}$ NMR spectrum of 5 in C_6D_6 at 298 K.

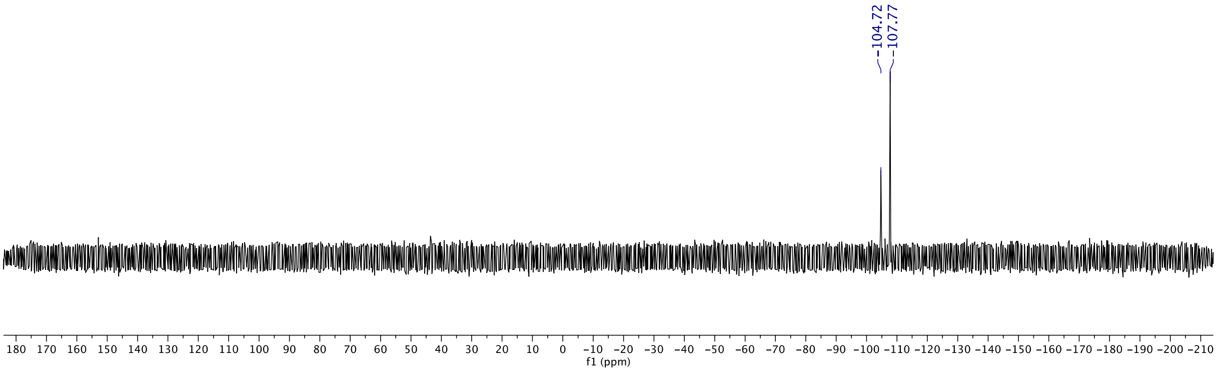

Figure 5. $^{19}F\{^{1}H\}$ NMR spectrum of 5 in C_6D_6 at 298 K.

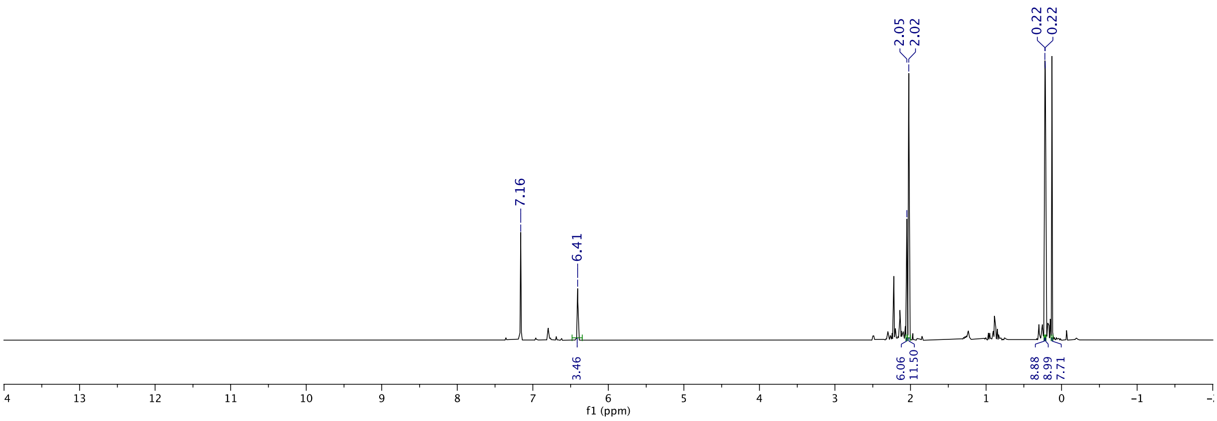

Figure 6. ^{1}H NMR spectrum of 5 in C_6D_6 at 298 K.

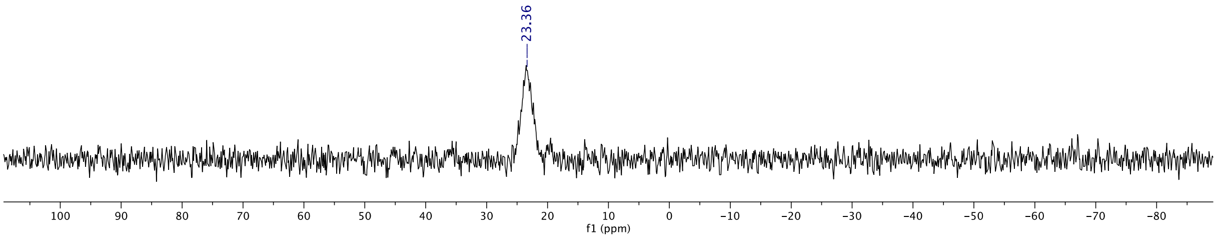

Figure 7. $^{11}B\{^{1}H\}$ NMR spectrum of 5 in C_6D_6at 298 K.

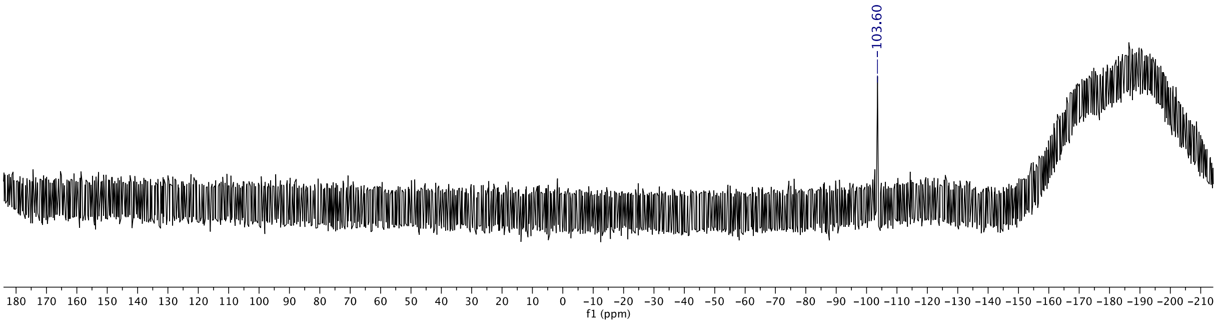

Figure 8. $^{19}F\{^{1}H\}$ NMR spectrum of 5 in C_6D_6 at 298 K.

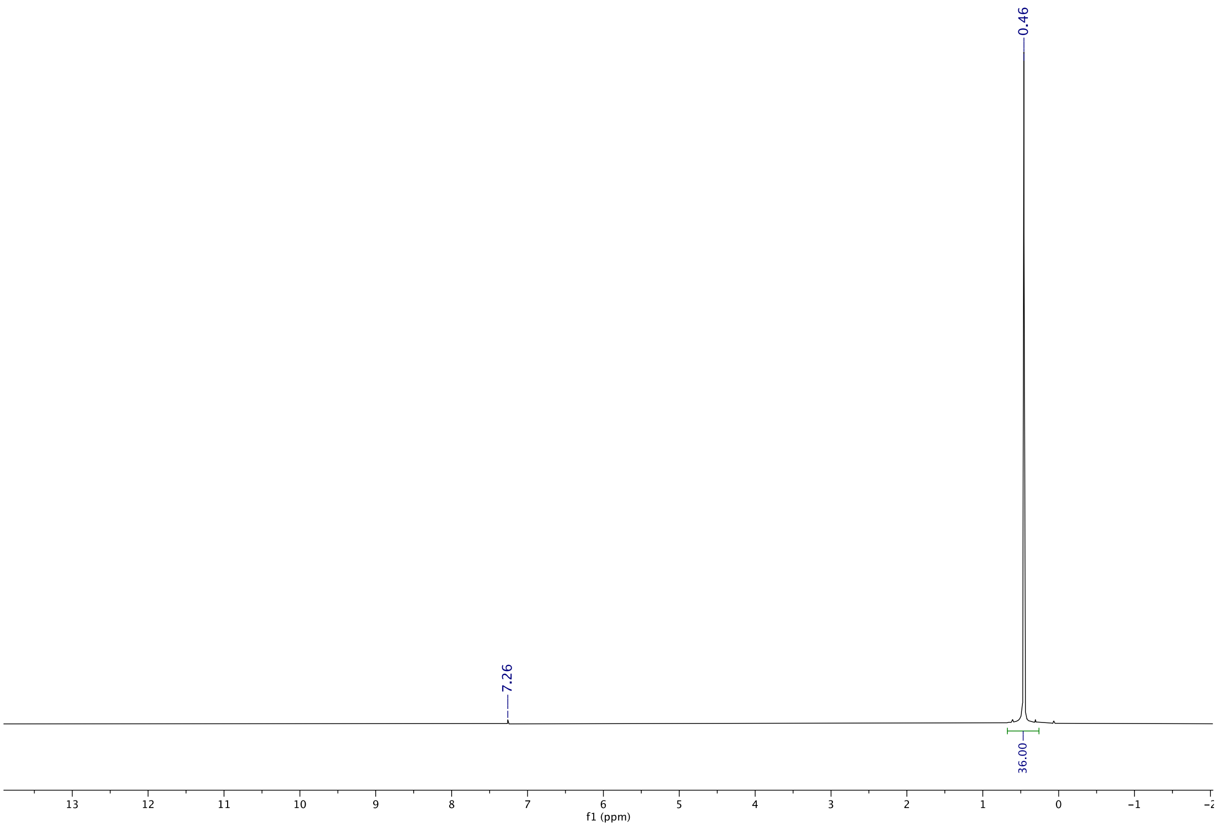

Figure 9. ^{1}H **NMR spectrum of 7 in** $CDCl_3$ **at 298 K.**

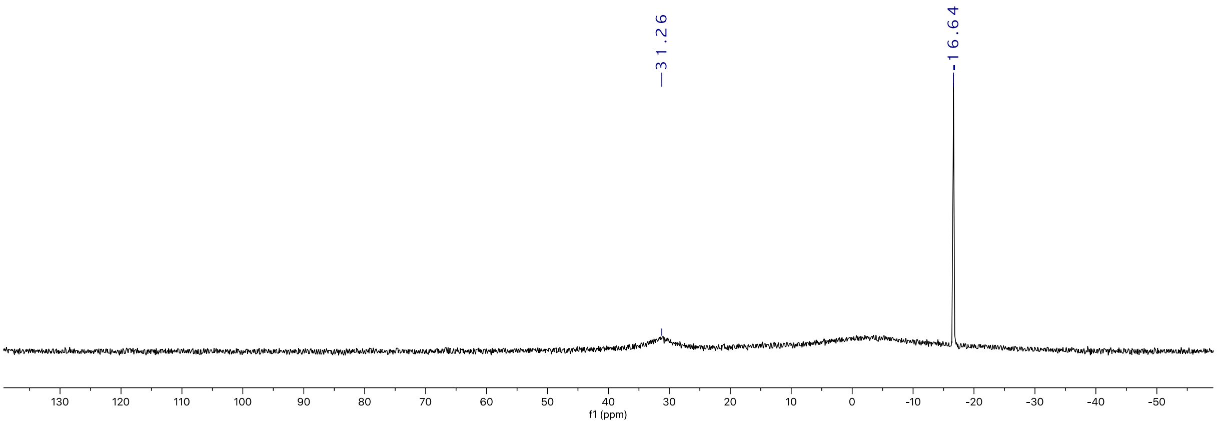

Figure 10. $^{11}B\{^{1}H\}$ **NMR spectrum of 7 in** $CDCl_3$ **at 298 K.**

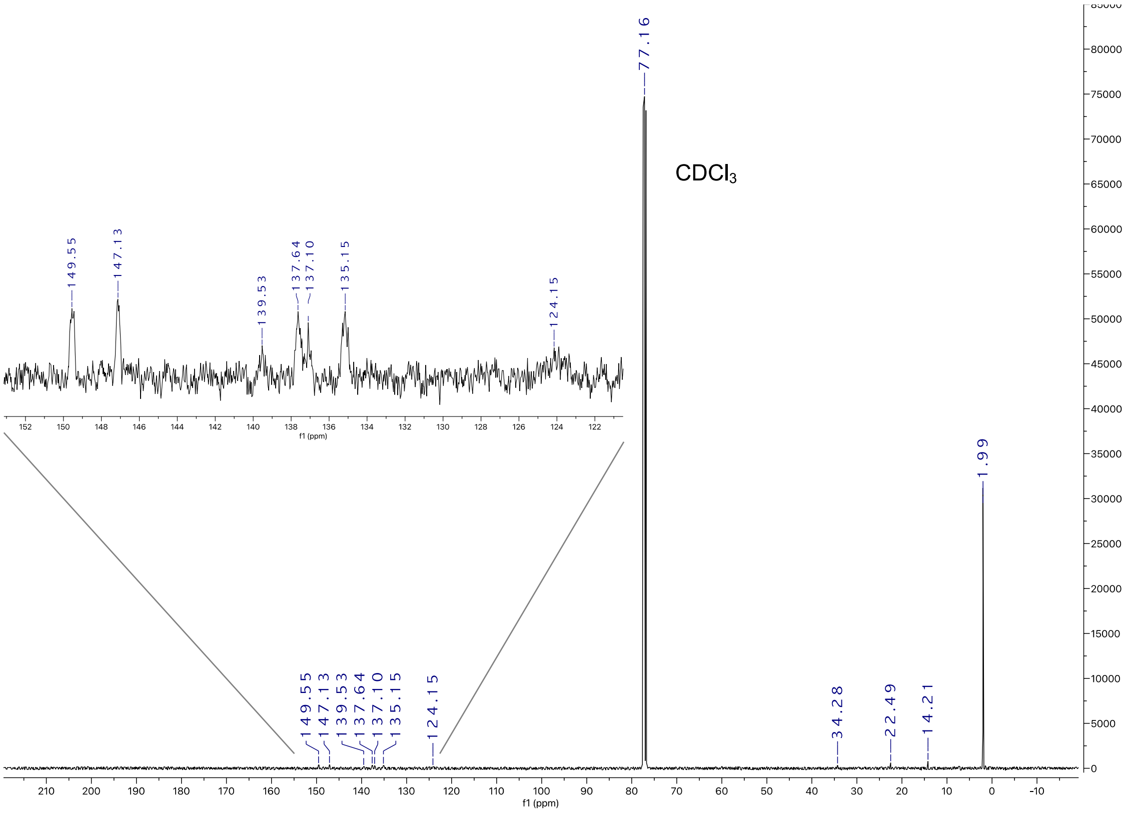

Figure 11. $^{13}C\{^{1}H\}$ **NMR spectrum of 7 in** $CDCl_3$ **at 298 K.**

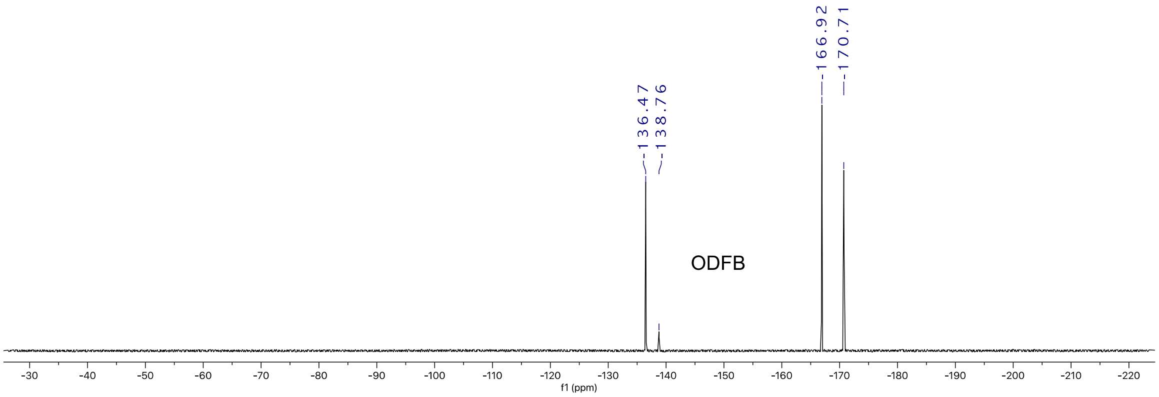

Figure 12. $^{19}F\{^{1}H\}$ **NMR spectrum of 7 in** $CDCl_3$**at 298 K.**

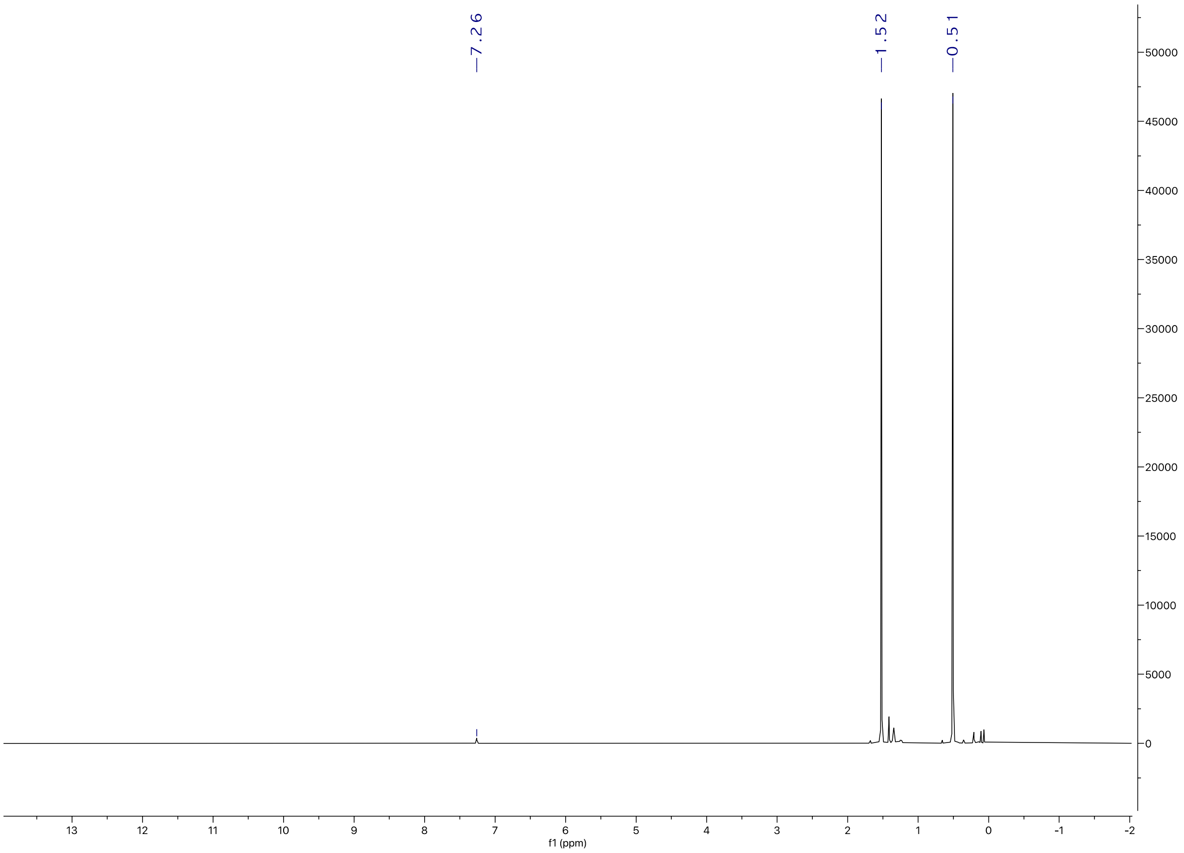

Figure 13. ^{1}H NMR spectrum of 8 in $CDCl_3$ at 298 K.

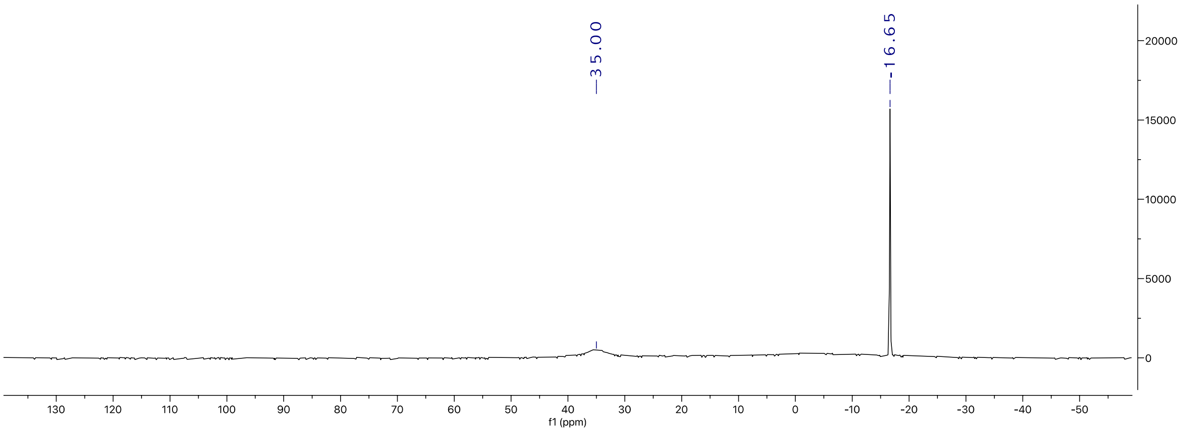

Figure 14. $^{11}B\{^{1}H\}$ NMR spectrum of 8 in $CDCl_3$ at 298 K.

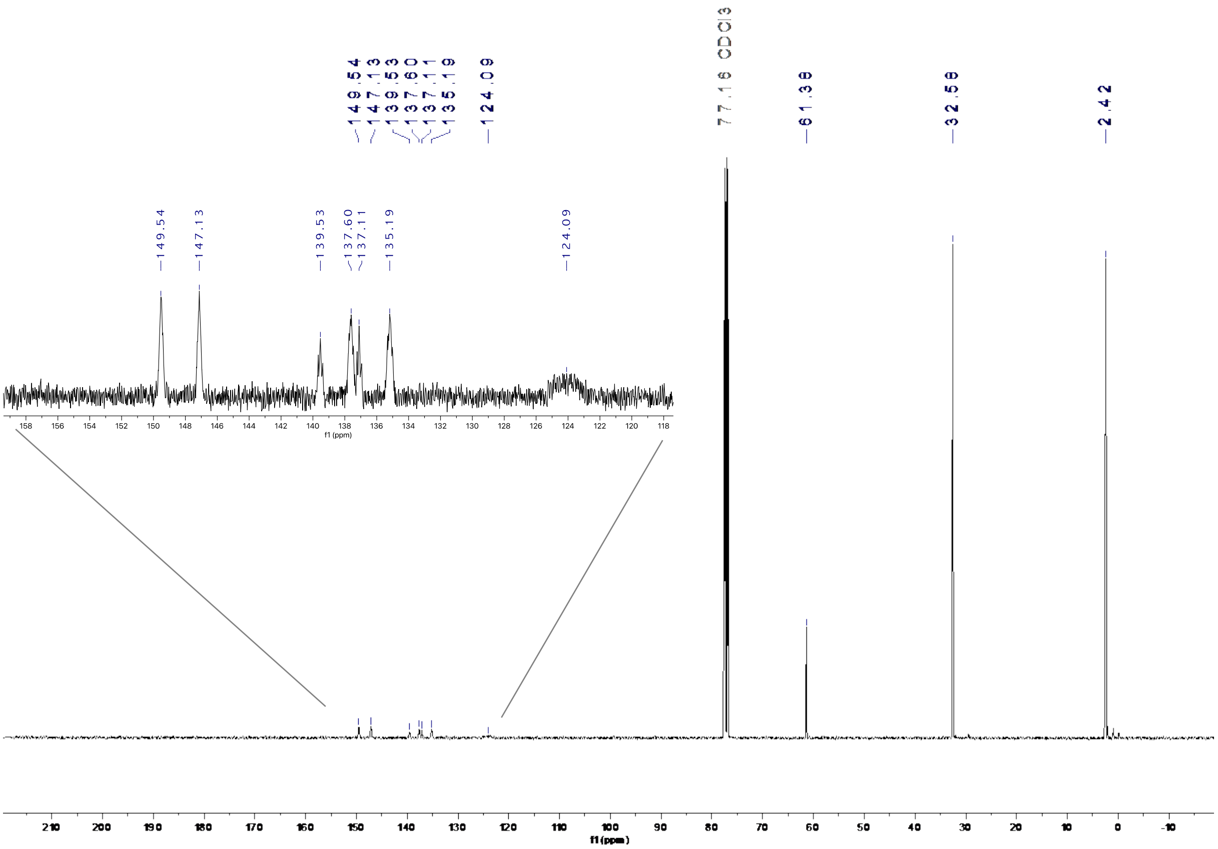

Figure 15. $^{13}C\{^{1}H\}$ **NMR spectrum of 8 in** $CDCl_3$ **at 298 K.**

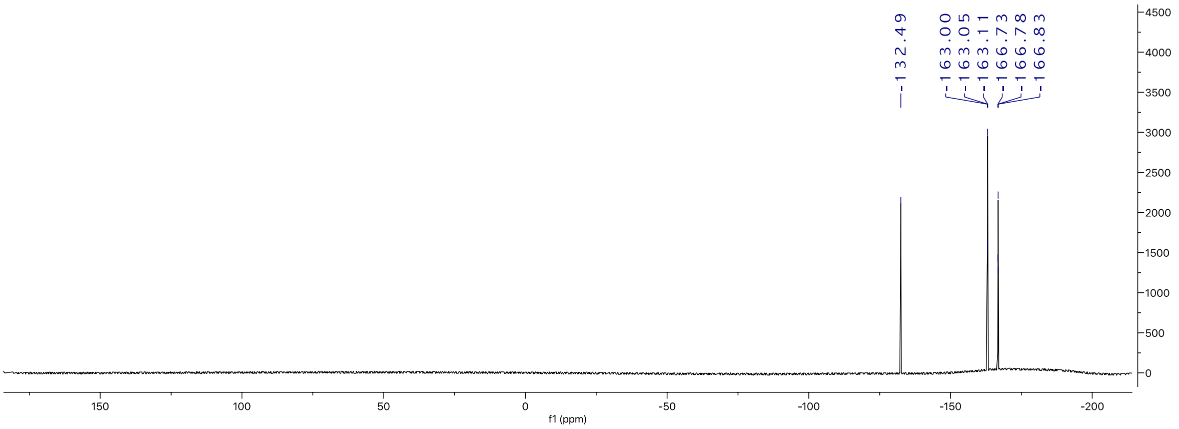

Figure 16. $^{19}F\{^{1}H\}$ **NMR spectrum of 8 in** $CDCl_3$ **at 298 K.**

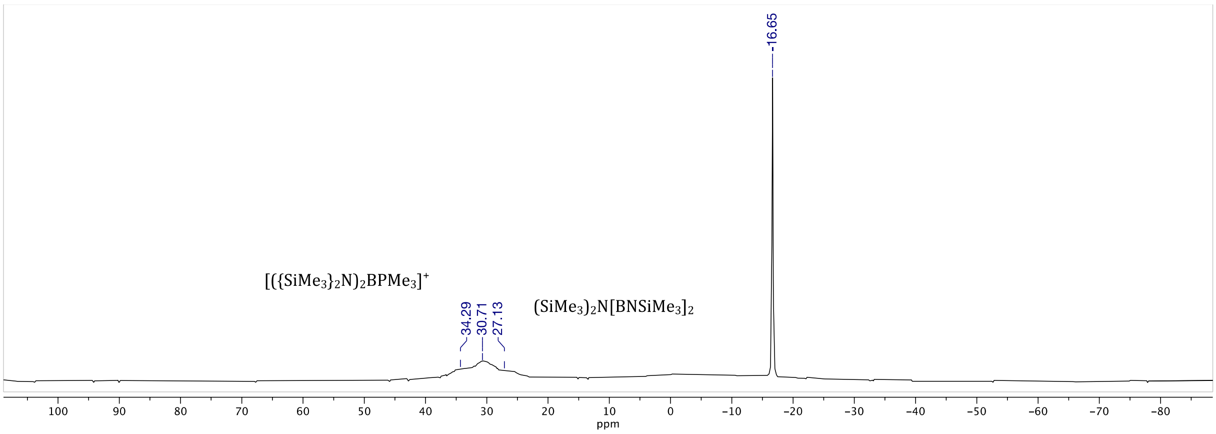

Figure 17. [11]**B{**[1]**H} NMR of [({SiMe3}2N)2B][B(C6F5)4] with PMe3 in $CDCl_3$ at 298 K.**

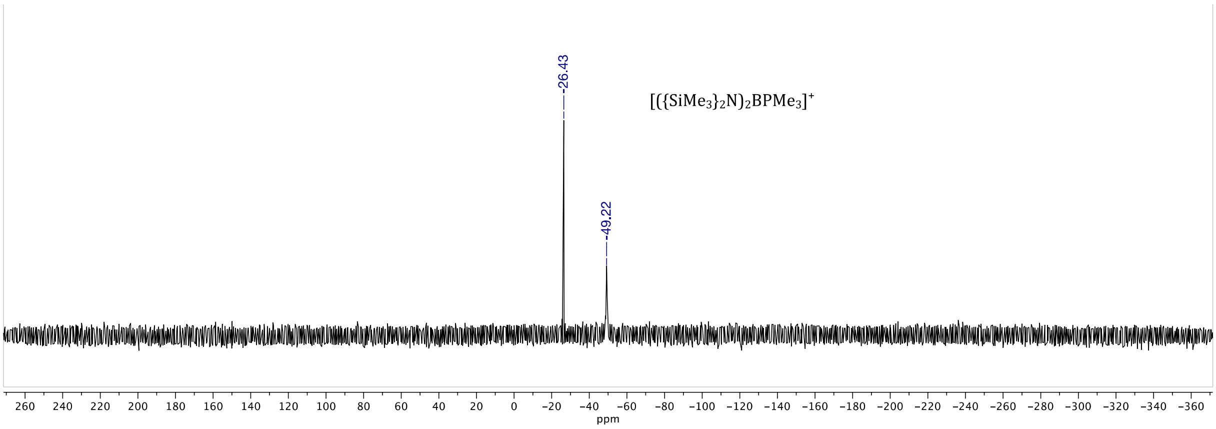

Figure 18. [31]**P{**[1]**H} NMR of [({SiMe3}2N)2B][B(C6F5)4] with PMe3 in $CDCl_3$ at 298 K.**

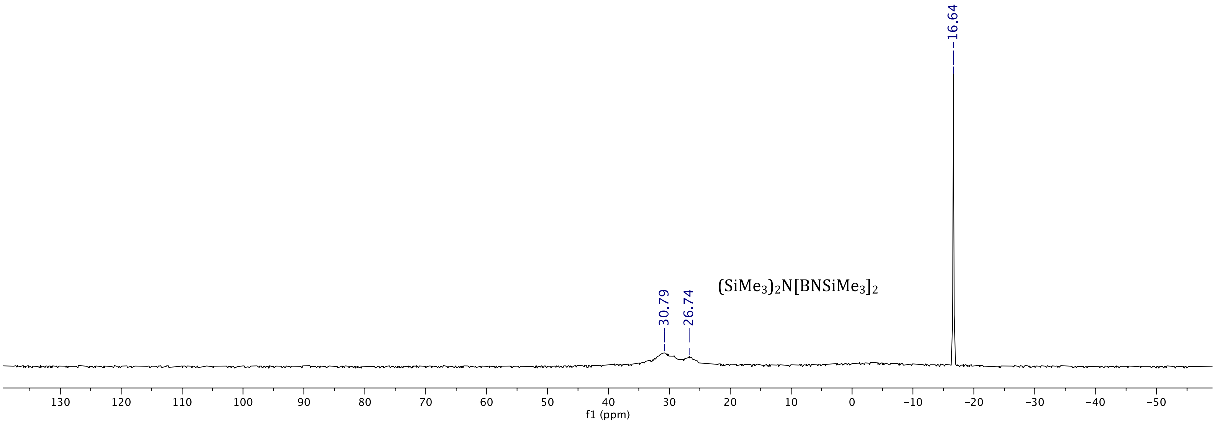

Figure 19. [11]**B{**[1]**H} NMR of [({SiMe3}2N)2B][B(C6F5)4] with PtBu3 in CH_2Cl_2 at 298 K.**

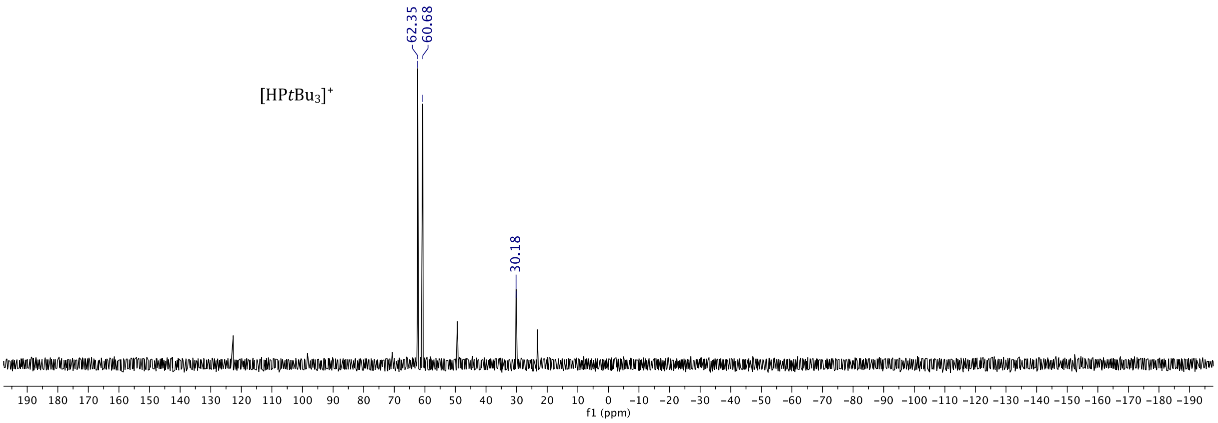

Figure 20. $^{31}P\{^1H\}$ NMR of [({SiMe3}2N)2B][B(C6F5)4] with PtBu3 in CH_2Cl_2 at 298 K.

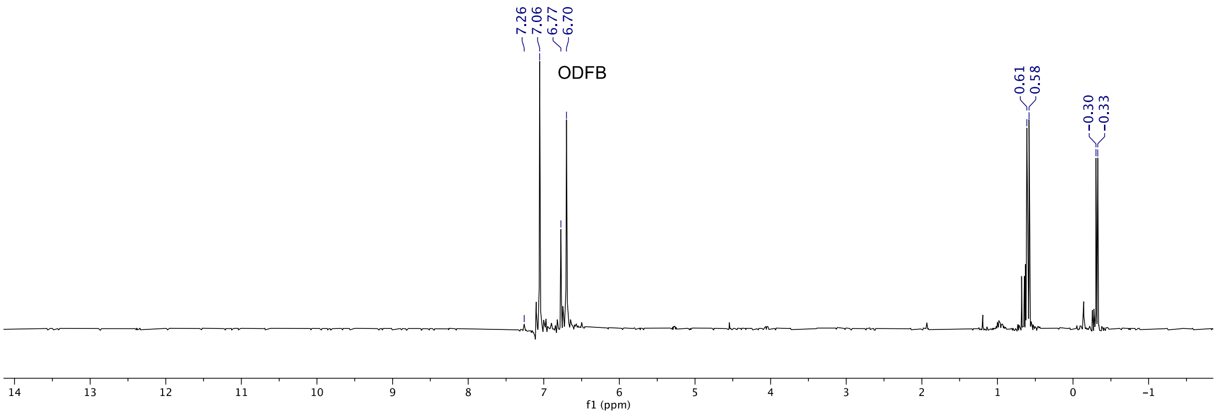

Figure 21. ^1H NMR spectrum of 9 in C_6D_5Br at 298 K.

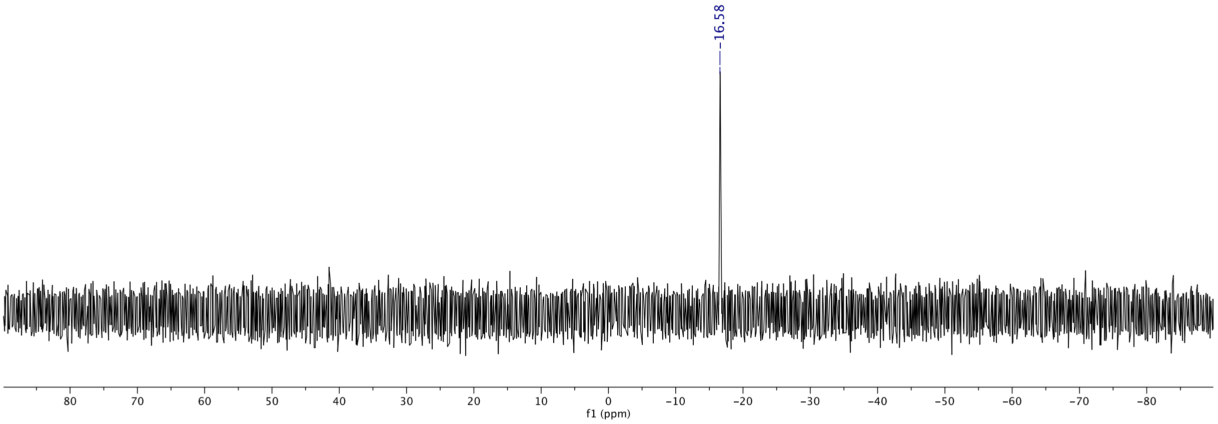

Figure 22. $^{11}B\{^1H\}$ NMR spectrum of 9 in C_6D_5Br at 298 K.

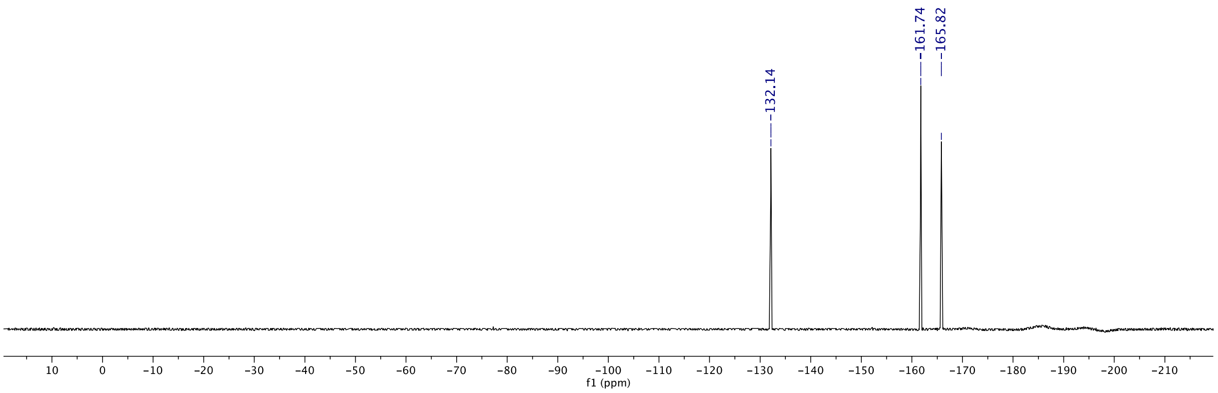

Figure 23. $^{19}F\{^{1}H\}$ NMR spectrum of 9 in C_6D_5Br at 298 K.

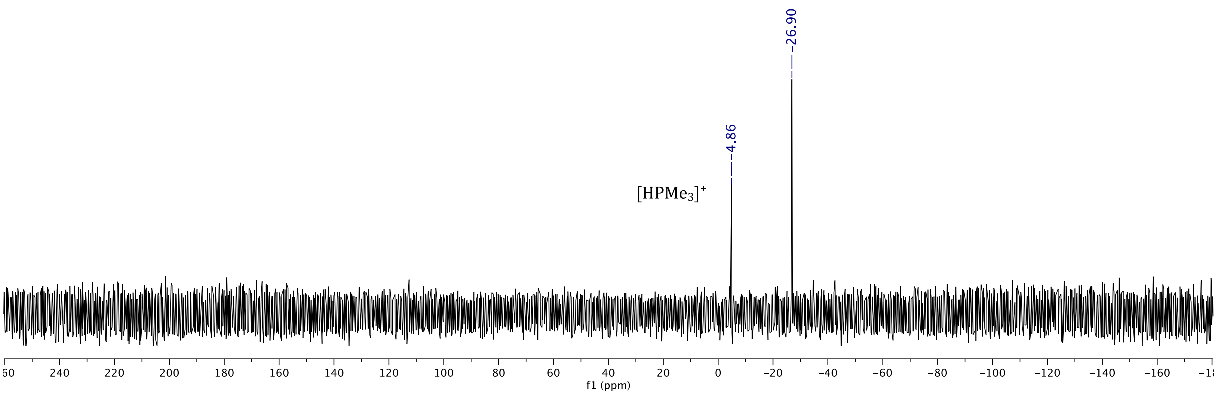

Figure 24. $^{31}P\{^{1}H\}$ NMR spectrum of 9 in C_6D_5Br at 298 K.

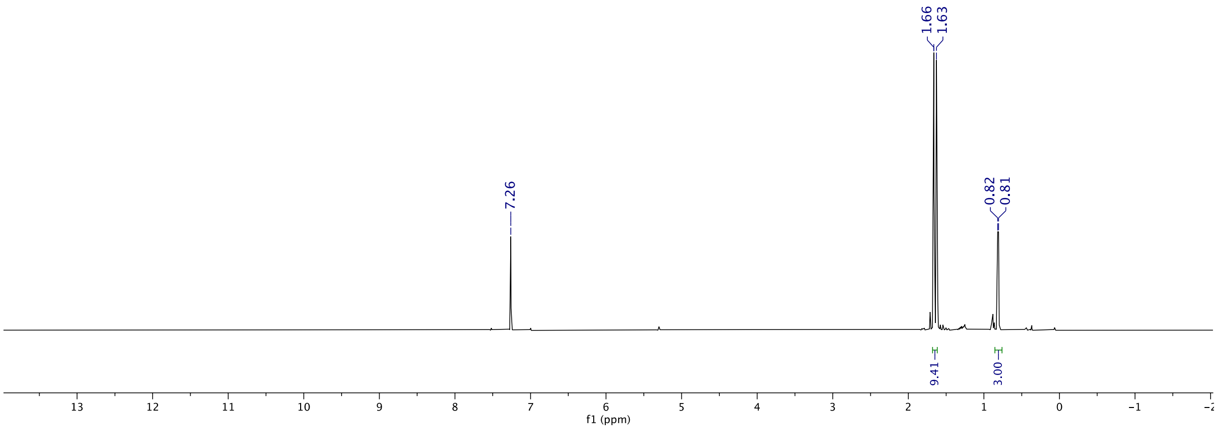

Figure 25. ^{1}H NMR spectrum of 11 in $CDCl_3$ at 298 K.

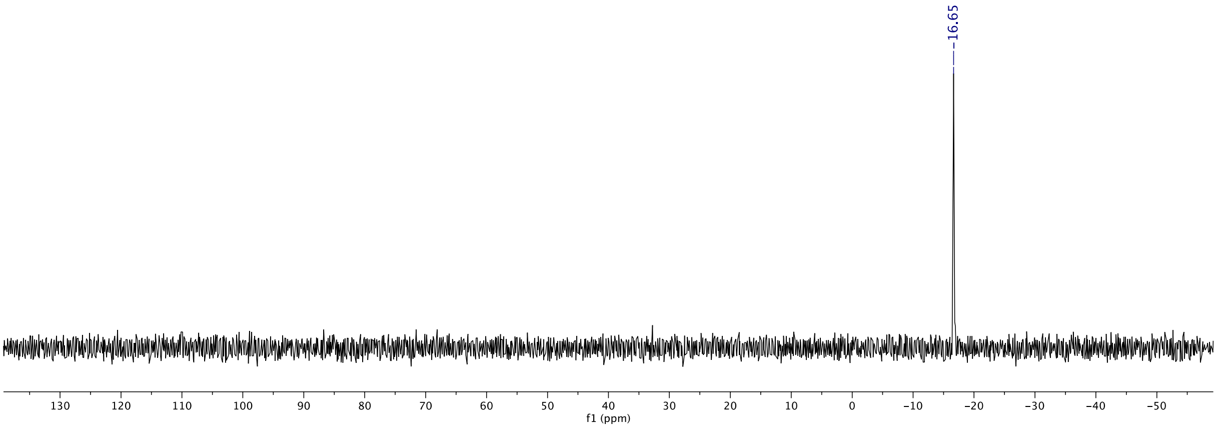

Figure 26. $^{11}B\{^1H\}$ NMR spectrum of 11 in $CDCl_3$ at 298 K.

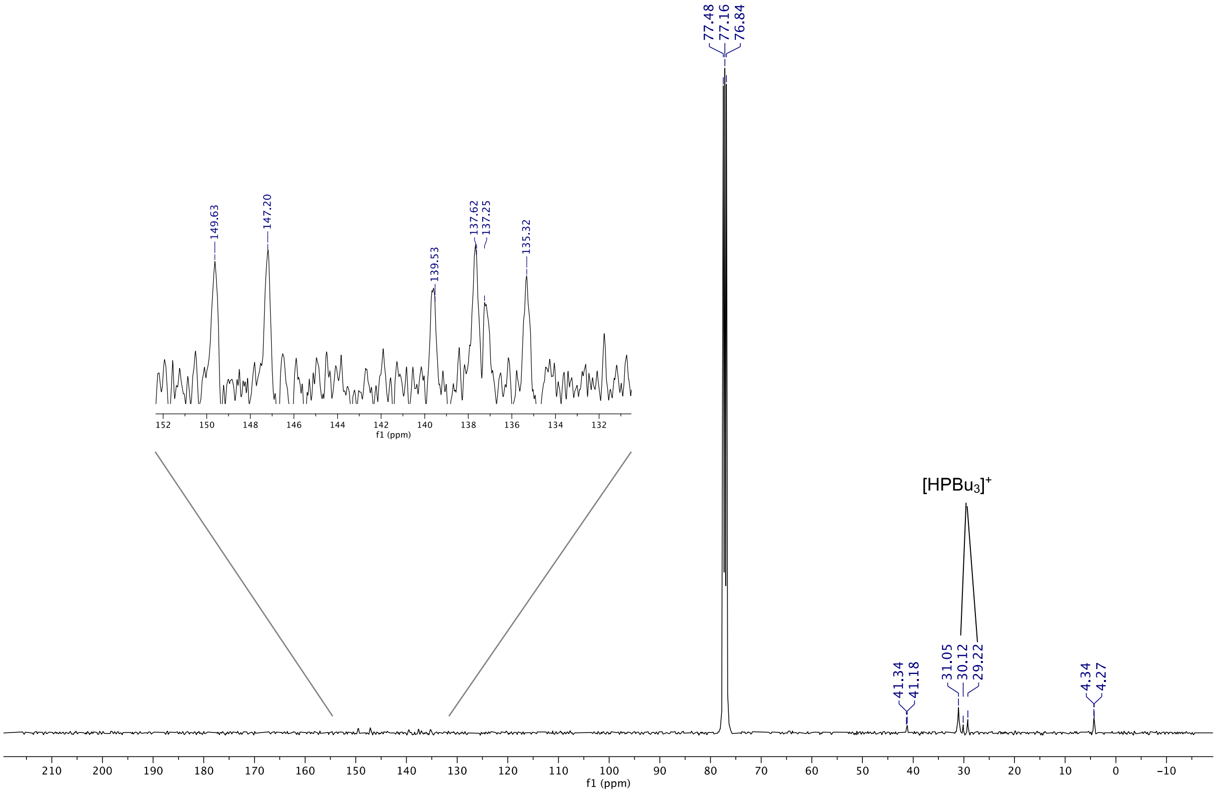

Figure 27. $^{13}C\{^1H\}$ NMR spectrum of 11 in $CDCl_3$ at 298 K. Exponential apodization of 10.0000 Hz applied to resolve -C_6F_5 signals.

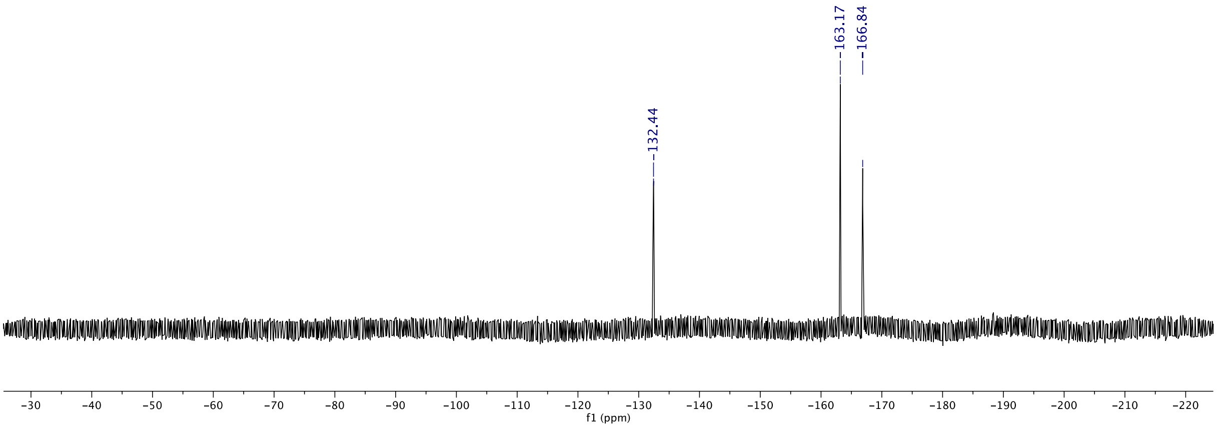

Figure 28. $^{19}F\{^{1}H\}$ **NMR spectrum of 11 in** $CDCl_3$ **at 298 K.**

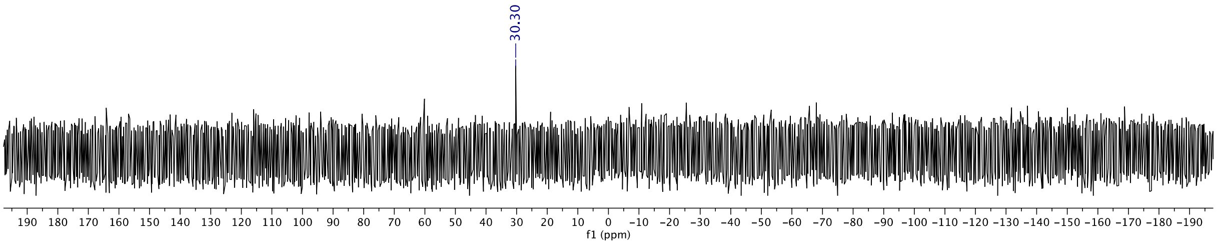

Figure 29. $^{31}P\{^{1}H\}$ **NMR spectrum of 11 in** $CDCl_3$ **at 298 K.**

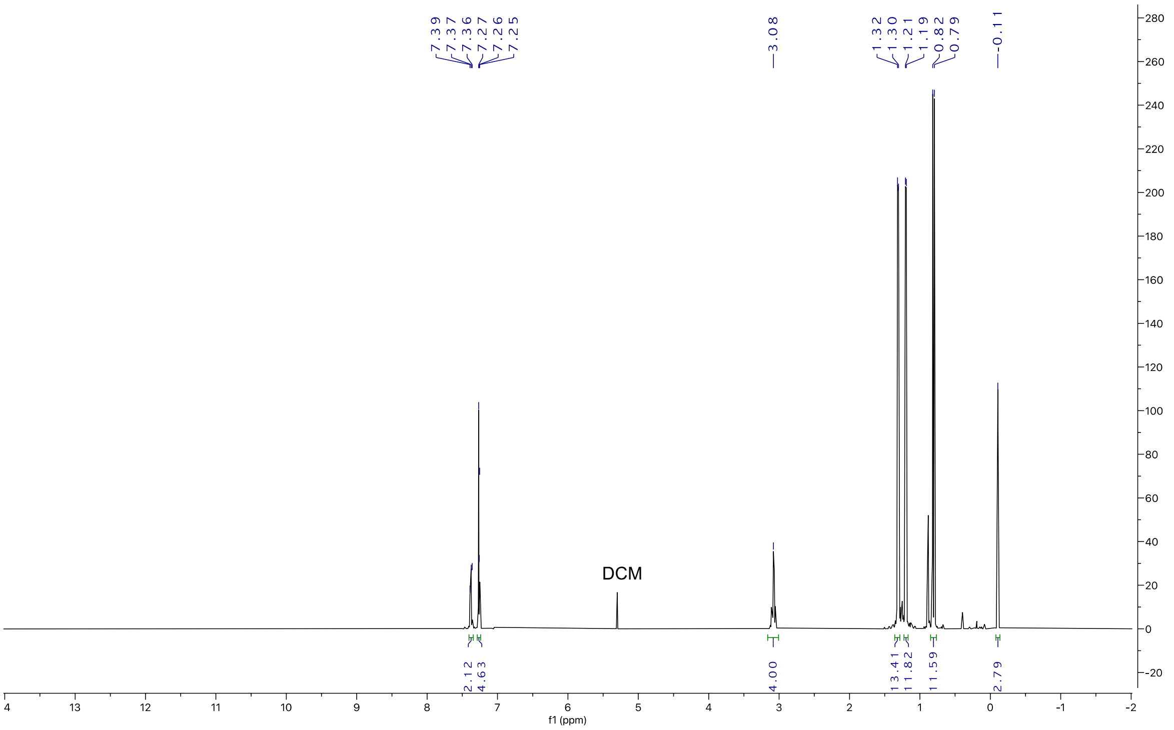

Figure 30. ^{1}H **NMR spectrum of 12 in** $CDCl_3$ **at 298 K.**

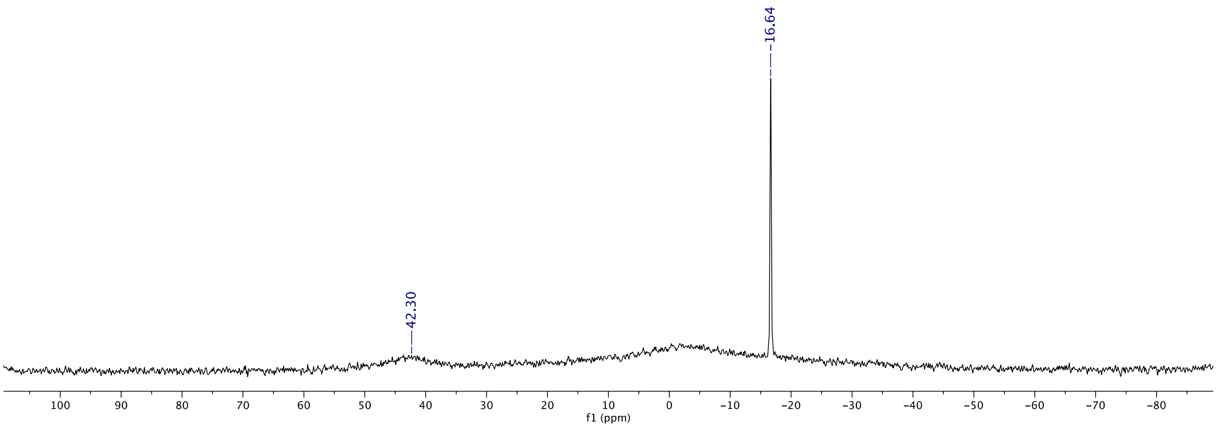

Figure 31. $^{11}B\{^{1}H\}$ **NMR spectrum of 12 in $CDCl_3$ at 298 K.**

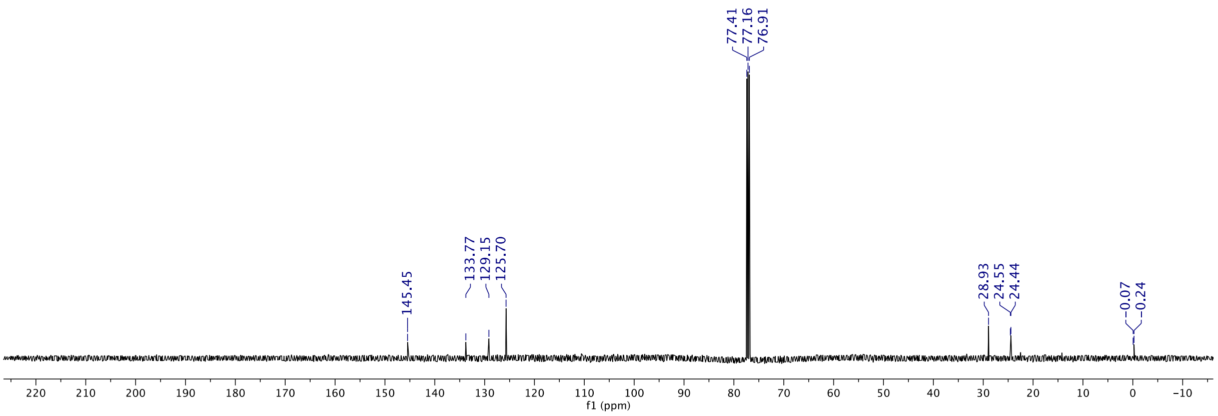

Figure 32. $^{13}C\{^{1}H\}$ **NMR spectrum of 12 in $CDCl_3$ at 298 K.**

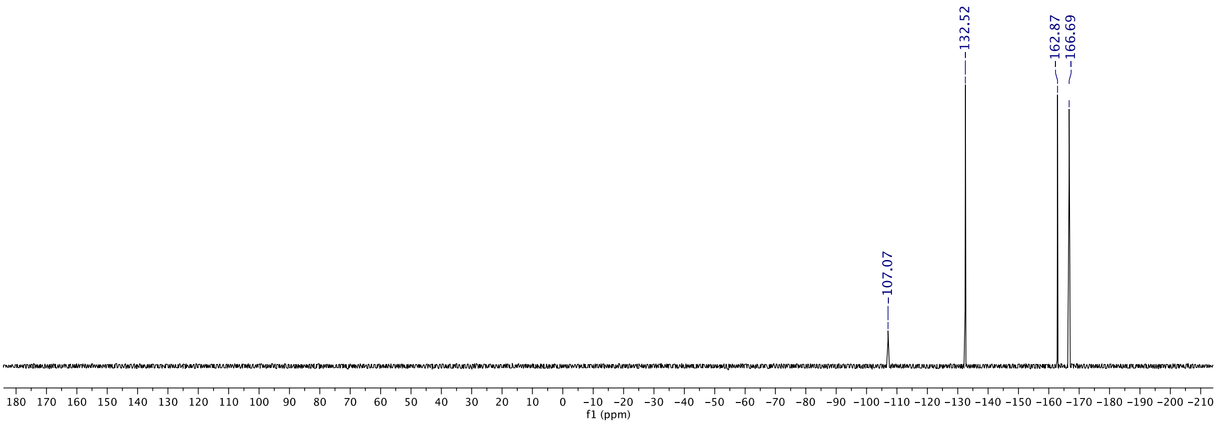

Figure 33. $^{19}F\{^{1}H\}$ **NMR spectrum of 12 in $CDCl_3$ at 298 K.**

Computational data

Computational Details:

The quantum chemical DFT calculations have been performed with the TURBOMOLE 7.4 suite of programs[1] The structures are fully optimized at the TPSS-D3/def2-TZVP + COSMO level of theory, which combines the TPSS meta-GGA density functional[2] with the BJ-damped DFT-D3 dispersion correction[3, 4] and the def2-TZVP basis set,[5, 6] using the Conductor-like Screening Model (COSMO) continuum solvation model[7] for $CHCl_3$ solvent (dielectric constant ε = 4.8 and solvent diameter R_{solv} = 3.17 Å). The density-fitting RI-J approach[5, 8, 9] is used to accelerate the geometry optimization and numerical harmonic frequency calculations[10] in solution. The optimized structures are characterized by frequency analysis to identify the nature of located stationary points (no imaginary frequency for true minima and only one imaginary frequency for transition state) and to provide thermal corrections (at 298.15 K and 1 atm) according to the modified ideal gas–rigid rotor–harmonic oscillator model.[11] This choice of dispersion-corrected meta-GGA functional makes the efficient exploration of all potential reaction paths possible.

The final solvation free energies in $CHCl_3$ are computed with the COSMO-RS solvation model[12] (parameter file: BP_TZVP_C30_1601.ctd) using the COSMOtherm program package[[13] on the above TPSS-D3 optimized structures, and corrected by +1.89 kcal·mol^{-1} to account for higher reference solute concentration of 1 mol·L^{-1} usually used in solution. To check the effects of the chosen DFT functional on the reaction energies and barriers, single-point calculations at the meta-GGA TPSS-D3[2] and hybrid-meta-GGA PW6B95-D3[14] levels are performed using a larger def2-QZVP basis set.[6, 15] The final reaction Gibbs free energies (ΔG) are determined from the electronic single-point energies plus TPSS-D3 thermal corrections and COSMO-RS solvation free energies. The computed relative free energies from both DFT functionals are mostly in very good overall agreement of about 0.2 ± 1.3 kcal/mol (average ± standard deviations). In our discussion, higher-level PW6B95-D3 Gibbs free energies (in kcal/mol, at 298.15 K and 1 mol/L concentration) will be used in our discussion unless specified otherwise. The applied DFT methods in combination with the large AO basis set provide usually accurate electronic energies leading to errors for chemical energies (including barriers) on the order of typically 1-2 kcal/mol. This has been tested thoroughly for the huge data base GMTKN55[16] which is the common standard in the field of DFT benchmarking.

Computational References

1 *TURBOMOLE V7.4*, **2019**, a development of University of Karlsruhe and Forschungszentrum Karlsruhe GmbH, 1989-2007, TURBOMOLE GmbH, since 2007; available from http://www.turbomole.com.

2 J. Tao, J. P. Perdew, V. N. Staroverov and G. E. Scuseria, *Phys. Rev. Lett.*, 2003, **91**, 146401.

3 S. Grimme, J. Antony, S. Ehrlich and H. Krieg, *J. Chem. Phys.*, 2010, **132**, 154104-154119.

4 S. Grimme, S. Ehrlich and L. Goerigk, *J. Comput. Chem.*, 2011, **32**, 1456-1465.

5 F. Weigend, M. Häser, H. Patzelt and R. Ahlrichs, *Chem. Phys. Lett.*, 1998, **294**, 143-152.

6 F. Weigend and R. Ahlrichs, *Phys. Chem. Chem. Phys.*, 2005, **7**, 3297-3305.

7 A. Klamt and G. Schüürmann, *J. Chem. Soc., Perkin Trans. 2*, 1993, 799-805.

8 K. Eichkorn, F. Weigend, O. Treutler and R. Ahlrichs, *Theor. Chem. Acc.*, 1997, **97**, 119-124.

9 F. Weigend, *Phys. Chem. Chem. Phys.*, 2006, **8**, 1057-1065.

10 P. Deglmann, K. May, F. Furche and R. Ahlrichs, *Chem. Phys. Lett.*, 2004, **384**, 103-107.
11 S. Grimme, *Chem. - Eur. J.*, 2012, **18**, 9955-9964.
12 F. Eckert and A. Klamt, *AIChE J.*, 2002, **48**, 369-385.
13 F. Eckert and A. Klamt, COSMOtherm, Version C3.0, Release 16.01; COSMOlogic GmbH & Co. KG, Leverkusen, Germany 2015.
14 Y. Zhao and D. G. Truhlar, *J. Phys. Chem. A*, 2005, **109**, 5656-5667.
15 F. Weigend, F. Furche and R. Ahlrichs, *J. Chem. Phys.*, 2003, **119**, 12753-12762.
16 L. Goerigk, A. Hansen, C. Bauer, S. Ehrlich, A. Najibi, S. Grimme, *Phys. Chem. Chem. Phys.* **2017**, *19*, 32184-32215.

Table S1. TPSS-D3/def2-TZVP + COSMO computed imaginary frequency (ImF), zero-point energies (ZPE), gas-phase enthalpic (Hc) and Gibbs free-energy (Gc) corrections; the COSMO-RS computed solvation enthalpic (Hsol) and Gibbs free-energy (Gsol) corrections in THF solution; TPSS-D3/def2-QZVP and PW6B95-D3/def2-QZVP single-point energies (TPSS-D3 and PW6B95-D3); the total PW6B95-D3 free energies G_P; the relative electronic energies (ΔE_T and ΔE_P) and Gibbs free-energies (ΔG_T and ΔG_P) at the TPSS-D3 and PW6B95-D3 levels.

Reactions in $CHCl_3$ solution	Im cm^{-1}	ZPE kcal/mol	Hc kcal/mol	Gc kcal/mol	Hsol kcal/mol	Gsol kcal/mol	TPSS-D3 E_h	PW6B95-D3 E_h	G_P E_h	ΔE_T kcal/mol	ΔE_P kcal/mol	*ΔG_P kcal/mol*	ΔG_T kcal/mol
Silylium cation Et_3Si^+ is -10.1 kcal/mol bound to toluene													
PhMe (toluene)	0	79.99	84.17	61.70	-8.22	-5.25	-271.74572	-272.04253	-271.94956				
Et_3Si^+ + PhMe	0	202.49	213.94	162.95	-59.67	-52.08	-798.87434	-799.65462	-799.47193	0.00	0.00	*0.00*	0.00
$Et_3Si(tol)^+$	0	204.36	215.78	178.09	-48.31	-42.99	-798.92905	-799.70637	-799.48808	-34.33	-32.47	*-10.13*	-11.99
..but not bound to $B(C_6F_5)_4^-$ anion in chloroform solution													
Et_3Si^+ + $B(C_6F_5)_4^-$	0	246.33	278.67	185.66	-95.14	-86.50	-3465.12303	-3468.60599	-3468.44194	0.00	0.00	*0.00*	0.00
$Et_3Si.B(C_6F_5)_4$	0	248.57	280.86	202.01	-36.91	-30.56	-3465.23312	-3468.71517	-3468.43893	-69.08	-68.52	*1.89*	1.32
*The Silylium $Et_3Si(toluene)^+$ may rapidly abstract fluoride F^- from **1***													
**1** + $Et_3Si(tol)^+$	0	497.39	529.22	437.60	-59.98	-49.22	-2671.15945	-2673.62743	-2673.00248	0.00	0.00	*0.00*	0.00
**1.**$Et_3Si(tol)^+$	0	494.17	528.62	446.05	-51.37	-42.55	-2671.17150	-2673.64140	-2672.99538	-7.56	-8.77	*4.46*	5.66
**TS1**$^+$	140i	494.73	528.52	447.59	-47.55	-39.00	-2671.16709	-2673.63766	-2672.98352	-4.79	-6.42	*11.90*	13.53
**7**$^+$ + Et_3SiF + PhMe	0	495.47	527.76	421.52	-59.77	-46.53	-2671.17773	-2673.64648	-2673.03985	-11.47	-11.95	*-23.46*	-22.97
*..or abstract a methyl group at first followed by facile F-shift to form bori **7F**$^+$*													
**1** + $Et_3Si(tol)^+$	0	497.39	529.22	437.60	-59.98	-49.22	-2671.15945	-2673.62743	-2673.00248	0.00	0.00	*0.00*	0.00
**TS2**$^+$	79i	494.33	528.18	446.94	-50.84	-41.68	-2671.16229	-2673.63116	-2672.98234	-1.78	-2.35	*12.64*	13.20
**1.**Et_3Si^+ + PhMe	0	497.18	529.29	437.79	-53.30	-42.62	-2671.14989	-2673.61794	-2672.98216	6.00	5.95	*12.75*	12.80
**A**$^+$ + Et_3SiMe + PhMe	0	495.42	527.36	420.96	-62.12	-49.30	-2671.13570	-2673.60362	-2673.00231	14.90	14.94	*0.10*	0.07
**TS3**$^+$ + Et_3SiMe + PhMe	88i	492.83	525.78	416.72	-62.74	-49.90	-2671.12516	-2673.59463	-2673.00102	21.52	20.58	*0.91*	1.85
**7F**$^+$ + Et_3SiMe + PhMe	0	495.43	527.70	420.61	-58.63	-45.98	-2671.17525	-2673.64504	-2673.03899	-9.91	-11.05	*-22.91*	-21.77
*Direct P-B adduct of **7**$^+$ and PMe_3 is kinetically accessible but thermodynamically unstable*													
**7**$^+$ + PMe_3	0	361.40	385.98	311.19	-53.99	-42.20	-2233.28874	-2235.26628	-2234.83160	0.00	0.00	*0.00*	0.00
**TSB**$^+$	48i	359.51	385.32	319.99	-44.19	-37.18	-2233.29643	-2235.27357	-2234.81987	-4.83	-4.57	*7.36*	7.10
**B**$^+$	0	363.79	387.87	326.89	-45.68	-38.74	-2233.30621	-2235.28304	-2234.82084	-10.96	-10.51	*6.75*	6.30
*..while Me_3Si^+ abstraction from borinium **7**$^+$ with PMe_3 is -12.7 kcal/mol exergonic over a low barrier of only 8.9 kcal/mol*													

**TS4**$^+$	31i	358.88	385.23	318.38	-48.73	-41.06	-2233.28761	-2235.26237	-2234.81743	0.70	2.45	*8.89*	7.14
**C** + **9**$^+$	0	361.83	386.32	309.40	-61.73	-51.34	-2233.26589	-2235.24148	-2234.82422	14.33	15.57	*4.63*	3.40
0.5***10** + **9**$^+$	0	362.79	387.13	318.94	-56.47	-48.25	-2233.30987	-2235.28768	-2234.85180	-13.26	-13.43	*-12.67*	-12.51

*..proton abstraction from **7**$^+$ with PMe_3 is 1.3 kcal/mol endergonic over a sizable barrier of 24.0 kcal/mol*

**7**$^+$ + PMe_3	0	361.40	385.98	311.19	-53.99	-42.20	-2233.28874	-2235.26628	-2234.83160	0.00	0.00	*0.00*	0.00
**TS5**$^+$	888i	355.98	381.89	316.06	-47.14	-39.73	-2233.26406	-2235.23678	-2234.79341	15.48	18.52	*23.96*	20.93
**10C** + PMe_3H^+	0	362.57	386.29	312.32	-65.70	-56.50	-2233.26510	-2235.24319	-2234.82950	14.83	14.49	*1.32*	1.66

*Me_3Si^+ abstraction from borinium **7**$^+$ with tBu_3P is -13.6 kcal/mol exergonic over a barrier of 18.6 kcal/mol to form $tBu_3PSiMe_3^+$ (**11**$^+$)*

**7**$^+$ + *t*Bu_3P	0	519.93	551.50	462.44	-58.83	-45.88	-2587.32274	-2589.67382	-2589.00398	0.00	0.00	*0.00*	0.00
**TS4t**$^+$	157i	518.66	551.65	473.55	-46.17	-37.88	-2587.32381	-2589.67157	-2588.97426	-0.67	1.41	*18.64*	16.56
**C** + **11**$^+$	0	521.50	552.69	462.68	-59.44	-48.21	-2587.31341	-2589.66460	-2588.99808	5.85	5.79	*3.70*	3.77
0.5***10** + **11**$^+$	0	522.46	553.51	472.22	-54.18	-45.12	-2587.35738	-2589.71081	-2589.02565	-21.74	-23.21	*-13.60*	-12.13

*..while proton abstraction from **7**$^+$ with tBu_3P is -5.0 kcal/mol exergonic but over a sizable barrier of 25.7 kcal/mol*

**7**$^+$ + *t*Bu_3P	0	519.93	551.50	462.44	-58.83	-45.88	-2587.32274	-2589.67382	-2589.00398	0.00	0.00	*0.00*	0.00
**TS5t**$^+$	945i	514.20	547.47	468.54	-46.67	-38.54	-2587.30643	-2589.65134	-2588.96307	10.23	14.11	*25.67*	21.79
**10C** + *t*Bu_3PH^+	0	520.89	551.50	463.46	-60.00	-49.66	-2587.32704	-2589.67742	-2589.01198	-2.70	-2.26	*-5.02*	-5.47

*F^- abstraction from Dipp-substituted **6** with $Et_3Si(tol)^+$ is -10.1 kcal/mol exergonic over a sizable barrier of 22.8 kcal/mol*

**6** + $Et_3Si(tol)^+$	0	678.38	718.17	611.04	-70.42	-58.29	-2787.98717	-2790.74402	-2789.85712	0.00	0.00	*0.00*	0.00
**TS6a**$^+$	138i	677.16	718.09	624.17	-55.89	-46.18	-2787.98505	-2790.74487	-2789.82077	1.33	-0.53	*22.82*	24.68
**Da**$^+$ + Et_3SiF + PhMe	0	676.45	716.61	594.59	-65.71	-51.32	-2787.99216	-2790.74808	-2789.87329	-3.13	-2.55	*-10.14*	-10.72

..leading to selective and facile F-aided methyl abstrcation from 6 with $Et_3Si(tol)^+$

**TS6**$^+$	71i	675.34	717.28	620.26	-57.92	-47.89	-2788.00099	-2790.75900	-2789.84387	-8.67	-9.40	*8.32*	9.05
**D**$^+$ + Et_3SiMe + PhMe	0	676.79	716.79	594.81	-64.97	-51.11	-2787.98917	-2790.74672	-2789.87124	-1.25	-1.70	*-8.86*	-8.41

*..followed by facile Si-to-B methyl shift and rapid ring-closing via B-N bond rotation, forming Si-F-Si bridged **12**$^+$*

**6** + $Et_3Si(tol)^+$ - Et_3SiMe - PhMe	0	452.82	479.91	426.82	-55.25	-49.29	-1948.88014	-1950.81587	-1950.21423	0.00	0.00	*0.00*	0.00
**TS7**$^+$	239i	448.65	476.39	407.32	-52.54	-44.70	-1948.85199	-1950.78817	-1950.20728	17.67	17.38	*4.36*	4.64
**E**$^+$	0	453.65	479.84	414.48	-50.60	-42.95	-1948.89219	-1950.82657	-1950.23149	-7.56	-6.71	*-10.83*	-11.67
**TS8**$^+$	26i	449.36	476.62	408.46	-51.59	-43.72	-1948.87029	-1950.80439	-1950.22013	6.18	7.21	*-3.71*	-4.73
**12**$^+$	0	452.17	478.95	412.11	-52.62	-44.69	-1948.89791	-1950.83599	-1950.24746	-11.15	-12.62	*-20.86*	-19.38

Table S2. TPSS-D3/def2-TZVP + COSMO optimized Cartesian coordinates (in Å) in $CHCl_3$ solution. Each structure is labeled by the specific name (See Table S1), followed by the number of atoms, the total energy (in hartrees), and the detailed atomic coordinates (in double-column text list).

10C : cyclic *c*-N($SiMe_3$)$SiMe_2CH_2$B-N($SiMe_3$)$_2$
54
Energy = -1771.564961161

B	0.4320535	-0.0887631	-0.6701784
N	0.3150908	1.2035697	0.0025996
N	-0.2571753	-1.3125334	-0.3337914
Si	1.0987038	1.4272434	1.5523449
Si	-0.2674862	2.5180159	-1.0023712
Si	-1.5517716	-1.7416720	0.7620514
Si	0.6645739	-2.1824879	-1.5815341
C	2.1959298	-0.0667771	1.8872171
C	2.2177652	2.9446793	1.5169200
C	-0.1282581	1.6575533	2.9640035
C	-1.1588990	3.7863747	0.0660724
C	1.1175560	3.3604281	-1.9617735
C	-1.5053774	1.8119713	-2.2349544
C	-2.7713774	-0.3212465	0.9223812
C	-2.4379475	-3.2415605	0.0482449
C	-0.8772562	-2.2317058	2.4511676
C	1.8031021	-3.4818825	-0.8451058
C	-0.3981475	-2.9365222	-2.9339499
C	1.3777322	-0.4882944	-1.9235146
H	1.6387785	-1.0079427	1.8843897
H	2.9842054	-0.1422241	1.1279274
H	2.6834107	0.0345204	2.8648416
H	2.7204273	3.0552944	2.4864335
H	1.6700011	3.8737990	1.3245427
H	2.9908168	2.8435173	0.7464734
H	-0.7609559	2.5368234	2.7968235
H	0.4070425	1.8068820	3.9106634
H	-0.7878597	0.7926624	3.0857463
H	-2.0035708	3.3287656	0.5938594
H	-1.5541727	4.5912475	-0.5666118
H	-0.5040891	4.2478841	0.8131274
H	1.6627925	2.6289507	-2.5697310
H	1.8378685	3.8430570	-1.2922779
H	0.7159863	4.1284503	-2.6352749
H	-1.9035089	2.6109962	-2.8729177
H	-1.0506367	1.0615237	-2.8918600
H	-2.3485017	1.3390910	-1.7195982
H	-2.2650796	0.6122114	1.1850588
H	-3.3000520	-0.1612798	-0.0245872
H	-3.5211337	-0.5368430	1.6936564
H	-3.2618400	-3.5456521	0.7058732
H	-1.7629639	-4.1006725	-0.0515562
H	-2.8594809	-3.0270007	-0.9403969
H	-0.1388777	-3.0372641	2.3547099
H	-1.6898053	-2.5982346	3.0914279
H	-0.3948489	-1.3982184	2.9706408
H	2.4459698	-3.9286687	-1.6133269
H	1.2253467	-4.2923837	-0.3827613
H	2.4460259	-3.0432348	-0.0736592
H	-1.1229112	-2.2064967	-3.3123606
H	0.2330718	-3.2534171	-3.7739253
H	-0.9507789	-3.8148810	-2.5818752
H	2.4459395	-0.3094627	-1.7610551
H	1.0995842	-0.0386591	-2.8834655

10 : cyclic dimer [(Me_3Si)$_2$NB(N$SiMe_3$)]$_2$
84
Energy = -2725.579836584

B	-0.9708126	-0.0088535	0.0026172
N	-2.4168465	-0.0201653	0.0026499
N	0.0092145	-1.1073843	0.0123323
Si	-3.2395180	0.4815174	1.4730174
Si	-3.2340478	-0.5374034	-1.4650682
Si	0.0253645	-2.8109160	0.3421687
C	-1.9979246	0.4190456	2.8832558
C	-3.9441891	2.2271808	1.3926186
C	-4.6637524	-0.6955091	1.8329317
C	-3.9056068	-2.2961255	-1.3846072
C	-4.6820113	0.6119794	-1.8196515
C	-1.9976955	-0.4512041	-2.8785957
C	-1.4920484	-3.2787421	1.3510389
C	0.0329926	-3.7894020	-1.2689724
C	1.5526621	-3.2513739	1.3484582
H	-1.5659345	-0.5801546	3.0023091
H	-1.1735332	1.1204396	2.7178264
H	-2.4849198	0.6919269	3.8275429
H	-4.4970266	2.4390417	2.3172720
H	-4.6365431	2.3573159	0.5540566
H	-3.1565279	2.9818700	1.2989717
H	-5.4229097	-0.6826871	1.0424511
H	-5.1589187	-0.4008480	2.7668466
H	-4.3120531	-1.7265203	1.9476797

H	-4.4565010	-2.5165922	-2.3084430
H	-4.5938101	-2.4399783	-0.5448426
H	-3.1043018	-3.0366472	-1.2937587
H	-4.3513731	1.6499158	-1.9343749
H	-5.4386620	0.5830463	-1.0272300
H	-5.1736233	0.3083252	-2.7525864
H	-2.4803180	-0.7382970	-3.8209079
H	-1.5882699	0.5569091	-3.0019170
H	-1.1575070	-1.1335037	-2.7124726
H	-1.4389180	-2.8412702	2.3547293
H	-2.4177915	-2.9311828	0.8851259
H	-1.5566841	-4.3681522	1.4652743
H	0.0399495	-4.8693356	-1.0752114
H	0.9152743	-3.5536580	-1.8755753
H	-0.8520288	-3.5654757	-1.8760828
H	2.4703708	-2.8831862	0.8825138
H	1.6396762	-4.3396434	1.4585970
H	1.4919727	-2.8188507	2.3538338
B	0.9710766	0.0073136	0.0001939
N	2.4170199	0.0202051	-0.0030442
N	-0.0092504	1.1059460	-0.0084204
Si	3.2398357	-0.4807651	-1.4734070
Si	3.2346164	0.5355305	1.4656035
Si	-0.0237213	2.8089614	-0.3406225
C	1.9980609	-0.4169262	-2.8832873
C	4.6652900	0.6950219	-1.8321733
C	3.9442721	-2.2266598	-1.3939550
C	4.6813995	-0.6148820	1.8216006
C	3.9075620	2.2936044	1.3833505
C	1.9982387	0.4499250	2.8790634
C	-1.5510892	3.2503281	-1.3464241
C	-0.0303486	3.7886927	1.2699042
C	1.4935292	3.2760651	-1.3500115
H	1.1728720	-1.1171790	-2.7167062
H	1.5672168	0.5826286	-3.0031766
H	2.4841093	-0.6915610	-3.8275529
H	5.1589799	0.4003783	-2.7669069
H	5.4250766	0.6792198	-1.0423340
H	4.3159845	1.7269872	-1.9453650
H	4.6369172	-2.3562187	-0.5555000
H	4.4970370	-2.4389721	-2.3185201
H	3.1569696	-2.9816539	-1.2996002
H	4.3503333	-1.6527275	1.9358339
H	5.1716268	-0.3114248	2.7553382
H	5.4392994	-0.5861039	1.0303610
H	3.1059939	3.0340226	1.2934395
H	4.5933940	2.4361052	0.5414152
H	4.4610265	2.5155748	2.3052225
H	2.4816778	0.7353863	3.8214503
H	1.1587173	1.1332662	2.7139934
H	1.5878717	-0.5578866	3.0016045
H	-1.4919201	2.8175469	-2.3517823
H	-2.4691803	2.8838522	-0.8798751
H	-1.6361322	4.3387707	-1.4565904
H	-0.9122890	3.5523713	1.8767945
H	-0.0382923	4.8685691	1.0760624
H	0.8549984	3.5653508	1.8767949
H	2.4190243	2.9287999	-0.8833464
H	1.5575432	4.3656162	-1.4634877
H	1.4415799	2.8391882	-2.3539609

11^{+} : cation *t*Bu$_3$PSiMe$_3^{+}$

53

Energy = -1224.478807277

Si	-0.0006203	0.0035694	-2.0647752
C	1.2434435	1.2581781	-2.6997923
C	-1.7098874	0.4567721	-2.6956890
C	0.4614211	-1.7001505	-2.7043562
H	0.9653657	2.2926169	-2.4818648
H	1.2536894	1.1455935	-3.7923047
H	2.2606579	1.0842458	-2.3412268
H	-1.6190848	0.5277358	-3.7879486
H	-2.4663831	-0.3024500	-2.4806430
H	-2.0674057	1.4227388	-2.3313665
H	1.4972092	-1.9767943	-2.4908361
H	0.3547575	-1.6508504	-3.7964155
H	-0.1963949	-2.4945883	-2.3444417
P	0.0019539	-0.0012091	0.2887698
C	0.0292633	1.8051893	0.8866876
C	1.4344691	2.4171506	0.7145116
C	-0.9279740	2.6444301	0.0135842
C	-0.3832418	1.9417177	2.3648085
H	2.1704856	1.9836037	1.3920484
H	1.8011138	2.3397840	-0.3106281
H	1.3505126	3.4823161	0.9568498
H	-1.9628972	2.3093519	0.0583088
H	-0.8927074	3.6737826	0.3871863
H	-0.6074060	2.6651391	-1.0304818
H	-0.2769385	2.9980992	2.6367539
H	-1.4239894	1.6630556	2.5358788
H	0.2525502	1.3602202	3.0339059
C	1.5531311	-0.9318934	0.8805548
C	1.3747477	-2.4541700	0.7093501
C	2.7563226	-0.5287860	0.0014287
C	1.8857773	-0.6444335	2.3571655
H	0.6326499	-2.8723388	1.3898910
H	1.1193364	-2.7320773	-0.3147428
H	2.3385720	-2.9175252	0.9477329

H	2.9892387	0.5336324	0.0444619
H	3.6292076	-1.0783600	0.3709526
H	2.6076417	-0.8166829	-1.0418313
H	2.7452224	-1.2693667	2.6255774
H	2.1714266	0.3946944	2.5267051
H	1.0658121	-0.8994255	3.0299670
C	-1.5788420	-0.8781388	0.8828567
C	-1.8206845	-2.1356339	0.0206346
C	-1.5041809	-1.2865044	2.3665371
C	-2.8095320	0.0312081	0.6909529
H	-1.9904420	-1.8789744	-1.0275522
H	-1.0141236	-2.8643466	0.0793270
H	-2.7331544	-2.6155307	0.3916073
H	-1.3241066	-0.4372976	3.0273092
H	-2.4747989	-1.7190652	2.6349356
H	-0.7445720	-2.0467771	2.5534554
H	-3.6923757	-0.5703327	0.9341841
H	-2.8062473	0.8936549	1.3583012
H	-2.9179883	0.3752233	-0.3391952

12^+ : cyclic *c*-N(dipp)$SiMe_2FSiMe_2$N(dipp)BMe

84

Energy = -1948.828844917

B	0.0017182	-0.1460709	0.0276095
N	-1.2435712	0.6040365	-0.0812529
N	1.2385287	0.6132868	0.1204119
C	-0.0095015	-1.7175527	0.0259297
Si	-1.5294589	2.1856839	0.5337090
C	-2.4394680	-0.1019642	-0.5491160
Si	1.5134113	2.2113978	-0.4695623
C	2.4715074	-0.0580555	0.5430353
H	-0.2646809	-2.0950337	-0.9732184
H	-0.7809346	-2.1173904	0.6909855
H	0.9528376	-2.1515786	0.3074608
F	-0.0187485	3.0090908	0.0130612
C	-2.8611033	3.1080204	-0.3426007
C	-1.3996367	2.4842002	2.3521632
C	-3.2822257	-0.7518843	0.3742968
C	-2.7104181	-0.1199407	-1.9331683
C	1.4418337	2.5280391	-2.2880967
C	2.8101103	3.1475140	0.4447561
C	3.3098870	-0.6624916	-0.4133620
C	2.7872833	-0.0756360	1.9181449
H	-3.8336456	2.6551627	-0.1189260
H	-2.7224075	3.0879824	-1.4270210
H	-2.8886959	4.1490635	-0.0046153
H	-0.6155331	1.8805743	2.8143270
H	-2.3479398	2.2435066	2.8420597
H	-1.1889520	3.5431393	2.5386335
C	-4.3866133	-1.4554397	-0.1224977
C	-3.0687101	-0.6838778	1.8782716
C	-3.8226137	-0.8394916	-2.3800842
C	-1.8233663	0.6057970	-2.9312241
H	2.4082383	2.2999490	-2.7478331
H	1.2312874	3.5883909	-2.4672744
H	0.6779974	1.9266907	-2.7856163
H	3.7990174	2.7494780	0.1903997
H	2.7880202	4.2029617	0.1533244
H	2.6848707	3.0737823	1.5282715
C	4.4700845	-1.3046926	0.0396297
C	3.0111473	-0.6549796	-1.9036268
C	3.9544395	-0.7300909	2.3202906
C	1.8697514	0.5653006	2.9453414
C	-4.6527462	-1.5098496	-1.4856070
H	-5.0452090	-1.9672264	0.5732413
C	-3.0202834	-2.0742524	2.5355369
C	-4.1753984	0.1669068	2.5326900
H	-2.1044849	-0.1995106	2.0635942
H	-4.0448783	-0.8718761	-3.4421229
C	-2.6227917	1.3171681	-4.0339376
C	-0.7860955	-0.3515682	-3.5477358
H	-1.2768600	1.3733995	-2.3734561
C	4.7914208	-1.3434812	1.3906000
H	5.1268379	-1.7815376	-0.6822850
C	2.8234828	-2.0810050	-2.4528854
C	4.1205692	0.0745497	-2.6840540
H	2.0704643	-0.1185021	-2.0603878
H	4.2142410	-0.7597507	3.3734216
C	2.6187692	1.1837745	4.1339738
C	0.8168058	-0.4480765	3.4342855
H	1.3370582	1.3749160	2.4344042
H	-5.5116282	-2.0645892	-1.8515549
H	-2.2494115	-2.7071725	2.0863655
H	-2.8025448	-1.9742150	3.6038750
H	-3.9805894	-2.5898847	2.4355192
H	-4.2535485	1.1578066	2.0720572
H	-5.1481218	-0.3225546	2.4190655
H	-3.9811508	0.2952650	3.6028183
H	-3.3939385	1.9689941	-3.6110757
H	-1.9496256	1.9278343	-4.6445859
H	-3.1132752	0.6013656	-4.7009183
H	-0.1409086	-0.7904151	-2.7811987
H	-1.2886533	-1.1708827	-4.0720444
H	-0.1532241	0.1764560	-4.2696922
H	5.6948057	-1.8469923	1.7218106
H	2.0299536	-2.6116949	-1.9204069

H 2.5627245 -2.0428510 -3.5157707
H 3.7471131 -2.6602417 -2.3517229
H 4.2993897 1.0808546 -2.2898689
H 5.0647265 -0.4755681 -2.6178796
H 3.8517641 0.1577267 -3.7423141
H 3.4012853 1.8723513 3.7991426
H 1.9165541 1.7388582 4.7644815
H 3.0854524 0.4169702 4.7602815
H 0.2200894 -0.8381503 2.6039656
H 1.3062657 -1.2985081 3.9196700
H 0.1395184 0.0157347 4.1600574

1.$Et_3Si(tol)^+$: loose complex of **1** and $Et_3Si(tol)^+$

93

Energy = -2671.076486349

B 1.6488262 -0.1196034 -0.2660825
N 2.3258891 -0.0361588 -1.5220965
N 2.2515193 -0.1436837 1.0528240
F 0.2635838 -0.1554268 -0.3076442
Si 3.8109639 -1.0012957 -1.7201091
Si 1.5131275 0.7259223 -2.9091223
Si 2.8371238 1.4140852 1.6571456
Si 2.0658901 -1.5279111 2.1329193
C 5.2135743 -0.4974531 -0.5804811
C 3.3563700 -2.8048078 -1.4358000
C 4.4812257 -0.8981178 -3.4741669
C 2.7379792 1.7684351 -3.8919616
C 0.6969223 -0.5794687 -3.9891856
C 0.1835929 1.9294552 -2.3298793
C 3.3345409 2.5407475 0.2380092
C 1.4406429 2.2904682 2.5741512
C 4.2981684 1.1980699 2.8191203
C 1.4446203 -1.0383934 3.8425507
C 0.8169674 -2.7390640 1.4071365
C 3.7226979 -2.3857253 2.3763027
H 5.5754901 0.5121253 -0.8028489
H 4.9296557 -0.5402400 0.4726527
H 6.0523140 -1.1888878 -0.7340693
H 4.2298478 -3.4500711 -1.5906889
H 2.5790658 -3.1151727 -2.1448567
H 2.9803389 -2.9957116 -0.4269410
H 3.7351803 -1.0972893 -4.2498403
H 5.2516108 -1.6761921 -3.5585616
H 4.9592285 0.0613453 -3.6912521
H 3.4971281 2.2136421 -3.2385513
H 2.1908469 2.5907431 -4.3692050
H 3.2520990 1.2125058 -4.6797004
H -0.0459601 -1.1393020 -3.4077580
H 1.4209129 -1.3001279 -4.3851238
H 0.1829790 -0.1193646 -4.8423110
H -0.0894089 2.5725161 -3.1755496
H -0.7219433 1.4218326 -1.9928626
H 0.5286799 2.5842034 -1.5217677
H 3.6962244 3.4870835 0.6607118
H 4.1294895 2.1178744 -0.3804817
H 2.4910257 2.7767806 -0.4190071
H 1.7805034 3.2622448 2.9538223
H 1.0682867 1.7108775 3.4244951
H 0.5959686 2.4765704 1.8994386
H 4.0634144 0.5782513 3.6904740
H 4.5990998 2.1852874 3.1921280
H 5.1627009 0.7569627 2.3121831
H 1.3537990 -1.9455644 4.4540090
H 0.4577372 -0.5669712 3.8014802
H 2.1215445 -0.3578592 4.3690963
H 0.8828101 -2.8446382 0.3197089
H 0.9778441 -3.7316215 1.8456941
H -0.2045773 -2.4319345 1.6553939
H 4.4541440 -1.7138268 2.8385973
H 4.1518179 -2.7490850 1.4377552
H 3.5960525 -3.2478007 3.0435745
H -1.5380486 -2.0904081 -0.4278353
C -2.5600824 -1.9748241 -0.7951548
H -3.2315632 -2.4887067 -0.1002640
H -2.6308761 -2.4827967 -1.7622765
C -2.9263055 -0.4874204 -0.9440308
H -2.2109501 0.0053300 -1.6155390
H -3.9044180 -0.3761403 -1.4270104
Si -2.9086038 0.5200666 0.6323503
C -2.0484062 -0.2448292 2.1088035
C -2.3773443 2.3023228 0.4228082
H -2.2046077 -1.3295906 2.1077168
H -0.9822748 -0.0958565 1.8852019
C -2.3839571 0.3598213 3.4825519
H -2.4705554 2.8309182 1.3789127
H -1.2950750 2.1713627 0.2604596
C -2.9708173 3.1368370 -0.7236025
H -3.4227406 0.1648212 3.7699994
H -1.7501104 -0.0754654 4.2610235
H -2.2269821 1.4430727 3.4958698
H -3.9479990 3.5415900 -0.4484746
H -2.3199311 3.9862379 -0.9517692
H -3.0878047 2.5536307 -1.6423527
H -5.5813146 2.3728581 0.1658174
C -5.6279222 1.2947973 0.2607263
C -4.9671297 0.6530872 1.3446006
C -6.3199528 0.5475579 -0.6745800

C -5.1429647 -0.7502829 1.5095988
H -4.6590767 1.2527415 2.2025491
C -6.4268285 -0.8516530 -0.5481904
H -6.8051005 1.0447015 -1.5090882
C -5.8372907 -1.4829561 0.5619681
H -4.7158645 -1.2439803 2.3763095
C -7.1501407 -1.6544913 -1.5868753
H -5.9460123 -2.5560915 0.6848179
H -8.0043197 -1.1022584 -1.9879720
H -6.4740227 -1.8621752 -2.4270193
H -7.4879322 -2.6130577 -1.1865832

1 : neutral $[(Me_3Si)_2N]_2BF$
56
Energy = -1872.139436905
B 0.0965160 -0.0167014 -0.7486170
N -1.2449910 -0.2562425 -0.2917803
N 1.2768122 0.2443116 0.0288386
F 0.2760732 -0.0406020 -2.1216496
Si -2.0008297 0.7626781 0.9399802
Si -2.2127756 -1.3920076 -1.2598738
Si 1.6898492 -0.7324280 1.4434334
Si 2.4619661 1.3568448 -0.6924865
C -2.3230440 -0.1503756 2.5550258
C -0.9654336 2.2950560 1.2758423
C -3.6503322 1.4045802 0.2890045
C -3.4971181 -2.2194671 -0.1577310
C -3.0525995 -0.5295047 -2.7039829
C -1.1165821 -2.7712210 -1.9211493
C 1.5878669 0.2296601 3.0590630
C 0.6001973 -2.2603709 1.5483991
C 3.4502396 -1.3822621 1.2565068
C 1.5731791 2.7065100 -1.6564196
C 3.4196203 2.2273381 0.6765227
C 3.6450353 0.4576438 -1.8444734
H -2.8468795 0.5179402 3.2506220
H -2.9533665 -1.0331149 2.4045562
H -1.4000518 -0.4780950 3.0428685
H -0.8827251 2.9081695 0.3713195
H 0.0440185 2.0755906 1.6280644
H -1.4730847 2.8986141 2.0394015
H -3.5029378 2.0009232 -0.6188635
H -4.0999318 2.0581044 1.0478272
H -4.3771446 0.6185954 0.0621716
H -3.0147104 -2.7830998 0.6493489
H -4.0695222 -2.9338333 -0.7634367
H -4.2127140 -1.5260378 0.2939436
H -3.7630248 0.2337339 -2.3698807
H -3.6007985 -1.2538664 -3.3196906
H -2.3042019 -0.0416442 -3.3386733
H -0.6287078 -3.3198831 -1.1079784
H -1.7405086 -3.4861527 -2.4730038
H -0.3433123 -2.4043240 -2.6020269
H 2.2341051 1.1137924 3.0480935
H 0.5703010 0.5626269 3.2852634
H 1.9178382 -0.4135228 3.8850307
H -0.4655617 -2.0361731 1.6220259
H 0.8929206 -2.8381893 2.4345996
H 0.7510641 -2.9004914 0.6717530
H 3.6887071 -2.0129813 2.1227301
H 4.2116970 -0.5982447 1.2032319
H 3.5404772 -2.0029242 0.3575082
H 0.9998104 2.3125433 -2.5003838
H 2.3178647 3.4087456 -2.0527406
H 0.8934408 3.2760517 -1.0131903
H 2.7461402 2.8135327 1.3124216
H 3.9933893 1.5538209 1.3201596
H 4.1306775 2.9253420 0.2159256
H 4.2490659 -0.2864511 -1.3147763
H 4.3302511 1.1680297 -2.3242402
H 3.0848824 -0.0587230 -2.6322379

3 : neutral $[(Me_3Si)(C_6F_5)N]_2BF$
52
Energy = -2509.720119156
B 0.0024651 -0.0005825 1.9896216
N 0.3650680 1.2333585 1.3587680
N -0.3626607 -1.2340828 1.3592703
F 0.0048598 -0.0010181 3.3541808
Si 1.0492525 2.5972179 2.3220931
Si -1.0493350 -2.5964354 2.3227999
C 1.4694119 3.9629087 1.1082896
C -0.2479164 3.2071512 3.5300583
C 2.5846160 1.9750363 3.1943043
C 0.2465738 -3.2092179 3.5306384
C -2.5831994 -1.9707767 3.1951609
C -1.4727950 -3.9608578 1.1087103
H 2.2436450 3.6664152 0.3932115
H 1.8511017 4.8240560 1.6708614
H 0.5910130 4.3010580 0.5468610
H -0.5098452 2.4353437 4.2606093
H -1.1619763 3.5015128 3.0013446
H 0.1227461 4.0832885 4.0766262
H 3.0514514 2.7846992 3.7684571
H 2.3504532 1.1618130 3.8892359
H 3.3185896 1.6064828 2.4686777
H -0.1258197 -4.0846901 4.0770908
H 0.5101051 -2.4380356 4.2612671

H	1.1599996	-3.5053255	3.0018098
H	-3.3164232	-1.6006317	2.4695916
H	-3.0517636	-2.7793365	3.7694671
H	-2.3471096	-1.1580079	3.8899904
H	-0.5952908	-4.3008412	0.5469928
H	-2.2464262	-3.6622597	0.3938490
H	-1.8564194	-4.8213381	1.6709840
C	0.0254733	1.4653703	0.0054689
C	-1.3014845	1.7018298	-0.3759265
C	0.9860160	1.4836013	-1.0129210
C	-1.6636058	1.9154683	-1.7028866
C	0.6474945	1.6979049	-2.3443362
C	-0.6847031	1.9102372	-2.6931530
C	-0.0241657	-1.4672055	0.0058146
C	1.3016266	-1.7101760	-0.3754919
C	-0.9844879	-1.4797468	-1.0128745
C	1.6632546	-1.9228006	-1.7027491
C	-0.6462725	-1.6911288	-2.3448421
C	0.6848323	-1.9105401	-2.6934282
F	-2.2673382	1.7064728	0.5616593
F	2.2668231	-1.7235158	0.5627022
F	2.2791422	1.2571478	-0.7088750
F	-2.2773915	-1.2527761	-0.7082560
F	-2.9492375	2.1195460	-2.0343329
F	2.9479327	-2.1331324	-2.0340198
F	1.5944945	1.6825100	-3.2982870
F	-1.5923380	-1.6655493	-3.2995195
F	-1.0214953	2.1043130	-3.9773067
F	1.0210586	-2.1047343	-3.9777038

6 : neutral $[(Me_3Si)(Dipp)N]_2BF$
88
Energy = -1988.949010697

B	0.0002716	-1.2976938	-0.0064501
N	1.2446278	-0.7410268	-0.4327073
N	-1.2449167	-0.7459492	0.4238905
F	0.0008185	-2.6849501	-0.0124671
Si	2.3419025	-1.8276988	-1.3519885
C	1.8037219	0.5275349	-0.0286290
Si	-2.3386965	-1.8386856	1.3400248
C	-1.8056588	0.5250778	0.0297691
C	3.9367221	-0.8946809	-1.6945544
C	2.8183886	-3.3982484	-0.4284264
C	1.4653986	-2.3016865	-2.9460877
C	2.5320239	0.5950958	1.1830025
C	1.7519263	1.6427021	-0.8932371
C	-2.8163571	-3.4048319	0.4097423
C	-1.4574554	-2.3200654	2.9293838
C	-3.9326333	-0.9082589	1.6934353
C	-2.5364298	0.6009538	-1.1799509
C	-1.7547144	1.6331405	0.9033346
H	4.4920040	-0.7127918	-0.7685526
H	3.7896757	0.0728562	-2.1790891
H	4.5682786	-1.5120486	-2.3462961
H	3.6628390	-3.8718771	-0.9464632
H	1.9927435	-4.1135513	-0.3923229
H	3.1351581	-3.1939307	0.5991921
H	2.0816640	-2.9882167	-3.5396592
H	0.5178208	-2.8076244	-2.7276652
H	1.2449292	-1.4245311	-3.5634343
C	3.2378131	1.7664800	1.4788879
C	2.6204172	-0.5731989	2.1567272
C	2.4855406	2.7875206	-0.5606412
C	0.9181859	1.6407425	-2.1625918
H	-3.1392522	-3.1957058	-0.6149803
H	-3.6567890	-3.8834978	0.9297122
H	-1.9890907	-4.1177788	0.3653704
H	-2.0715678	-3.0096611	3.5216176
H	-0.5101140	-2.8242798	2.7060804
H	-1.2358734	-1.4454717	3.5500280
H	-4.4919991	-0.7210646	0.7710098
H	-3.7829901	0.0565828	2.1826228
H	-4.5614876	-1.5289049	2.3446645
C	-3.2457383	1.7730184	-1.4645711
C	-2.6216843	-0.5582359	-2.1647943
C	-2.4915498	2.7790236	0.5816607
C	-0.9186247	1.6223690	2.1710620
C	3.2374068	2.8508902	0.6081947
H	3.8065081	1.8221850	2.4037833
C	2.0898831	-0.2069912	3.5546344
C	4.0671656	-1.0888651	2.2786005
H	2.0027798	-1.3862382	1.7641924
H	2.4630327	3.6431419	-1.2304330
C	1.7719634	1.6032392	-3.4429589
C	-0.0166710	2.8600827	-2.2082112
H	0.3018963	0.7384579	-2.1327781
C	-3.2461134	2.8501981	-0.5849583
H	-3.8167156	1.8349018	-2.3876677
C	-2.0935572	-0.1762630	-3.5594108
C	-4.0667106	-1.0776240	-2.2908215
H	-2.0008302	-1.3728602	-1.7807920
H	-2.4692488	3.6292938	1.2582235
C	-1.7698375	1.5706117	3.4526512
C	0.0138300	2.8432122	2.2258566
H	-0.3002840	0.7218531	2.1320016
H	3.8043344	3.7464682	0.8476684
H	2.6778909	0.6044970	3.9973492
H	1.0461297	0.1127245	3.5171873

H	2.1577756	-1.0752387	4.2195799
H	4.1043444	-1.9655293	2.9351644
H	4.4787500	-1.3710851	1.3058642
H	4.7162572	-0.3169004	2.7069266
H	2.4474992	2.4655970	-3.4806387
H	2.3761149	0.6956135	-3.5101120
H	1.1232585	1.6434293	-4.3257955
H	-0.6130340	2.9329816	-1.2968655
H	0.5558781	3.7873808	-2.3252220
H	-0.6998223	2.7781406	-3.0599973
H	-3.8159040	3.7462520	-0.8156927
H	-1.0508428	0.1465541	-3.5194772
H	-2.1590744	-1.0379604	-4.2330474
H	-2.6846115	0.6377138	-3.9934358
H	-4.1012125	-1.9480323	-2.9557995
H	-4.4767929	-1.3706851	-1.3206857
H	-4.7187450	-0.3038606	-2.7113675
H	-2.4479362	2.4305031	3.4993725
H	-2.3710945	0.6605062	3.5125641
H	-1.1196530	1.6047198	4.3346437
H	-0.5603920	3.7682058	2.3523653
H	0.6988003	2.7550832	3.0755736
H	0.6082801	2.9256404	1.3140450

$7F^+$: cation $(Me_3Si)_2NBN(SiMe_3)(SiMe_2F)^+$
52
Energy = -1832.020340724

B	0.1365892	0.2930022	-0.6463278
N	1.4442679	0.3168234	-0.9091945
N	-1.1816112	0.2628094	-0.3913788
F	-1.2260947	1.5561073	-2.6960784
Si	2.0471819	-0.8385334	-2.1957991
Si	2.4644488	1.5528617	-0.0297370
Si	-1.7657094	-0.7752477	1.0027220
Si	-2.2402297	1.2355858	-1.4708209
C	3.6833422	-1.5257566	-1.6209131
C	0.7399238	-2.1739621	-2.3038965
C	2.1743822	0.1179916	-3.7919454
C	3.5534997	2.3920267	-1.2903103
C	1.2408492	2.7530560	0.7233775
C	3.4416138	0.6572790	1.2838867
C	-0.2418471	-1.0998061	2.0355690
C	-3.0455493	0.2265248	1.9181234
C	-2.4673196	-2.3500312	0.2909820
C	-2.7123800	2.8306386	-0.6625928
C	-3.6436290	0.1980070	-2.0806162
H	4.4539467	-0.7536198	-1.5262605
H	3.5900675	-2.0446998	-0.6612064
H	4.0384721	-2.2522950	-2.3620257
H	1.0342466	-2.9102695	-3.0611840
H	-0.2376872	-1.7777695	-2.6027035
H	0.6247817	-2.7080324	-1.3537870
H	1.2148061	0.5822182	-4.0451956
H	2.4460525	-0.5640359	-4.6068336
H	2.9345833	0.9034500	-3.7464489
H	4.2615106	1.7018093	-1.7605795
H	4.1404296	3.1703364	-0.7873460
H	2.9620650	2.8730702	-2.0763790
H	0.5404141	2.2675602	1.4131744
H	0.6639354	3.2756133	-0.0479556
H	1.7839491	3.5127513	1.2978985
H	4.0104659	1.3880887	1.8717061
H	2.7854404	0.1130004	1.9713762
H	4.1549972	-0.0536890	0.8567392
H	0.5387499	-1.6218802	1.4696257
H	0.1819530	-0.1738404	2.4399134
H	-0.5073195	-1.7389231	2.8858160
H	-2.6369705	1.1762167	2.2792872
H	-3.3888688	-0.3440766	2.7894495
H	-3.9272757	0.4383339	1.3029782
H	-3.3423511	-2.1670818	-0.3400456
H	-2.7812474	-3.0072497	1.1111370
H	-1.7228037	-2.8899332	-0.3034526
H	-3.2573605	3.4577648	-1.3775205
H	-1.8319070	3.3849081	-0.3223819
H	-3.3650768	2.6613678	0.1997481
H	-3.2898867	-0.7251553	-2.5504383
H	-4.3286692	-0.0656475	-1.2670389
H	-4.2178808	0.7640953	-2.8230238

7^+.$MeSiEt_3$: loose complex of **7^+** and Et_3SiMe
78
Energy = -2399.322864775

B	3.1053617	0.1837768	-0.8179125
N	3.5066760	-0.9715594	-0.0979564
N	2.1570179	1.2197757	-0.3631365
F	3.6311652	0.4140368	-2.0606300
Si	2.2314995	-2.0269122	0.5506793
Si	5.1931310	-1.5394003	-0.3308511
Si	2.6416080	2.1993194	1.0757605
Si	0.9543211	1.7468046	-1.4498901
C	1.1040585	-1.1720819	1.7896105
C	1.2062660	-2.5972117	-0.9224150
C	2.9257910	-3.5467072	1.3967662
C	5.9008199	-2.1174942	1.3119222
C	5.2449808	-2.9090676	-1.6126883

C	6.2603852	-0.0950730	-0.8915656
C	3.8122996	1.2458545	2.1765269
C	3.5384273	3.7228038	0.4509002
C	1.1138698	2.6852198	2.0528742
C	0.5982291	3.5625082	-1.5698779
C	0.8564514	0.8495894	-3.0682806
C	-0.9796407	1.0795208	-0.5046726
H	1.6059477	-0.9899965	2.7452754
H	0.7210504	-0.2155887	1.4263236
H	0.2468977	-1.8274575	1.9898272
H	0.4545997	-3.3368436	-0.6234229
H	1.8501954	-3.0602825	-1.6792355
H	0.6774442	-1.7708528	-1.4136969
H	3.5969772	-4.1353821	0.7643927
H	2.0721063	-4.1881220	1.6524595
H	3.4469629	-3.3054492	2.3278897
H	5.6543210	-1.4227771	2.1227565
H	6.9939927	-2.1429384	1.2197333
H	5.5727326	-3.1168385	1.6063398
H	4.8661846	-2.5480285	-2.5759667
H	4.6428752	-3.7753793	-1.3166924
H	6.2741156	-3.2572028	-1.7639621
H	7.3124797	-0.3937299	-0.8034871
H	6.0779365	0.1967147	-1.9286234
H	6.1243783	0.7908650	-0.2589861
H	4.0281116	1.8788549	3.0471876
H	3.4005719	0.3010067	2.5353407
H	4.7632677	1.0347704	1.6790819
H	3.9268439	4.2976307	1.3006223
H	2.9001177	4.3911311	-0.1351992
H	4.3918212	3.4376762	-0.1759486
H	0.4004192	3.2950778	1.4869776
H	1.4333174	3.2974945	2.9052735
H	0.5892659	1.8147158	2.4601075
H	-0.3218007	3.7277855	-2.1416428
H	1.4197227	4.0417916	-2.1154073
H	0.4984108	4.0587120	-0.6016152
H	0.9460157	-0.2353448	-2.9657160
H	-0.0908551	1.0765530	-3.5692395
H	1.6726215	1.1946281	-3.7122041
H	-0.6346448	1.8123270	0.2285506
H	-0.4956310	0.1026468	-0.4728249
H	-1.2598069	1.4541830	-1.4931748
H	-1.5894227	-2.0706712	-0.4111813
C	-2.6732149	-2.1242122	-0.2649866
H	-2.8556498	-2.3074276	0.7986124
H	-3.0339485	-2.9947094	-0.8217994
C	-3.3712654	-0.8358304	-0.7397653
H	-3.2316900	-0.7054586	-1.8214577
H	-4.4565602	-0.9227961	-0.5873305
Si	-2.8204958	0.7221363	0.1492412
C	-2.7185128	0.5010388	2.0115069
C	-3.8008412	2.2414057	-0.3574423
H	-3.6133930	-0.0612309	2.3139899
H	-1.8676958	-0.1504298	2.2503948
C	-2.6362809	1.8109509	2.8189342
H	-4.8108853	2.1272307	0.0611088
H	-3.3734212	3.1241715	0.1370948
C	-3.8956055	2.4790839	-1.8764917
H	-3.5409211	2.4120091	2.6865256
H	-2.5248509	1.6040739	3.8880576
H	-1.7842059	2.4285146	2.5143852
H	-4.4920055	3.3695686	-2.0978738
H	-2.9076024	2.6378267	-2.3277954
H	-4.3591778	1.6297944	-2.3879808

7$^+$: borinium cation $[(Me_3Si)_2N]_2B^+$

55

Energy = -1772.007831636

B	-0.0075885	0.0006307	-0.0051275
N	0.1099686	1.3339076	0.0034120
N	-0.1215202	-1.3329557	-0.0144779
Si	1.4121739	2.0597643	1.0623208
Si	-1.0419313	2.2857946	-1.0509160
Si	-1.4151449	-2.0759957	1.0427483
Si	1.0407813	-2.2691805	-1.0717255
C	1.9200083	0.7000767	2.2441492
C	2.8410411	2.5495056	-0.0354631
C	0.6758794	3.5189044	1.9616762
C	-2.3366245	3.0523143	0.0547617
C	-0.0482450	3.5650206	-1.9758230
C	-1.8175669	1.0368334	-2.2093974
C	-1.9734371	-0.7096459	2.1938578
C	-2.8192679	-2.6239970	-0.0592962
C	-0.6462843	-3.4971663	1.9752482
C	0.0714822	-3.5880120	-1.9661851
C	1.7680649	-1.0179373	-2.2587452
C	2.3682521	-2.9861344	0.0284214
H	1.0926962	0.3764776	2.8845645
H	2.3168247	-0.1785024	1.7230333
H	2.7167868	1.0734595	2.8983492
H	3.6563794	2.9355205	0.5887099
H	2.5704352	3.3311170	-0.7510816
H	3.2303498	1.6924494	-0.5958823
H	0.3792418	4.3233607	1.2805307
H	1.4308409	3.9294711	2.6432713
H	-0.1947020	3.2297059	2.5595189
H	-2.8693285	2.2922195	0.6365877

H	-3.0756841	3.5725084	-0.5669652
H	-1.9146171	3.7820168	0.7514893
H	0.7436307	3.1058838	-2.5768817
H	0.4057953	4.3037357	-1.3071328
H	-0.7160040	4.1067208	-2.6567614
H	-2.5308699	1.5516482	-2.8641509
H	-1.0762821	0.5481349	-2.8504511
H	-2.3754130	0.2607346	-1.6731339
H	-1.1605931	-0.3429804	2.8295514
H	-2.3997486	0.1425324	1.6524573
H	-2.7588265	-1.0968461	2.8537803
H	-3.6363339	-3.0053656	0.5655112
H	-2.5255127	-3.4218959	-0.7470070
H	-3.2151886	-1.7910813	-0.6505703
H	-0.3188727	-4.3039117	1.3110374
H	-1.3953510	-3.9184303	2.6567828
H	0.2095563	-3.1719108	2.5759726
H	0.7462750	-4.1195231	-2.6482449
H	-0.7403056	-3.1597965	-2.5633875
H	-0.3527515	-4.3304548	-1.2822631
H	2.3009777	-0.2118950	-1.7416933
H	2.4954378	-1.5208067	-2.9072550
H	1.0067110	-0.5684105	-2.9049275
H	1.9708645	-3.7049787	0.7506601
H	2.8985514	-2.2031032	0.5813265
H	3.1049892	-3.5095310	-0.5933517

9^{+} : cation $PMe_3SiMe_3^+$

26

Energy = -870.4651140423

Si	0.0000056	0.0002378	-1.2164276
C	-0.0000305	1.8071229	-1.6983576
C	-1.5648746	-0.9034650	-1.6974975
C	1.5649034	-0.9033878	-1.6976091
H	-0.8899145	2.3284350	-1.3299157
H	0.0002811	1.8824655	-2.7926774
H	0.8895579	2.3285487	-1.3293694
H	-1.6309220	-0.9411208	-2.7917687
H	-1.5708599	-1.9345909	-1.3286946
H	-2.4609948	-0.3938635	-1.3279783
H	2.4610972	-0.3933310	-1.3288860
H	1.6304456	-0.9417976	-2.7918846
H	1.5713241	-1.9342430	-1.3280561
P	-0.0000007	0.0002673	1.0885643
C	-1.4657788	0.8464423	1.7445071
H	-2.3687925	0.3376622	1.3973679
H	-1.4385629	0.8323427	2.8377573
H	-1.4782102	1.8820767	1.3952250
C	-0.0002435	-1.6925742	1.7439496
H	-0.0017007	-1.6621483	2.8372252
H	-0.8909522	-2.2210777	1.3945196
H	0.8917658	-2.2204337	1.3968147
C	1.4659855	0.8459940	1.7446747
H	2.3689417	0.3387578	1.3951896
H	1.4773081	1.8825110	1.3978753
H	1.4402222	0.8291703	2.8379113

A^{+} : cation $(Me_3Si)_2NBFN(SiMe_3)SiMe_2^+$

52

Energy = -1831.986180883

B	0.0478921	0.6739449	0.4650931
N	-1.1583445	0.0456696	0.0900617
N	1.4126166	0.1082776	0.3487057
F	0.0166095	1.9349515	0.9817450
Si	-1.2959085	-1.3575731	-0.9923662
Si	-2.6603995	0.9637918	0.5389831
Si	2.5761558	1.2215596	-0.5065993
Si	1.6573534	-1.4384852	0.9239115
C	-1.9497700	-0.7859878	-2.6509056
C	0.3854932	-2.1512042	-1.3927621
C	-2.3362865	-2.7253182	-0.2355610
C	-4.1960479	-0.0644522	0.2103214
C	-2.6267524	1.3099079	2.3818087
C	-2.7334436	2.5231334	-0.4964512
C	3.9400392	0.2236228	-1.3110417
C	1.5609184	2.0618610	-1.8373476
C	3.2723778	2.4140405	0.7482350
C	0.4694009	-2.1449217	2.1430133
C	3.2846936	-2.2871693	0.7704379
H	-2.0723732	-1.6417030	-3.3256275
H	-2.9201591	-0.2868418	-2.5686549
H	-1.2513001	-0.0830761	-3.1197651
H	0.1714933	-2.9703876	-2.0947412
H	0.8861866	-2.7152810	-0.5693689
H	1.1154258	-1.4913879	-1.8660768
H	-3.3970265	-2.6192551	-0.4768877
H	-2.2444774	-2.7596643	0.8551105
H	-2.0051242	-3.6937497	-0.6294929
H	-5.0512597	0.5857940	0.4382619
H	-4.2639834	-0.9335396	0.8715950
H	-4.3184369	-0.4016861	-0.8228752
H	-3.5721439	1.7813785	2.6779139
H	-1.8116335	1.9745939	2.6796047
H	-2.5368393	0.3756101	2.9496997
H	-2.7480273	2.2815416	-1.5658506
H	-3.6491854	3.0825635	-0.2693791
H	-1.8801331	3.1814766	-0.3068562
H	4.6493973	-0.2004537	-0.5944942

H	3.5632770	-0.5794095	-1.9543388
H	4.5037228	0.9121043	-1.9537049
H	1.1381033	1.3309599	-2.5368856
H	2.2083168	2.7321294	-2.4155284
H	0.7422335	2.6655242	-1.4325170
H	2.4807366	3.0057454	1.2180630
H	3.9743439	3.1035187	0.2635795
H	3.8169990	1.8820050	1.5371699
H	0.9805550	-2.1654178	3.1146828
H	0.2194807	-3.1802026	1.8884219
H	-0.4470023	-1.5600071	2.2351875
H	3.6434539	-2.3333737	-0.2595239
H	4.0307739	-1.7516503	1.3710706
H	3.1941060	-3.3028050	1.1684949

$B(C_6F_5)_4^-$: counter-anion
45
Energy = -2937.881433347

C	-2.3072731	-2.3330669	3.0947830
C	-0.9529064	-2.5509830	2.8659657
C	-2.9717616	-1.3889095	2.3246494
C	-0.3073252	-1.8236629	1.8741466
C	-2.2803964	-0.6845438	1.3386317
C	-0.9283230	-0.8775023	1.0522909
F	-2.9605894	-3.0219719	4.0522771
C	-0.8777813	0.9288981	-1.0524194
C	-1.8246929	0.3077917	-1.8732966
C	-0.6841469	2.2806932	-1.3398007
C	-2.5530266	0.9533551	-2.8643758
C	-1.3883609	2.9716214	-2.3262016
C	-2.3346121	2.3074733	-3.0940536
C	0.9289815	0.8782940	1.0521471
C	2.2806254	0.6840989	1.3396172
C	0.3083047	1.8262167	1.8721298
C	2.9716343	1.3877478	2.3263733
C	0.9534181	2.5523210	2.8651236
C	2.3070478	2.3321524	3.0961177
C	0.8780821	-0.9285111	-1.0528282
C	1.8260644	-0.3083980	-1.8731691
C	0.6837299	-2.2802582	-1.3397655
C	2.5529715	-0.9543512	-2.8650458
C	1.3876371	-2.9719593	-2.3258720
C	2.3325994	-2.3080455	-3.0955277
F	-0.2853493	-3.4625814	3.6083497
F	-4.2851034	-1.1535626	2.5451431
F	-3.0228859	0.2322180	0.6677969
F	1.0181744	-2.0769008	1.7210686
F	-1.0162010	2.0826259	1.7156380
F	0.2858670	3.4639835	3.6074321
F	2.9598095	3.0197860	4.0549043
F	4.2847294	1.1518560	2.5476990
F	3.0225886	-0.2334674	0.6693535
F	0.2326890	3.0232390	-0.6691856
F	-1.1515973	4.2843631	-2.5486799
F	-3.0253361	2.9610034	-4.0500961
F	-3.4648558	0.2856930	-3.6063733
F	-2.0778590	-1.0176455	-1.7196120
F	2.0816929	1.0164320	-1.7183833
F	3.4665070	-0.2877832	-3.6059230
F	3.0200715	-2.9613119	-4.0540730
F	1.1512215	-4.2850311	-2.5468190
F	-0.2345380	-3.0217588	-0.6698781
B	0.0004749	0.0003428	-0.0002587

B^+ : $Me_3PB[N(SiMe_3)_2]^+$, adduct PMe_3
68
Energy = -2233.240125057

B	0.0829285	-0.3323150	-0.0210334
N	1.2944694	0.3744569	-0.1002087
N	-1.2736120	0.1521865	0.0891347
Si	1.4124743	2.0975526	-0.6501733
Si	2.8741615	-0.4219287	0.2565284
Si	-2.4943722	0.1691635	-1.2093445
Si	-1.7159912	0.7216273	1.7300736
C	2.5367871	2.0999211	-2.1575038
C	-0.1880338	2.8506136	-1.2493621
C	2.0435758	3.1730988	0.7586127
C	4.2809512	0.7749348	0.5917169
C	2.7278407	-1.3458113	1.8913035
C	3.3624263	-1.4362314	-1.2510039
C	-3.6974013	-1.2811304	-1.1214690
C	-3.5673317	1.7055281	-1.0927510
C	-1.6183324	0.1288951	-2.8869154
C	-3.3801702	0.0112689	2.2373752
C	-0.4080950	0.1243065	2.9402125
C	-1.8056954	2.5897673	1.8946088
H	2.6441309	3.1322036	-2.5138057
H	3.5410323	1.7027003	-1.9943336
H	2.0716666	1.5205884	-2.9645111
H	-0.6116743	2.3261636	-2.1070837
H	0.0684847	3.8657835	-1.5822783
H	-0.9498591	2.9380998	-0.4767197
H	1.6802481	2.8190378	1.7300686
H	1.6663430	4.1931663	0.6171030
H	3.1334742	3.2279875	0.8078827
H	5.1571229	0.1340073	0.7667921
H	4.1196983	1.3525685	1.5060866

H	4.5392317	1.4596355	-0.2179469
H	1.7491908	-1.7778680	2.1056739
H	2.9376966	-0.6299445	2.6946481
H	3.4766834	-2.1431945	1.9587189
H	2.5385345	-1.9843616	-1.7171633
H	3.7557504	-0.7524445	-2.0124266
H	4.1568613	-2.1526501	-1.0103799
H	-4.4547711	-1.1250422	-1.9011221
H	-3.2523840	-2.2639776	-1.3032657
H	-4.2250422	-1.3218757	-0.1626354
H	-4.3017357	1.6533341	-1.9071707
H	-3.0037066	2.6338592	-1.2135786
H	-4.1309085	1.7620875	-0.1559680
H	-2.0294667	-0.6617878	-3.5250848
H	-0.5366214	-0.0309900	-2.8121808
H	-1.7695153	1.0755542	-3.4171248
H	-3.6035541	0.3671454	3.2512371
H	-3.3842533	-1.0828751	2.2659644
H	-4.2018290	0.3382197	1.5925468
H	0.5714305	0.5727028	2.7486396
H	-0.7146276	0.4237305	3.9502088
H	-0.2873765	-0.9640269	2.9517248
H	-2.5203308	3.0520120	1.2071319
H	-0.8324739	3.0701492	1.7553431
H	-2.1387034	2.8155555	2.9163171
P	0.0070676	-2.4133191	-0.0939621
C	-1.3856272	-2.9573948	0.9383895
H	-2.2568629	-2.3307398	0.7585850
H	-1.6208886	-3.9987927	0.7020946
H	-1.1002787	-2.8884310	1.9911532
C	-0.4149672	-2.8475669	-1.8138109
H	0.4415844	-2.6389926	-2.4599326
H	-0.6476048	-3.9151622	-1.8571480
H	-1.2711334	-2.2777275	-2.1697348
C	1.2844506	-3.6603590	0.3153828
H	1.4057014	-3.7340497	1.3959894
H	0.9126235	-4.6160739	-0.0674821
H	2.2457510	-3.4408077	-0.1455198

C : neutral $Me_3SiN{=}BN(SiMe_3)_2$
42
Energy = -1362.746857598

B	-0.3972106	-0.0125324	0.0005847
N	0.9951424	-0.0083957	0.0030592
N	-1.6684500	-0.0149932	-0.0006960
Si	1.8139436	-1.5891003	-0.0345843
Si	1.7996329	1.5799996	0.0447231
Si	-3.3679677	0.0015567	-0.0006251
C	0.5608959	-2.8771191	0.5043685
C	2.3960796	-1.9546333	-1.7817770
C	3.2716580	-1.5660861	1.1483646
C	2.3603768	1.9548497	1.7969327
C	3.2700159	1.5667229	-1.1226839
C	0.5415446	2.8556573	-0.5114525
C	-4.0195029	-0.7860446	1.5834412
C	-3.9741135	1.7833710	-0.1069962
C	-4.0197995	-0.9684612	-1.4798050
H	0.1963905	-2.6785618	1.5188085
H	-0.3073095	-2.8958537	-0.1644004
H	1.0174145	-3.8746178	0.4954647
H	2.8505058	-2.9519189	-1.8354630
H	3.1403199	-1.2277478	-2.1241067
H	1.5527761	-1.9299878	-2.4821099
H	4.0219015	-0.8154139	0.8763004
H	3.7679490	-2.5446155	1.1372643
H	2.9451338	-1.3654593	2.1749457
H	1.5099110	1.9266248	2.4884229
H	2.8071741	2.9555198	1.8519807
H	3.1060913	1.2343949	2.1495538
H	2.9560196	1.3604860	-2.1520491
H	4.0240228	0.8235096	-0.8407377
H	3.7574629	2.5496481	-1.1091509
H	0.9877521	3.8577268	-0.4936831
H	0.1960670	2.6549849	-1.5320956
H	-0.3380498	2.8644578	0.1423853
H	-3.6755740	-1.8240698	1.6705293
H	-3.6619888	-0.2406519	2.4653873
H	-5.1167566	-0.7903445	1.6093672
H	-5.0703702	1.8354300	-0.1119374
H	-3.6080726	2.2642660	-1.0223031
H	-3.6119638	2.3696472	0.7464029
H	-3.6615177	-0.5322178	-2.4201774
H	-5.1170887	-0.9739223	-1.5049201
H	-3.6783456	-2.0102918	-1.4426919

Da$^+$: borinium cation $[(Me_3Si)(Dipp)N]_2B^+$
87
Energy = -1888.799600390

B	0.0018534	-0.0160592	-0.6100554
N	-1.2499449	-0.4941981	-0.6230262
N	1.2518754	0.4655461	-0.6464095
Si	-1.9198695	-1.5702839	-1.9634552
C	-2.1290804	-0.1009242	0.5045105
Si	1.9215944	1.4816446	-2.0328994
C	2.1313081	0.1252786	0.4979386
C	-2.1404099	-0.4856067	-3.4691302
C	-0.6380091	-2.8875609	-2.2835390

C	-3.5331470	-2.2381092	-1.3227141
C	-2.8835014	1.0792763	0.4196750
C	-2.1714457	-0.9575743	1.6206730
C	2.1304055	0.3358829	-3.4945225
C	0.6473757	2.7935229	-2.4012855
C	3.5412419	2.1649844	-1.4256860
C	2.8946734	-1.0516824	0.4629891
C	2.1606181	1.0249529	1.5801094
H	-2.9038172	0.2839201	-3.3228594
H	-2.4529357	-1.1071419	-4.3171079
H	-1.2059475	0.0099408	-3.7561560
H	0.3268497	-2.4626156	-2.5831993
H	-0.9783646	-3.5262865	-3.1072943
H	-0.4759409	-3.5261227	-1.4101574
H	-4.2205575	-1.4379172	-1.0314309
H	-4.0103361	-2.8197309	-2.1208185
H	-3.3938014	-2.9002866	-0.4634567
C	-3.7029091	1.3983669	1.5094415
C	-2.8447178	2.0095614	-0.7792680
C	-3.0039882	-0.5869130	2.6818219
C	-1.3264420	-2.2177334	1.7279183
H	2.4361282	0.9214888	-4.3700840
H	1.1937240	-0.1710407	-3.7532117
H	2.8951108	-0.4271047	-3.3224800
H	0.9921976	3.4003084	-3.2470401
H	-0.3195171	2.3633629	-2.6863871
H	0.4882724	3.4640803	-1.5516433
H	3.4101488	2.8547295	-0.5871264
H	4.0141454	2.7191934	-2.2455501
H	4.2285382	1.3726675	-1.1135008
C	3.7137544	-1.3195848	1.5666258
C	2.8632649	-2.0313135	-0.6962856
C	2.9919071	0.7033799	2.6582365
C	1.3046729	2.2811105	1.6361452
C	-3.7629744	0.5787175	2.6305819
H	-4.2974859	2.3064921	1.4747004
C	-2.3713893	3.4173455	-0.3759214
C	-4.2179493	2.0758286	-1.4724493
H	-2.1254824	1.6066578	-1.4983642
H	-3.0540166	-1.2208625	3.5615991
C	-2.1302035	-3.4266946	2.2339450
C	-0.1079207	-1.9667201	2.6381088
H	-0.9537641	-2.4625706	0.7277096
C	3.7623229	-0.4558082	2.6549237
H	4.3159886	-2.2233308	1.5701289
C	2.3908968	-3.4216947	-0.2352378
C	4.2387624	-2.1241157	-1.3815827
H	2.1456481	-1.6608540	-1.4342418
H	3.0315489	1.3710806	3.5133695

C	2.1072797	3.5218611	2.0615057
C	0.1055487	2.0693206	2.5812653
H	0.9113183	2.4691381	0.6317910
H	-4.4014913	0.8475541	3.4667127
H	-1.3942848	3.3809969	0.1148399
H	-2.2927800	4.0583293	-1.2597206
H	-3.0785439	3.8823352	0.3182509
H	-4.9681103	2.5108436	-0.8040111
H	-4.1597595	2.7006795	-2.3698584
H	-4.5672924	1.0805428	-1.7649093
H	-3.0251835	-3.5994992	1.6283952
H	-1.5075142	-4.3262509	2.1942412
H	-2.4463422	-3.2884672	3.2726578
H	0.5163793	-1.1494314	2.2661657
H	-0.4398596	-1.7037908	3.6480465
H	0.5118506	-2.8667382	2.7030273
H	4.4001004	-0.6858644	3.5031038
H	1.4118131	-3.3663774	0.2496954
H	2.3171240	-4.0998206	-1.0913320
H	3.0962084	-3.8550594	0.4809614
H	4.9886871	-2.5253405	-0.6920959
H	4.1852376	-2.7895927	-2.2495835
H	4.5851974	-1.1420363	-1.7186700
H	2.9838294	3.6747872	1.4245943
H	1.4734546	4.4120951	1.9956212
H	2.4527598	3.4364953	3.0965758
H	-0.5233812	2.9649796	2.6057095
H	-0.5152252	1.2257206	2.2668317
H	0.4575353	1.8682857	3.5985960

D^{+} : $[(Me_3Si)(Dipp)N]B[N(Dipp)(SiMe_2F)]^{+}$

84

Energy = -1948.811488584

B	0.0835807	-0.5536203	-0.3335299
N	1.2247080	-0.2555187	-0.9797850
N	-1.0341309	-0.8893044	0.3175359
F	2.0136925	-2.4168582	-2.3679457
Si	1.6622762	-0.8592857	-2.6290483
C	2.2268780	0.5171889	-0.1970769
Si	-1.5580242	-2.6350495	0.6334895
C	-1.8539187	0.2362790	0.8275108
C	3.1736149	0.0372876	-3.1897519
C	0.1723744	-0.7866005	-3.7208783
C	3.0984864	-0.1540625	0.6742779
C	2.2545090	1.9094389	-0.3984589
C	-1.3645763	-3.4958910	-1.0126799
C	-0.4176403	-3.3785746	1.9074102
C	-3.3158146	-2.5153052	1.2300866

C	-2.9206743	0.7062220	0.0434093
C	-1.5254216	0.7779820	2.0819338
H	3.5304124	-0.4141539	-4.1223607
H	3.9782511	-0.0149122	-2.4503183
H	2.9557447	1.0914896	-3.3879187
H	0.3857236	-1.3162097	-4.6561942
H	-0.7019108	-1.2615540	-3.2629761
H	-0.0866647	0.2469477	-3.9703269
C	4.0333911	0.6284938	1.3646528
C	3.0931408	-1.6609578	0.8698962
C	3.2052239	2.6392910	0.3220777
C	1.2785159	2.6223847	-1.3218177
H	-2.0109353	-3.0616867	-1.7830858
H	-1.6350080	-4.5534886	-0.9100449
H	-0.3274257	-3.4630715	-1.3685685
H	-0.7302211	-4.4134655	2.0945610
H	0.6182673	-3.4100720	1.5553307
H	-0.4439779	-2.8462375	2.8623830
H	-3.6666006	-3.5243191	1.4786727
H	-3.9864897	-2.1050943	0.4698401
H	-3.3989976	-1.8985500	2.1306943
C	-3.6800735	1.7604162	0.5603155
C	-3.2058107	0.1648668	-1.3464245
C	-2.3218611	1.8299334	2.5514712
C	-0.3656851	0.2830111	2.9299871
C	4.0867255	2.0071077	1.1951184
H	4.7279396	0.1444808	2.0442923
C	3.0379869	-2.0434016	2.3590005
C	4.3239378	-2.2969858	0.1948299
H	2.2028009	-2.0701504	0.3809840
H	3.2539096	3.7161671	0.1976285
C	1.9445703	3.7311678	-2.1511577
C	0.0960416	3.1887533	-0.5115246
H	0.8744325	1.8842247	-2.0243893
C	-3.3866599	2.3176680	1.8022808
H	-4.5084193	2.1529809	-0.0210011
C	-2.5922350	1.1091042	-2.3986438
C	-4.7033185	-0.0442838	-1.6174608
H	-2.7110079	-0.8082121	-1.4385757
H	-2.0968522	2.2713323	3.5178973
C	-0.8677051	-0.2962989	4.2651599
C	0.6692485	1.3966342	3.1701250
H	0.1366540	-0.5222038	2.3856814
H	4.8178348	2.5927705	1.7442335
H	3.9402595	-1.7159287	2.8845092
H	2.1746426	-1.5923399	2.8571292
H	2.9692030	-3.1307862	2.4640138
H	4.2982076	-3.3850853	0.3129136
H	4.3564256	-2.0700650	-0.8744302
H	5.2455350	-1.9222043	0.6526291
H	2.2676420	4.5644616	-1.5193598
H	2.8188566	3.3583024	-2.6939969
H	1.2283322	4.1267342	-2.8781838
H	-0.4331413	2.4049614	0.0393558
H	0.4559151	3.9237316	0.2161413
H	-0.6206130	3.6821661	-1.1757788
H	-3.9885768	3.1368539	2.1841130
H	-1.5183857	1.2439428	-2.2315805
H	-2.7407492	0.7126787	-3.4080510
H	-3.0627326	2.0959818	-2.3402587
H	-4.8391838	-0.5207851	-2.5934992
H	-5.2429248	0.9078271	-1.6356481
H	-5.1646564	-0.6803651	-0.8557212
H	-1.3414934	0.4818209	4.8724374
H	-1.6030738	-1.0915827	4.1057693
H	-0.0295716	-0.7083031	4.8371591
H	0.2263779	2.2271613	3.7294073
H	1.5113452	1.0070276	3.7509790
H	1.0588666	1.7884844	2.2265745

E$^+$: [(Me_2Si)(Dipp)N]BMe[N(Dipp)($SiMe_2$F)]$^+$

84

Energy = -1948.824419673

B	0.2084220	1.0310298	-0.3915456
N	0.9914863	-0.0338633	0.1857272
N	-1.2278700	0.8399983	-0.5273838
C	0.8902222	2.3465160	-0.9335745
Si	0.3755153	-1.5898796	0.6796675
C	2.4195904	0.1677187	0.4089214
Si	-2.2831266	2.2013467	-0.9730380
C	-1.7498591	-0.4499051	-0.2568290
H	1.0523799	2.2463589	-2.0152838
H	0.2940076	3.2547985	-0.7996107
H	1.8714107	2.5225005	-0.4851301
C	1.2119350	-2.2647951	2.2020151
C	0.4370540	-2.9547024	-0.5837671
C	2.8484666	0.7469734	1.6247658
C	3.3389340	-0.1893617	-0.5984901
F	-3.7598531	1.5209678	-0.9407158
C	-2.0043886	2.8517249	-2.6987013
C	-2.2638610	3.5364653	0.3238381
C	-1.6609943	-0.9618067	1.1013499
C	-2.2966504	-1.2445562	-1.2839454
H	2.1644289	-2.6852263	1.8533730
H	0.6261770	-3.0851901	2.6285787
H	1.4352378	-1.5385392	2.9814765

H	1.5035633	-3.1525214	-0.7463371
H	-0.0136083	-2.7285184	-1.5483213
H	-0.0014971	-3.8741631	-0.1872584
C	4.2163652	0.9763144	1.8009443
C	1.8668676	1.1449052	2.7164034
C	4.6984851	0.0607285	-0.3721877
C	2.9170771	-0.8017042	-1.9248284
H	-2.1660706	3.9355166	-2.7210625
H	-2.7105115	2.3957206	-3.4010570
H	-0.9911210	2.6578000	-3.0619468
H	-3.1426194	4.1773545	0.1802688
H	-2.3015344	3.1309266	1.3388170
H	-1.3781074	4.1743779	0.2436480
C	-2.1472521	-2.2841151	1.3505118
C	-1.7039005	0.0586540	2.2611564
C	-2.7704788	-2.5212674	-0.9555894
C	-2.3651898	-0.7657671	-2.7214877
C	5.1377486	0.6402577	0.8122139
H	4.5662369	1.4265105	2.7248080
C	1.4591253	2.6264145	2.6056550
C	2.3903836	0.8456754	4.1299565
H	0.9603602	0.5531924	2.5647717
H	5.4198229	-0.1988388	-1.1420781
C	3.6306087	-2.1418483	-2.1808133
C	3.1817201	0.1627787	-3.0967551
H	1.8394186	-0.9863850	-1.8831427
C	-2.6790351	-3.0532687	0.3322090
H	-2.1090650	-2.6672072	2.3648433
C	-1.3355547	-0.4900496	3.6424718
C	-3.1511762	0.5929493	2.3158411
H	-1.0328708	0.8835255	2.0078518
H	-3.2040283	-3.1325275	-1.7417419
C	-3.7745678	-0.9236541	-3.3141224
C	-1.3094514	-1.4811190	-3.5867103
H	-2.1126148	0.2960011	-2.7295402
H	6.1961275	0.8286664	0.9677684
H	0.9820628	2.8417965	1.6461781
H	0.7552051	2.8844502	3.4047373
H	2.3368007	3.2748405	2.6990651
H	1.5941710	1.0129130	4.8628192
H	2.7358807	-0.1893231	4.2236024
H	3.2246494	1.5024118	4.3965218
H	4.7035510	-1.9875313	-2.3354047
H	3.5156221	-2.8315089	-1.3382289
H	3.2279245	-2.6208677	-3.0796022
H	2.8273167	-0.2761365	-4.0358692
H	2.6766001	1.1209175	-2.9495824
H	4.2544336	0.3594687	-3.1978572
H	-3.0377686	-4.0569817	0.5332268
H	-0.3344191	-0.9185066	3.6851394
H	-1.3788134	0.3300574	4.3650003
H	-2.0496850	-1.2506510	3.9738138
H	-3.4892274	0.9764598	1.3512767
H	-3.8321857	-0.2102234	2.6170571
H	-3.2196454	1.3957328	3.0563508
H	-4.0577378	-1.9777278	-3.4034562
H	-4.5184527	-0.4139797	-2.6959448
H	-3.7988927	-0.4888385	-4.3181123
H	-1.3809637	-1.1306722	-4.6208362
H	-0.2955895	-1.2735070	-3.2288440
H	-1.4648129	-2.5657404	-3.5831524

PMe_3H^+ : protonated PMe_3
14
Energy = -461.6569684041

P	0.0011690	-0.0032839	0.2802663
C	0.6207856	1.5917874	-0.2689718
H	1.6366486	1.7358017	0.1075606
H	0.6269076	1.6147775	-1.3622658
H	-0.0288935	2.3838644	0.1118244
C	1.0707003	-1.3361858	-0.2759869
H	1.0839750	-1.3502700	-1.3693856
H	2.0828336	-1.1708701	0.1018702
H	0.6884706	-2.2887853	0.0994693
C	-1.6896982	-0.2617630	-0.2711461
H	-2.0509418	-1.2221858	0.1050490
H	-2.3225478	0.5442013	0.1088823
H	-1.7121978	-0.2629030	-1.3645062
H	0.0024419	-0.0065146	1.6767779

PMe_3 : phospine
13
Energy = -461.2159442040

P	0.0001575	0.0001849	0.6105560
C	0.5923610	1.5223980	-0.2651978
H	1.6253201	1.7338930	0.0295410
H	0.5475695	1.4071677	-1.3550281
H	-0.0263804	2.3761824	0.0294596
C	1.0221467	-1.2741256	-0.2649597
H	0.9455752	-1.1772494	-1.3548243
H	2.0708423	-1.1662458	0.0304174
H	0.6879175	-2.2743311	0.0291710
C	-1.6144261	-0.2481936	-0.2648697
H	-2.0444062	-1.2109805	0.0297183
H	-2.3141177	0.5404945	0.0301676
H	-1.4925592	-0.2291944	-1.3547665

$Et_3SiB(C_6F_5)_4$: ionic Si..F complex

67

Energy = -3465.080629600

Si	6.3252032	0.2821843	0.0543389
C	6.4870347	0.5072296	-1.7749752
H	5.5290839	0.2589665	-2.2485696
H	7.1794135	-0.2901340	-2.0909615
C	7.0016366	1.8816949	-2.2456345
C	5.5342744	-1.2825225	0.6548702
C	7.5835539	1.0827095	1.1506136
H	7.9863184	2.1043972	-1.8245047
H	7.0901879	1.8951380	-3.3353268
H	6.3166146	2.6831062	-1.9544985
H	6.4022998	-1.9357476	0.8490589
H	5.0924172	-1.1108024	1.6440324
C	4.5475083	-1.9930149	-0.2930580
H	8.5409985	0.6036278	0.8916831
H	7.6901542	2.1317361	0.8453928
C	7.3189138	0.9696739	2.6657515
H	5.0081276	-2.2025714	-1.2626139
H	4.2337410	-2.9454073	0.1423785
H	3.6486757	-1.3969781	-0.4661240
H	7.2564211	-0.0751779	2.9835259
H	8.1312286	1.4431329	3.2239875
H	6.3851813	1.4661699	2.9447229
F	3.5935524	0.7019845	2.6373184
C	2.9116794	0.8047215	1.4817635
C	3.5316798	1.2934971	0.3481382
C	1.5773301	0.4078385	1.3790801
C	2.8598273	1.4373134	-0.8542017
F	4.9039318	1.6382321	0.4171919
C	0.8435708	0.4708398	0.1917672
F	1.0404659	-0.0512062	2.5269558
C	1.5300990	1.0322777	-0.8915871
F	3.4875766	1.9322492	-1.9353472
B	-0.7601143	0.0819128	-0.0007304
F	0.9043696	1.1948216	-2.0753124
C	-1.3816786	-0.7852866	1.2599128
C	-1.4249607	1.5834442	-0.1863843
C	-1.0279603	-0.9449100	-1.2687325
C	-0.7726504	-1.9930091	1.6150676
C	-2.5720677	-0.5296980	1.9417836
C	-1.9280402	2.1376288	-1.3645756
C	-1.3818639	2.4785761	0.8871014
C	-2.3493422	-1.2293742	-1.6300776
C	-0.0815849	-1.7112744	-1.9487661
C	-1.2609586	-2.8682743	2.5762706
F	0.3826423	-2.3642067	1.0015926
C	-3.1035930	-1.3785644	2.9129877
F	-3.3082150	0.5797637	1.6854944
C	-2.3898421	3.4500721	-1.4654521
F	-1.9854668	1.4207286	-2.5141190
C	-1.8286888	3.7926881	0.8357389
F	-0.8731714	2.0682668	2.0787491
C	-2.7180026	-2.1567739	-2.5964201
F	-3.3653717	-0.5761084	-1.0125780
C	-0.4008811	-2.6533314	-2.9259526
F	1.2479803	-1.5927098	-1.6850642
C	-2.4449115	-2.5569233	3.2370501
F	-0.6053344	-4.0103851	2.8756803
F	-4.2600535	-1.0684230	3.5367602
C	-2.3444425	4.2864425	-0.3580155
F	-2.8706542	3.9200940	-2.6361508
F	-1.7656598	4.5920295	1.9219539
C	-1.7296716	-2.8791349	-3.2575930
F	-4.0164532	-2.3658256	-2.8991280
F	0.5721085	-3.3543991	-3.5479331
F	-2.9444216	-3.3870468	4.1720385
F	-2.7830558	5.5563081	-0.4401589
F	-2.0568569	-3.7853380	-4.1979426

Et_3SiF : neutral

23

Energy = -627.3455759076

Si	0.1262351	-0.0410219	0.2863480
C	-1.2356683	-1.2944557	0.5990741
H	-2.0602467	-0.7912769	1.1224802
H	-0.8546427	-2.0556822	1.2938321
C	-1.7656710	-1.9711643	-0.6824648
C	-0.4398408	1.3544622	-0.8340344
C	1.6971413	-0.8568698	-0.3370563
H	-0.9695792	-2.5091334	-1.2091831
H	-2.5567130	-2.6933566	-0.4524013
H	-2.1824129	-1.2347231	-1.3787971
H	0.3799074	2.0808237	-0.9224616
H	-0.5865254	0.9455102	-1.8443211
C	-1.7227922	2.0688318	-0.3636932
H	1.9752353	-1.6455058	0.3766785
H	1.4622309	-1.3742793	-1.2785811
C	2.8862184	0.1014390	-0.5499637
H	-1.5928408	2.4939595	0.6374319
H	-1.9950848	2.8860962	-1.0408606
H	-2.5710034	1.3766290	-0.3212757
H	3.1450816	0.6213041	0.3787646
H	3.7767983	-0.4393305	-0.8894123
H	2.6535910	0.8628338	-1.3023779
F	0.4807691	0.6371189	1.7360324

Et_3SiH : neutral silane

23
Energy = -527.9782051161

Si	0.1342461	-0.0256757	0.3276693
C	-1.2405642	-1.2941766	0.6046166
H	-2.0740033	-0.7986183	1.1215090
H	-0.8688192	-2.0595209	1.3002825
C	-1.7528832	-1.9641791	-0.6855775
C	-0.4446095	1.3480791	-0.8349718
C	1.6919632	-0.8593695	-0.3440950
H	-0.9483420	-2.4972093	-1.2046425
H	-2.5470729	-2.6892788	-0.4730590
H	-2.1597273	-1.2236825	-1.3837013
H	0.3703967	2.0766085	-0.9451314
H	-0.5994494	0.9174875	-1.8346986
C	-1.7255127	2.0679738	-0.3714909
H	1.9740704	-1.6647111	0.3489252
H	1.4437745	-1.3522237	-1.2947923
C	2.8854678	0.0934898	-0.5495326
H	-1.5910994	2.5152916	0.6202160
H	-2.0088662	2.8702961	-1.0627315
H	-2.5706015	1.3734403	-0.3065041
H	3.1603932	0.5909638	0.3877536
H	3.7696834	-0.4427998	-0.9135459
H	2.6488351	0.8751295	-1.2799326
H	0.4663017	0.5948758	1.6475114

Et_3SiMe : neutral
26
Energy = -567.3299471862

Si	0.1347755	-0.0169234	0.3773462
C	-1.2347088	-1.3057144	0.6009238
H	-2.0793404	-0.8293489	1.1189934
H	-0.8660448	-2.0833198	1.2855805
C	-1.7291149	-1.9553219	-0.7058187
C	-0.4508980	1.3423579	-0.8047952
C	1.6758562	-0.8519491	-0.3396695
H	-0.9144954	-2.4702412	-1.2274831
H	-2.5190269	-2.6920979	-0.5175777
H	-2.1349577	-1.2044140	-1.3933460
H	0.3526033	2.0856223	-0.9065297
H	-0.5794962	0.9036464	-1.8047410
C	-1.7529341	2.0458728	-0.3760316
H	1.9678414	-1.6696675	0.3354471
H	1.4031505	-1.3310228	-1.2907836
C	2.8762386	0.0882383	-0.5633481
H	-1.6438537	2.5197396	0.6061259
H	-2.0445810	2.8262719	-1.0888346
H	-2.5840058	1.3349081	-0.3061211
H	3.1927746	0.5588653	0.3743755
H	3.7401075	-0.4501702	-0.9709493
H	2.6261891	0.8912754	-1.2657504
C	0.5434746	0.7366574	2.0596288
H	0.9037676	-0.0305160	2.7561704
H	1.3215276	1.5051227	1.9773022
H	-0.3397445	1.2051315	2.5102797

$Et_3Si(toluene)^+$: toluene-coordinated silylium
37
Energy = -798.9330404056

Si	1.1473862	0.0887847	0.3995013
C	1.8979260	-1.5991466	0.7053897
H	1.1142614	-2.3067349	0.9999763
H	2.5180128	-1.4506686	1.6037257
C	2.7611500	-2.1791744	-0.4286741
C	-0.0191125	0.6768707	1.7368038
C	2.4218928	1.3567529	-0.1372666
H	3.5455266	-1.4803801	-0.7363179
H	3.2467533	-3.1048270	-0.1057942
H	2.1585774	-2.4196662	-1.3114448
H	0.5677147	1.3810143	2.3442150
H	-0.8136669	1.2837256	1.2843921
C	-0.6153872	-0.4187372	2.6393071
H	3.3063112	1.1377448	0.4802776
H	2.7303080	1.1551829	-1.1708063
C	2.0236817	2.8330777	0.0377790
H	0.1691163	-0.9708871	3.1651037
H	-1.2748046	0.0242404	3.3919978
H	-1.2033896	-1.1399918	2.0638598
H	1.8239623	3.0673317	1.0873774
H	2.8295130	3.4889195	-0.3053455
H	1.1244998	3.0860824	-0.5332999
H	0.3712048	1.7105219	-2.2393979
C	-0.3495233	1.0731194	-1.7393909
C	0.0126685	-0.2638821	-1.4131914
C	-1.6026504	1.5558499	-1.4076385
C	-0.9798484	-1.1226529	-0.8608500
H	0.8786935	-0.7065214	-1.9073590
C	-2.5600155	0.7200759	-0.8004574
H	-1.8605949	2.5865891	-1.6303205
C	-2.2304434	-0.6242569	-0.5426172
H	-0.7416347	-2.1652569	-0.6772117
C	-3.9249448	1.2433886	-0.4675863
H	-2.9745083	-1.2788431	-0.0994299
H	-4.5998991	1.0605118	-1.3142531
H	-4.3455549	0.7328915	0.4023196
H	-3.9063854	2.3205731	-0.2867347

Et_3Si^+ : silylium cation
22
Energy = -527.1601673289

Si	1.5515919	0.1143031	0.7351202
C	1.8883946	-1.6922739	0.6138199
H	0.9245781	-2.1685663	0.3707792
H	2.1012072	-2.0215233	1.6450993
C	2.9988619	-2.1367064	-0.3566936
C	0.2196494	0.7300471	1.8481205
C	2.5611491	1.3068327	-0.2399477
H	3.9624794	-1.6942342	-0.0888324
H	3.1048751	-3.2242078	-0.3224494
H	2.7640089	-1.8516417	-1.3860505
H	0.7122690	1.4376950	2.5362150
H	-0.4292392	1.3662985	1.2241783
C	-0.5965648	-0.3251359	2.6181503
H	3.6082639	1.1116613	0.0470442
H	2.5008482	0.9723890	-1.2885987
C	2.2109758	2.8005316	-0.1028309
H	0.0443593	-0.9281336	3.2675815
H	-1.3421274	0.1709655	3.2451219
H	-1.1225040	-0.9972653	1.9342822
H	2.3109634	3.1395262	0.9321703
H	2.8881425	3.3961570	-0.7206821
H	1.1876162	3.0004728	-0.4326181

tBu_3PH^+ : protonated phosphine
41
Energy = -815.6776497003

P	0.0007423	-0.0019060	0.4800653
C	0.0319790	1.8183729	0.9257098
C	1.4385907	2.4111304	0.7014236
C	-0.9217360	2.5696945	-0.0324508
C	-0.3818191	2.0375111	2.3936456
H	2.1860438	2.0008759	1.3815958
H	1.7769097	2.2880515	-0.3308819
H	1.3629280	3.4852501	0.9012566
H	-1.9704646	2.3241718	0.1217317
H	-0.7966714	3.6401426	0.1625744
H	-0.6624075	2.3871156	-1.0797651
H	-0.3208570	3.1110924	2.6005443
H	-1.4076950	1.7200595	2.5899334
H	0.2851252	1.5243908	3.0909023
C	1.5606064	-0.9386843	0.9294792
C	1.3710718	-2.4535550	0.7069155
C	2.6904210	-0.4902204	-0.0267247
C	1.9541827	-0.6881149	2.3979750
H	0.6403568	-2.8947269	1.3859803
H	1.0980484	-2.6865063	-0.3256687
H	2.3385425	-2.9249616	0.9100944
H	3.0024475	0.5407382	0.1269178
H	3.5540370	-1.1341471	0.1708400
H	2.4048380	-0.6244400	-1.0745353
H	2.8512321	-1.2798340	2.6086371
H	2.1946997	0.3588565	2.5926380
H	1.1736053	-1.0054175	3.0939029
C	-1.5906947	-0.8841709	0.9290572
C	-1.7660616	-2.0875954	-0.0266268
C	-1.5713859	-1.3494660	2.3978106
C	-2.8080683	0.0368637	0.7051940
H	-1.7391261	-1.7736574	-1.0745548
H	-1.0289444	-2.8728981	0.1279439
H	-2.7553664	-2.5139959	0.1706696
H	-1.4576350	-0.5142716	3.0933626
H	-2.5319061	-1.8316508	2.6077614
H	-0.7842541	-2.0801857	2.5936645
H	-3.7000269	-0.5654396	0.9080693
H	-2.8256832	0.8907638	1.3837644
H	-2.8727555	0.3892909	-0.3276303
H	0.0008602	-0.0036118	-0.9236981

tBu_3P : phosphine
40
Energy = -815.2241801962

P	-0.0005147	0.0005168	0.1682493
C	0.0616458	1.7843662	0.8777706
C	1.0446641	2.5850624	-0.0085989
C	-1.3107472	2.4545294	0.6641128
C	0.4580651	1.9515706	2.3541440
H	2.0812160	2.2687268	0.1079089
H	0.7746538	2.4944324	-1.0658797
H	0.9846416	3.6452784	0.2718919
H	-2.0805848	2.0621731	1.3313539
H	-1.2046720	3.5266575	0.8751795
H	-1.6528504	2.3490532	-0.3707551
H	0.4100272	3.0175846	2.6174155
H	-0.2140546	1.4114769	3.0246745
H	1.4790238	1.6141013	2.5473206
C	1.5142977	-0.9455612	0.8754104
C	1.7146266	-2.1971328	-0.0115192
C	2.7811983	-0.0927862	0.6599962
C	1.4631092	-1.3723966	2.3518674
H	0.9224748	-2.9365521	0.1057502
H	1.7697516	-1.9175636	-1.0687535
H	2.6631500	-2.6754994	0.2674903
H	2.8279932	0.7702712	1.3272029
H	3.6565213	-0.7211804	0.8702359
H	2.8597957	0.2560885	-0.3749203

H	2.4105064	-1.8643065	2.6137021
H	1.3328957	-0.5201113	3.0223388
H	0.6604226	-2.0875909	2.5465009
C	-1.5761073	-0.8383396	0.8779882
C	-1.4710011	-2.3616921	0.6617330
C	-1.9179276	-0.5809355	2.3550412
C	-2.7619309	-0.3862207	-0.0066364
H	-1.2094746	-2.6034830	-0.3736914
H	-0.7460359	-2.8338882	1.3276451
H	-2.4525722	-2.8058608	0.8728246
H	-2.1363942	0.4716770	2.5498628
H	-2.8167747	-1.1561213	2.6183799
H	-1.1134681	-0.8935761	3.0243570
H	-3.6496823	-0.9691239	0.2736206
H	-3.0065534	0.6693274	0.1116715
H	-2.5493340	-0.5729717	-1.0644408

Toluene : PhMe
15
Energy = -271.7318154766

H	1.7288247	2.1596916	0.0053201
C	1.1969188	1.2119250	0.0038076
C	1.9082731	0.0103424	0.0115727
C	-0.1987892	1.2006822	-0.0108792
C	1.2112374	-1.1995221	0.0015524
H	2.9946176	0.0168239	0.0203125
C	-0.9112635	-0.0063220	-0.0158195
H	-0.7444757	2.1418125	-0.0211012
C	-0.1845589	-1.2047603	-0.0130918
H	1.7542708	-2.1409613	0.0013078
C	-2.4204710	-0.0152056	0.0009844
H	-0.7190671	-2.1522510	-0.0250222
H	-2.7979624	-0.0124010	1.0317930
H	-2.8281495	0.8685252	-0.4996164
H	-2.8175583	-0.9084475	-0.4911140

TS1^{+} : TS for fluoride abstraction from **1**
93
Energy = -2671.067496203

B	1.4039179	0.0760249	-0.1513965
N	2.0586986	-0.0448699	-1.3947240
N	1.8955179	-0.0295854	1.1974098
F	-0.0002939	0.4444116	-0.2514940
Si	3.5614432	-1.0382354	-1.4804337
Si	1.4549609	0.7093030	-2.9098961
Si	2.4307752	1.5377003	1.8605718
Si	1.8594654	-1.4682666	2.2309296
C	4.8956109	-0.3913236	-0.3367747
C	3.1026154	-2.8275299	-1.1452082
C	4.3268781	-1.0668513	-3.1974787
C	2.8458758	1.6447198	-3.7700086
C	0.7014616	-0.6195178	-4.0013505
C	0.1566721	2.0272614	-2.5911490
C	2.8340143	2.6829635	0.4265127
C	1.0511560	2.3421431	2.8540352
C	3.9400127	1.3516155	2.9605320
C	1.2697246	-1.0549515	3.9708915
C	0.6640917	-2.7276170	1.5004205
C	3.5659596	-2.2294433	2.4439424
H	5.2198030	0.6071348	-0.6512996
H	4.5789155	-0.3401305	0.7048308
H	5.7678333	-1.0545643	-0.3952324
H	3.9945106	-3.4564755	-1.2563388
H	2.3660329	-3.1630443	-1.8858998
H	2.6889725	-3.0157103	-0.1541986
H	3.6313456	-1.2916329	-4.0109616
H	5.0593279	-1.8850362	-3.1666834
H	4.8712663	-0.1523505	-3.4437370
H	3.5573604	2.0647027	-3.0497632
H	2.3956713	2.4855973	-4.3116710
H	3.4044035	1.0482565	-4.4934876
H	-0.1511701	-1.0917337	-3.4993505
H	1.4148789	-1.4105214	-4.2542403
H	0.3383351	-0.1839219	-4.9401331
H	-0.0081411	2.5536571	-3.5394420
H	-0.8023325	1.6235231	-2.2695712
H	0.4794814	2.7737107	-1.8580229
H	3.1778003	3.6433589	0.8303745
H	3.6217015	2.2846710	-0.2187133
H	1.9593418	2.8921572	-0.1997187
H	1.3960637	3.3049288	3.2514218
H	0.7292215	1.7295562	3.7016430
H	0.1748189	2.5435615	2.2281714
H	3.7668612	0.6975597	3.8205415
H	4.1961880	2.3440916	3.3529406
H	4.8117489	0.9764162	2.4156918
H	1.2769525	-1.9848135	4.5545529
H	0.2501086	-0.6602073	3.9992690
H	1.9222415	-0.3451588	4.4890121
H	0.6800954	-2.7672734	0.4075554
H	0.9194424	-3.7297096	1.8652867
H	-0.3641477	-2.5198635	1.8106556
H	4.2584358	-1.5247270	2.9169308
H	4.0205783	-2.5695611	1.5106104
H	3.4770824	-3.0983013	3.1089588
H	-0.9505240	-1.9958623	-1.0073231
C	-2.0402583	-1.9338635	-1.0519706
H	-2.4455645	-2.4407227	-0.1715979

H	-2.3726501	-2.4864830	-1.9360104
C	-2.5154303	-0.4744488	-1.1319796
H	-2.0765599	0.0241688	-2.0047173
H	-3.6008624	-0.4363538	-1.2868189
Si	-2.1463707	0.5997971	0.3410004
C	-1.7205095	-0.0463138	2.0419565
C	-2.1569960	2.4621483	0.1849442
H	-1.9838933	-1.1070617	2.1030069
H	-0.6245396	-0.0016949	2.0639742
C	-2.2753540	0.7222774	3.2549709
H	-2.5433924	2.8677222	1.1282132
H	-1.0921022	2.7351881	0.1653977
C	-2.8803457	3.1120661	-1.0070414
H	-3.3536442	0.5906927	3.3699324
H	-1.8052385	0.3538625	4.1718843
H	-2.0688316	1.7935315	3.1871255
H	-3.9620686	3.0073265	-0.9165529
H	-2.6476165	4.1799845	-1.0447648
H	-2.5813042	2.6719505	-1.9609700
H	-5.7225688	2.0897362	-0.3166788
C	-5.5563471	1.0361892	-0.1209843
C	-4.8667502	0.6375225	1.0351727
C	-6.0630823	0.0836411	-0.9975464
C	-4.7532239	-0.7329230	1.3216356
H	-4.5603677	1.3813831	1.7653045
C	-5.9166291	-1.2907224	-0.7386665
H	-6.5944580	0.4031078	-1.8900228
C	-5.2674119	-1.6811237	0.4402489
H	-4.2908669	-1.0595185	2.2469451
C	-6.4384138	-2.3160070	-1.7093333
H	-5.1752757	-2.7379913	0.6746848
H	-7.3496907	-1.9657857	-2.2023282
H	-5.6952877	-2.5092116	-2.4935844
H	-6.6464203	-3.2652031	-1.2085630

TS2$^+$: TS for methyl abstraction from **1**

93

Energy = -2671.064459427

B	3.0767263	0.2017712	-0.8158875
N	3.5359549	-0.9295321	-0.0666952
N	2.1190690	1.2002847	-0.3624235
F	3.5737735	0.3767696	-2.0824218
Si	2.2890161	-2.0254772	0.5469241
Si	5.2419461	-1.4060611	-0.2815473
Si	2.5560937	2.1757816	1.0676952
Si	0.8231193	1.6897406	-1.4215636
C	1.1280068	-1.2383990	1.8039475
C	1.2613634	-2.5857317	-0.9306975
C	3.0230057	-3.5518940	1.3520069
C	5.9583354	-2.0085437	1.3505086
C	5.3993127	-2.7283896	-1.6066280
C	6.2404709	0.1112075	-0.7760683
C	3.6949510	1.2605577	2.2400835
C	3.4753600	3.7084403	0.4869985
C	1.0037459	2.6848754	2.0030294
C	0.6363633	3.5360560	-1.6587966
C	0.8468035	0.8300564	-3.0802701
C	-0.8390508	1.1210695	-0.5805329
H	1.6085456	-1.0820593	2.7749984
H	0.7465639	-0.2739872	1.4580420
H	0.2754962	-1.9110424	1.9647417
H	0.5242330	-3.3438034	-0.6401121
H	1.9052180	-3.0222018	-1.7032773
H	0.7169193	-1.7548687	-1.3956716
H	3.7150819	-4.0976067	0.7031835
H	2.1913991	-4.2291690	1.5868681
H	3.5361577	-3.3217596	2.2906272
H	5.6675113	-1.3552062	2.1809646
H	7.0529007	-1.9819594	1.2757772
H	5.6712455	-3.0319021	1.6040543
H	5.0206843	-2.3542160	-2.5648992
H	4.8356402	-3.6334264	-1.3520820
H	6.4479342	-3.0188301	-1.7464273
H	7.3067037	-0.1439437	-0.7308658
H	6.0187829	0.4564835	-1.7891494
H	6.0801274	0.9495239	-0.0869790
H	3.8678845	1.9126977	3.1063991
H	3.2832950	0.3161237	2.6000881
H	4.6670936	1.0518016	1.7846690
H	3.8219416	4.2896158	1.3507320
H	2.8603335	4.3672119	-0.1335900
H	4.3578775	3.4249603	-0.0995383
H	0.3167297	3.2943876	1.4054876
H	1.2991961	3.2961738	2.8646376
H	0.4578098	1.8190903	2.3933774
H	-0.2472699	3.7350544	-2.2783372
H	1.5074216	3.9429527	-2.1839944
H	0.5180670	4.0879089	-0.7214514
H	0.9554819	-0.2559823	-3.0019558
H	-0.0937887	1.0393328	-3.6049106
H	1.6693473	1.2033660	-3.6973941
H	-0.9344815	1.7358681	0.3187623
H	-0.7381384	0.0538156	-0.3701421
H	-1.5734243	1.3492477	-1.3617750
H	-1.5336772	-2.0120296	-0.1474622
C	-2.6182665	-2.1389936	-0.2220698
H	-2.9982896	-2.3807877	0.7743525
H	-2.8044569	-2.9990762	-0.8715850

C -3.3064661 -0.8848761 -0.7886849
H -2.9631082 -0.6731075 -1.8091589
H -4.3892975 -1.0534247 -0.8657931
Si -3.0845442 0.6511317 0.2379475
C -2.6100795 0.4946081 2.0405776
C -3.7816291 2.2835605 -0.3519850
H -3.2720834 -0.2515515 2.4940696
H -1.6045709 0.0570204 2.0874362
C -2.6687953 1.8169598 2.8278642
H -4.6698656 2.5095906 0.2489800
H -3.0523396 3.0643568 -0.0979796
C -4.1238224 2.3256646 -1.8521316
H -3.6849138 2.2222643 2.8495854
H -2.3479297 1.6578194 3.8611654
H -2.0161596 2.5798555 2.3918027
H -4.5129769 3.3099048 -2.1270497
H -3.2438159 2.1346065 -2.4778780
H -4.8786498 1.5762466 -2.1074497
H -6.7763614 1.4398687 -0.2094939
C -6.5672709 0.4110567 0.0677406
C -5.9353762 0.1260973 1.2854818
C -6.9487292 -0.6237729 -0.7822950
C -5.7186771 -1.2072854 1.6493479
H -5.6888038 0.9289479 1.9756087
C -6.7188380 -1.9655553 -0.4357184
H -7.4376657 -0.3921330 -1.7252073
C -6.1061141 -2.2388210 0.7951122
H -5.2634977 -1.4447680 2.6065691
C -7.1136501 -3.0792324 -1.3710124
H -5.9337890 -3.2716263 1.0862944
H -8.1427121 -2.9501010 -1.7220232
H -6.4678118 -3.0869807 -2.2576374
H -7.0305971 -4.0534899 -0.8828024

TS3⁺ : TS for almost barrierless fluoride shift
52
Energy = -1831.975467242
B 0.0881915 0.2324633 0.6810470
N -1.1646385 0.0127708 0.0995043
N 1.4645662 0.0802312 0.1090066
F 0.1536437 0.6868787 1.9797940
Si -1.5188145 -1.2778330 -1.0873427
Si -2.5371951 0.8482370 0.9389179
Si 2.0159787 1.1192023 -1.2939212
Si 2.5075966 -0.7486195 1.1160363
C -2.3896067 -0.6185538 -2.6173417
C 0.0559815 -2.1634876 -1.5972912
C -2.5977199 -2.5519758 -0.2277464
C -4.0534897 0.8536289 -0.1630823
C -2.9235771 -0.0278624 2.5497942
C -2.0479871 2.6396183 1.2130606
C 3.1915354 0.1140178 -2.3504455
C 0.5250037 1.6487202 -2.2771123
C 2.8538208 2.6068706 -0.5289892
C 1.7958937 -1.9257706 2.3354245
C 4.3295429 -0.5493675 1.0615568
H -3.4769354 -0.6578926 -2.5070392
H -2.1157357 0.4141073 -2.8516205
H -2.1239122 -1.2394340 -3.4812880
H -0.2364470 -2.9940565 -2.2517563
H 0.5874524 -2.5962642 -0.7431787
H 0.7534121 -1.5377616 -2.1584349
H -2.0896181 -2.9708914 0.6479402
H -2.8058623 -3.3778098 -0.9187167
H -3.5604761 -2.1506302 0.1013598
H -4.8363267 1.4023355 0.3760778
H -4.4516329 -0.1399184 -0.3870958
H -3.8760709 1.3770697 -1.1073255
H -3.7742259 0.4568556 3.0451511
H -2.0705977 0.0010422 3.2352693
H -3.1912088 -1.0770816 2.3793707
H -1.7830527 3.1218158 0.2640991
H -2.9032661 3.1869652 1.6284651
H -1.2100462 2.7551994 1.9057207
H 4.1894153 0.0150571 -1.9111653
H 2.8107910 -0.8921708 -2.5538965
H 3.3148050 0.6215396 -3.3143605
H 0.0580655 0.8145417 -2.8074394
H 0.8674171 2.3694938 -3.0301216
H -0.2365052 2.1392530 -1.6635618
H 2.1579532 3.1547841 0.1170953
H 3.1917264 3.2924859 -1.3149944
H 3.7290782 2.3355765 0.0719650
H 2.3316443 -2.8796364 2.2739825
H 0.7279833 -2.0948262 2.1781891
H 1.9417092 -1.5345995 3.3493465
H 4.8181457 -1.5281726 1.1355413
H 4.6927675 -0.0117990 0.1853402
H 4.6085352 0.0190460 1.9625583

TS4t⁺ : TS for Me_3Si^+ abstraction with *t*Bu_3P
95
Energy = -2587.225411389
B -2.3793850 0.2982648 -0.0792706
N -3.3453793 -0.6592809 0.0377583
N -1.4542625 1.2214326 -0.1695305

Si	-3.8419392	-1.5321829	-1.4592807
Si	-4.1256392	-0.8264675	1.6572576
Si	-1.9060539	2.9300983	-0.2899023
Si	0.7725196	0.4296573	-0.1056661
C	-2.9349720	-0.7056225	-2.8771288
C	-3.3210993	-3.3259335	-1.3262620
C	-5.6942490	-1.3613962	-1.6576714
C	-5.5829482	0.3466077	1.7215759
C	-4.6614889	-2.6012385	1.9016011
C	-2.8347695	-0.3443082	2.9288701
C	-3.5253590	3.0603018	-1.2392084
C	-2.1536693	3.6304552	1.4367297
C	-0.5922884	3.9182579	-1.1985213
C	0.8680940	1.5767573	1.3798388
C	0.0414495	-1.2851896	0.1466831
C	0.8736290	0.9739821	-1.9035000
H	-3.1994205	0.3530768	-2.9710361
H	-1.8461414	-0.7728570	-2.7680359
H	-3.2012869	-1.2019361	-3.8181596
H	-3.5144455	-3.8339368	-2.2791552
H	-3.8679801	-3.8631028	-0.5457808
H	-2.2498826	-3.4145986	-1.1119879
H	-6.2457722	-1.8148302	-0.8268219
H	-6.0157188	-1.8639242	-2.5779838
H	-5.9882018	-0.3082523	-1.7287283
H	-5.2595251	1.3842349	1.5765881
H	-6.0797378	0.2854507	2.6973731
H	-6.3258729	0.1147247	0.9511782
H	-3.8117993	-3.2906326	1.8556078
H	-5.4084135	-2.9236291	1.1687612
H	-5.1163339	-2.6965910	2.8953609
H	-3.2805524	-0.3457775	3.9306936
H	-1.9957636	-1.0493328	2.9388444
H	-2.4347037	0.6595803	2.7468657
H	-3.4102261	2.7117904	-2.2721568
H	-4.3188314	2.4652538	-0.7701586
H	-3.8713674	4.1004670	-1.2754835
H	-2.4555922	4.6834648	1.3766248
H	-1.2450143	3.5780265	2.0454119
H	-2.9458660	3.0896826	1.9686127
H	0.4027173	3.8324614	-0.7493498
H	-0.8737926	4.9784228	-1.1679098
H	-0.5141651	3.6275221	-2.2511850
H	-0.0136419	1.3923150	1.9993906
H	0.8370275	2.6234543	1.0640588
H	1.7519732	1.4324644	1.9952864
H	-0.6797781	-1.4873732	-0.6523675
H	0.7793247	-2.0841412	0.1166366
H	-0.4904501	-1.3444965	1.1012872
H	-0.1113670	1.2959806	-2.2426212
H	1.1763005	0.1280182	-2.5252890
H	1.5816755	1.7870007	-2.0704437
P	3.2978409	-0.3156071	0.0483546
C	3.6009688	-1.4410421	1.5761560
C	3.1064390	-2.8876028	1.3628166
C	5.0811963	-1.5379007	1.9976868
C	2.7784493	-0.8794179	2.7548259
H	2.0437131	-2.9430966	1.1232741
H	3.6704947	-3.4262223	0.6013402
H	3.2508933	-3.4167339	2.3121995
H	5.5001738	-0.5802900	2.3051554
H	5.1346385	-2.2109740	2.8622683
H	5.7101371	-1.9574630	1.2114343
H	2.9368833	-1.5327916	3.6208569
H	3.0772125	0.1290841	3.0429081
H	1.7080753	-0.8785387	2.5295216
C	3.8310958	-1.2890134	-1.5139923
C	5.1354074	-2.0954777	-1.3549943
C	2.6968267	-2.2731028	-1.8782987
C	4.0137520	-0.3542461	-2.7291991
H	5.9880169	-1.4686151	-1.0928681
H	5.0489032	-2.8941766	-0.6176205
H	5.3517620	-2.5661260	-2.3219565
H	1.7600584	-1.7554011	-2.0955814
H	2.9968657	-2.8106121	-2.7854236
H	2.5101113	-3.0147051	-1.1012569
H	4.1605418	-0.9914683	-3.6092837
H	3.1428012	0.2731939	-2.9176623
H	4.8919197	0.2860504	-2.6406150
C	4.4385247	1.2236424	0.2127798
C	4.4319612	1.8324538	1.6320201
C	5.9126148	0.9600384	-0.1544517
C	3.8787615	2.3198985	-0.7183765
H	3.4393282	2.1506948	1.9502144
H	4.8533256	1.1726413	2.3899861
H	5.0612491	2.7296400	1.5941609
H	6.0446166	0.6670201	-1.1959773
H	6.4614878	1.8982147	-0.0074741
H	6.3725989	0.2033800	0.4826252
H	4.5293635	3.1988373	-0.6397893
H	3.8492108	2.0195324	-1.7659825
H	2.8744888	2.6233589	-0.4087722

TS4$^+$: TS for Me_3Si^+ abstraction with PMe_3

68

Energy = -2233.222613271

B	-1.2081861	0.4217762	-0.1022159

N	-2.1873505	-0.5435230	-0.0175831
N	-0.2697661	1.3127365	-0.1547543
Si	-2.6615248	-1.3849958	-1.5285081
Si	-3.0522601	-0.6551202	1.5536379
Si	-0.3945579	3.0407994	-0.3132102
Si	2.2846353	-0.1298556	0.0466066
C	-1.6520686	-0.6115131	-2.9080750
C	-2.2557016	-3.2104200	-1.3904539
C	-4.4932639	-1.1257135	-1.8205964
C	-4.4609304	0.5804887	1.5459290
C	-3.6845391	-2.4011215	1.7910425
C	-1.8172291	-0.2101653	2.8938978
C	-2.0290218	3.4888605	-1.1376929
C	-0.3336050	3.8319494	1.3936214
C	1.0092495	3.7191219	-1.3658363
C	2.4210051	1.1601496	1.4053637
C	1.1513308	-1.5953249	0.3690787
C	2.3939659	0.4060292	-1.7445761
H	-1.8439546	0.4632456	-3.0001987
H	-0.5753770	-0.7478998	-2.7533015
H	-1.9113453	-1.0831396	-3.8637234
H	-2.4524366	-3.7029303	-2.3509464
H	-2.8586546	-3.7140179	-0.6287981
H	-1.1996231	-3.3687926	-1.1444054
H	-5.1061521	-1.5485424	-1.0168169
H	-4.7950789	-1.6142714	-2.7551106
H	-4.7314370	-0.0594831	-1.9051828
H	-4.0816901	1.6013898	1.4174861
H	-5.0105977	0.5446540	2.4943751
H	-5.1722189	0.3818935	0.7372619
H	-2.8653431	-3.1279008	1.8109106
H	-4.3943841	-2.7032740	1.0137794
H	-4.2091760	-2.4600712	2.7527575
H	-2.3198580	-0.1699687	3.8677640
H	-1.0113149	-0.9492005	2.9655750
H	-1.3625092	0.7706777	2.7124702
H	-2.0994544	3.0452024	-2.1384360
H	-2.8815489	3.1250426	-0.5506443
H	-2.1393143	4.5750082	-1.2458488
H	-0.4212623	4.9229373	1.3174061
H	0.6013388	3.6072304	1.9182067
H	-1.1616576	3.4743250	2.0178705
H	1.9950522	3.4946211	-0.9440021
H	0.9222010	4.8109372	-1.4330081
H	0.9787366	3.3229979	-2.3873825
H	1.4888936	1.1900286	1.9737928
H	2.5879962	2.1502259	0.9716359
H	3.2435039	0.9598271	2.1009199
H	0.3981505	-1.6652657	-0.4202415
H	1.6849192	-2.5514643	0.3941562
H	0.6263755	-1.4784450	1.3211856
H	1.4491938	0.8663155	-2.0404376
H	2.5749223	-0.4573242	-2.3948608
H	3.1973506	1.1327979	-1.9053166
P	4.3789020	-1.1451287	0.2731662
C	4.5745177	-1.9383542	1.8985135
H	3.8114754	-2.7109036	2.0242867
H	5.5657792	-2.3949839	1.9703419
H	4.4630483	-1.1938850	2.6908466
C	4.6798274	-2.4371722	-0.9706113
H	4.6524186	-1.9989453	-1.9711875
H	5.6600128	-2.8922061	-0.8020152
H	3.9080281	-3.2078008	-0.8956408
C	5.7269919	0.0641714	0.1103988
H	5.6127306	0.8437423	0.8680290
H	6.6926471	-0.4316209	0.2431917
H	5.6883117	0.5213123	-0.8817935

TS5t^{+} : TS for **7**$^{+}$ deprotonation with *t*Bu$_3$P
95
Energy = -2587.208082980

B	-0.0738238	-0.1158836	1.7747566
N	-0.0127618	1.1541094	2.3861317
N	-0.1434511	-1.4589344	2.1423170
Si	1.5704003	1.7342215	2.9473157
Si	-1.5098795	2.1115946	2.4023700
Si	-0.1637153	-2.2871728	3.7167248
Si	-0.1507320	-1.9662259	0.4527781
C	2.8783843	0.6673879	2.1149898
C	1.8105571	3.5215310	2.4324873
C	1.7120681	1.5880959	4.8119478
C	-1.6917666	2.9444101	4.0703390
C	-1.5065783	3.3952223	1.0308854
C	-2.9316789	0.9159750	2.1068959
C	-1.0917556	-1.2258278	4.9476071
C	-1.0288816	-3.9283794	3.4533072
C	1.6138823	-2.5695321	4.2413391
C	1.3494590	-2.9886889	0.0359017
C	-1.7508065	-2.7816637	-0.0408196
C	-0.0306094	-0.1625872	-0.1125248
H	2.7154539	-0.4041216	2.2768548
H	2.9211021	0.8471892	1.0344280
H	3.8665004	0.9113628	2.5221818
H	2.8060791	3.8578885	2.7475005
H	1.0786492	4.1941657	2.8926919
H	1.7452024	3.6396311	1.3451486
H	0.9304919	2.1620157	5.3211856

H	2.6823713	1.9763959	5.1459220
H	1.6371820	0.5487129	5.1481828
H	-1.7238537	2.2101760	4.8827066
H	-2.6263292	3.5183223	4.0969823
H	-0.8727271	3.6428093	4.2744472
H	-1.3535210	2.9353175	0.0481824
H	-0.7235227	4.1468061	1.1707360
H	-2.4711906	3.9171001	1.0036350
H	-3.8823542	1.4614714	2.1434785
H	-2.8788920	0.4326409	1.1234436
H	-2.9690277	0.1279568	2.8658835
H	-0.6858422	-0.2107243	4.9904507
H	-2.1539289	-1.1567613	4.6878915
H	-1.0218555	-1.6606303	5.9519544
H	-1.0496528	-4.4967313	4.3908490
H	-0.5139130	-4.5476706	2.7087393
H	-2.0653985	-3.7892035	3.1257564
H	2.1634487	-3.1365076	3.4806435
H	1.6503664	-3.1423261	5.1759150
H	2.1475053	-1.6278815	4.4084208
H	1.3308738	-3.3520197	-0.9959982
H	1.3699937	-3.8671822	0.6936539
H	2.2786919	-2.4311533	0.1962585
H	-2.6135136	-2.1354563	0.1513789
H	-1.7560237	-3.0678003	-1.0970496
H	-1.8818942	-3.6978928	0.5459589
H	0.9084668	0.3949457	-0.1165623
H	-0.8939399	0.4992904	-0.2104181
P	0.0948162	-0.0225623	-3.3151537
C	-0.0791541	1.8672173	-3.4798813
C	-1.5201749	2.2869760	-3.1261414
C	0.8262005	2.5354107	-2.4182862
C	0.2623071	2.4317260	-4.8701762
H	-2.2418083	1.9944873	-3.8899670
H	-1.8440609	1.8819929	-2.1625943
H	-1.5417795	3.3805849	-3.0515386
H	1.8878798	2.3564781	-2.5805504
H	0.6592972	3.6181644	-2.4650167
H	0.5679017	2.2052259	-1.4086217
H	0.0803317	3.5139940	-4.8583443
H	1.3109530	2.2784945	-5.1327177
H	-0.3587231	1.9961584	-5.6555349
C	-1.3921945	-0.8757331	-4.1363962
C	-1.0946484	-2.3847165	-4.2529668
C	-2.5972051	-0.7496338	-3.1743641
C	-1.7900284	-0.3397810	-5.5220777
H	-0.3244549	-2.6070973	-4.9932112
H	-0.7946441	-2.8162042	-3.2925345
H	-2.0162443	-2.8861232	-4.5709223
H	-3.0083577	0.2576606	-3.1363433
H	-3.3894669	-1.4194791	-3.5289355
H	-2.3320653	-1.0543695	-2.1590963
H	-2.6335188	-0.9348191	-5.8949088
H	-2.1168402	0.7014706	-5.4808651
H	-0.9770388	-0.4203598	-6.2461532
C	1.7166583	-0.5856859	-4.1372712
C	2.0842409	-1.9660488	-3.5490974
C	1.6733243	-0.6683272	-5.6728996
C	2.8675101	0.3538293	-3.7226848
H	2.2010119	-1.9012376	-2.4639412
H	1.3568675	-2.7444066	-3.7770314
H	3.0484196	-2.2738983	-3.9716952
H	1.4164989	0.2891322	-6.1308515
H	2.6690614	-0.9572754	-6.0321259
H	0.9665115	-1.4223795	-6.0253691
H	3.8036622	-0.0921989	-4.0787510
H	2.7872999	1.3479657	-4.1643873
H	2.9410299	0.4517677	-2.6347174
H	0.0353370	-0.3606208	-1.4332674

TS5$^+$: TS for **7$^+$** deprotonation with PMe_3
68
Energy = -2233.195703960

B	-0.0377237	0.1020496	0.4700547
N	0.0483706	1.2136189	1.3578695
N	-0.0954277	-1.2975659	0.6629101
Si	1.6191170	1.5933311	2.0819269
Si	-1.3891879	2.2395439	1.4919821
Si	-0.1730926	-2.3660497	2.0793328
Si	-0.1647739	-1.5396725	-1.0636630
C	2.9213655	0.6305748	1.1217417
C	1.9726831	3.4288521	1.9048253
C	1.6821902	1.1293549	3.8998316
C	-1.5759190	2.8208627	3.2646635
C	-1.2935174	3.7161472	0.3319195
C	-2.8743210	1.1851284	1.0155158
C	-1.1518739	-1.5203559	3.4315532
C	-1.0372196	-3.9300108	1.5082844
C	1.5748673	-2.7721111	2.6217761
C	1.3314871	-2.3923147	-1.7690400
C	-1.7948565	-2.1675602	-1.7017556
C	-0.0562551	0.3803577	-1.2518412
H	2.7343454	-0.4485545	1.1203608
H	2.9812331	0.9669615	0.0792497
H	3.9084876	0.7913543	1.5714175
H	2.9633468	3.6498539	2.3214691
H	1.2480231	4.0520654	2.4399770
H	1.9746759	3.7370384	0.8533343

H	0.9151657	1.6587347	4.4753665
H	2.6594264	1.4019106	4.3179950
H	1.5401522	0.0560543	4.0608167
H	-1.6739123	1.9737309	3.9523489
H	-2.4805261	3.4346788	3.3559073
H	-0.7304520	3.4329664	3.5968350
H	-1.1592842	3.4035736	-0.7106727
H	-0.4639334	4.3828654	0.5886287
H	-2.2210588	4.2998371	0.3848744
H	-3.7937936	1.7724096	1.1273512
H	-2.8390443	0.8445999	-0.0265149
H	-2.9627026	0.3009940	1.6555317
H	-0.7576151	-0.5246675	3.6537180
H	-2.2038438	-1.4134572	3.1444119
H	-1.1162331	-2.1139352	4.3528610
H	-1.0988295	-4.6498836	2.3331198
H	-0.4962062	-4.4211802	0.6897154
H	-2.0593886	-3.7256226	1.1698798
H	2.1493040	-3.2113285	1.7974579
H	1.5558055	-3.5014909	3.4406112
H	2.1170132	-1.8897170	2.9764261
H	1.2980048	-2.4724868	-2.8600684
H	1.3817707	-3.4129943	-1.3699448
H	2.2529666	-1.8750845	-1.4817496
H	-2.6292577	-1.6236929	-1.2470300
H	-1.8686822	-2.0725003	-2.7897494
H	-1.9077496	-3.2291638	-1.4552094
H	0.8745156	0.9402476	-1.3763693
H	-0.9212597	1.0341372	-1.3894274
P	0.0389557	-0.0789093	-4.4199482
H	-0.0646166	-0.0291240	-2.5412104
C	-0.8666309	1.3715254	-5.0702656
H	-1.9359546	1.2502382	-4.8747066
H	-0.5191775	2.2761049	-4.5621430
H	-0.7085183	1.4839813	-6.1474538
C	1.7724170	0.1945667	-4.9384978
H	2.3796381	-0.6718323	-4.6613024
H	1.8383514	0.3479935	-6.0202244
H	2.1711055	1.0762883	-4.4279348
C	-0.5341344	-1.4768934	-5.4553897
H	-0.3591482	-1.2667387	-6.5156279
H	0.0033301	-2.3879509	-5.1777649
H	-1.6038935	-1.6375068	-5.2949081

TS6a$^+$: TS for fluoride abstraction from **6**
125
Energy = -2787.865322768

B	-0.5886007	-0.2325488	-0.1730866
N	-0.7847554	1.0643800	-0.7243007
N	-1.4190618	-1.2127169	0.4052923
F	0.8146857	-0.6984366	-0.2139641
Si	0.0399513	1.7332059	-2.1830366
C	-1.6871947	2.0403904	-0.1100795
Si	-0.7476916	-2.6827585	1.2180362
C	-2.8762097	-1.2634974	0.3415534
C	-0.5209282	3.5030239	-2.4506220
C	1.9071631	1.9605967	-2.1041028
C	-0.3246867	0.5644080	-3.6077697
C	-2.9370220	2.3407136	-0.6968372
C	-1.1997529	2.8048592	0.9735570
C	0.1970707	-3.7337440	-0.0263076
C	0.2775591	-2.0955438	2.6794272
C	-2.1357432	-3.7671260	1.8494746
C	-3.4909700	-1.9596044	-0.7254361
C	-3.6368021	-0.8187697	1.4454979
H	-0.0918031	3.8466554	-3.4008126
H	-0.1037247	4.1319894	-1.6556002
H	-1.5946772	3.6769153	-2.4736767
H	2.2062835	2.6840291	-1.3402736
H	2.1800761	2.3934720	-3.0762220
H	2.5062391	1.0599225	-1.9829425
H	0.2442077	0.8524169	-4.4993891
H	-0.0469590	-0.4674210	-3.3576328
H	-1.3853771	0.5546873	-3.8712526
C	-3.6408623	3.4568569	-0.2285109
C	-3.5678851	1.4973067	-1.7933705
C	-1.9488848	3.9015587	1.4138399
C	0.1370800	2.5301301	1.6459331
H	0.7831450	-4.5032052	0.4900451
H	0.8700627	-3.1694653	-0.6754257
H	-0.5153831	-4.2548639	-0.6721482
H	-0.3903476	-1.6850631	3.4436641
H	0.8347788	-2.9202261	3.1390530
H	0.9900071	-1.3089369	2.4185772
H	-2.7803901	-3.2747571	2.5800057
H	-1.6784188	-4.6401808	2.3338740
H	-2.7731926	-4.1293576	1.0380159
C	-4.8632609	-2.2236121	-0.6453358
C	-2.7304858	-2.4988209	-1.9309344
C	-5.0025258	-1.1239733	1.4828497
C	-3.0419063	-0.0269204	2.5986650
C	-3.1493243	4.2467195	0.8045895
H	-4.5935961	3.7073494	-0.6863361
C	-3.6563846	2.1980303	-3.1630526
C	-4.9874502	1.0700734	-1.3831884
H	-2.9548914	0.5979220	-1.8954431
H	-1.5740881	4.5015739	2.2385967
C	-0.0119460	2.3078589	3.1613372

C	1.1280072	3.6777223	1.3758863
H	0.5519483	1.6167248	1.2096550
C	-5.6150932	-1.8334987	0.4564016
H	-5.3440524	-2.7625702	-1.4571287
C	-3.2371413	-1.8944108	-3.2537579
C	-2.8447619	-4.0338787	-2.0208246
H	-1.6740945	-2.2359140	-1.8144776
H	-5.5914596	-0.7992177	2.3360773
C	-3.0801111	-0.7947640	3.9328531
C	-3.7810939	1.3106515	2.7717839
H	-1.9989618	0.1874372	2.3476621
H	-3.7082516	5.1133903	1.1455890
H	-2.6801495	2.3898947	-3.6108635
H	-4.2262708	1.5697189	-3.8567641
H	-4.1822642	3.1549663	-3.0706936
H	-4.9966822	0.6242027	-0.3883943
H	-5.6637990	1.9318907	-1.3838909
H	-5.3780181	0.3374781	-2.0958851
H	-0.6598293	1.4545207	3.3783252
H	0.9671595	2.1199001	3.6142827
H	-0.4404674	3.1898458	3.6478783
H	1.2592220	3.8497799	0.3036388
H	0.7646223	4.6104408	1.8197986
H	2.1065970	3.4547948	1.8147327
H	-6.6744189	-2.0678073	0.5077128
H	-3.1496237	-0.8067983	-3.2660981
H	-2.6596637	-2.2947110	-4.0940055
H	-4.2895245	-2.1502520	-3.4157253
H	-2.5577446	-4.5246113	-1.0873141
H	-2.2056878	-4.4155904	-2.8244772
H	-3.8761992	-4.3265402	-2.2436994
H	-2.5195729	-1.7316189	3.8944589
H	-2.6540726	-0.1766249	4.7310234
H	-4.1133711	-1.0331689	4.2076121
H	-3.8170660	1.8675196	1.8348148
H	-4.8082452	1.1406684	3.1130888
H	-3.2758269	1.9269426	3.5223504
Si	2.9758684	-1.0475947	-0.2653772
C	3.2928166	0.5829542	0.5742307
C	3.1760065	-2.5505218	0.8211123
C	2.7865927	-1.4265974	-2.0960460
C	3.2876346	0.5253090	2.1126213
H	2.5545030	1.3052570	0.2225651
H	4.2659015	0.9440217	0.2146630
H	3.8015197	-2.2439459	1.6666939
H	2.1976779	-2.7755878	1.2560839
C	3.7467163	-3.8135472	0.1510318
H	2.6421258	-2.5149356	-2.1275542
H	1.8223703	-0.9892299	-2.3746874
C	3.8348198	-1.0269741	-3.1499620
H	2.3006858	0.2689843	2.5057313
H	4.0070048	-0.1999572	2.4976397
H	3.5601170	1.5050852	2.5160763
H	3.1023351	-4.1696600	-0.6571060
H	3.8298788	-4.6179888	0.8876225
H	4.7422723	-3.6394082	-0.2656625
H	4.7688804	-1.5742867	-3.0156180
H	3.4478660	-1.2657882	-4.1448555
H	4.0539059	0.0427600	-3.1216686
H	5.9310155	-1.9923075	-1.2071613
C	6.1192336	-1.1025526	-0.6130484
C	6.3462885	0.1285284	-1.2392070
C	6.2629348	-1.2022583	0.7769127
H	6.2857863	0.2113148	-2.3189290
C	6.6718847	1.2497031	-0.4789085
C	6.5967892	-0.0785747	1.5296103
H	6.1426300	-2.1630600	1.2681056
C	6.7933158	1.1675599	0.9170524
H	6.8460399	2.2021384	-0.9727580
H	6.7136488	-0.1685243	2.6065145
C	7.1192283	2.3869210	1.7399649
H	7.5152151	3.1919749	1.1156496
H	6.2197331	2.7634466	2.2432265
H	7.8512241	2.1521182	2.5190139

TS6^{+} : TS for methyl abstraction from **6**
125
Energy = -2787.882687986

B	-2.4260175	-1.0372449	0.7065574
N	-1.0610751	-0.6404864	0.4921231
N	-3.6810423	-0.4940815	0.3328275
F	-2.4969388	-2.2142843	1.4370972
Si	0.1310851	-1.9290283	0.7455272
C	-0.5642918	0.7003073	0.2884729
Si	-5.1275577	-1.0130950	1.2789649
C	-3.9516348	0.2665891	-0.8686323
C	1.8678646	-1.0755133	0.5730230
C	0.1872607	-2.7197284	2.4418522
C	-0.0267788	-3.2350934	-0.5807546
C	-0.1787474	1.1243209	-1.0019652
C	-0.3309349	1.5184585	1.4186881
C	-6.6457890	-0.2462906	0.4881832
C	-5.3832667	-2.8761388	1.2933870
C	-4.8669992	-0.4141877	3.0406257
C	-4.2134989	-0.4378561	-2.0676701
C	-4.0978582	1.6692168	-0.8149677
H	2.5053474	-1.9316417	0.3206993
H	2.0435130	-0.6632722	1.5725898

H	1.8211533	-0.3079495	-0.2022849
H	0.0639366	-1.9904112	3.2475568
H	1.1546039	-3.2196259	2.5823535
H	-0.5993763	-3.4717592	2.5439515
H	0.7112178	-4.0330188	-0.4358216
H	-1.0218604	-3.6917316	-0.5273652
H	0.1015955	-2.8245017	-1.5864090
C	0.5138942	2.3347615	-1.1301513
C	-0.5077356	0.3138977	-2.2435298
C	0.3618519	2.7218105	1.2383174
C	-0.7707050	1.1252893	2.8231572
H	-7.5192927	-0.4873830	1.1072535
H	-6.8209152	-0.6610583	-0.5098707
H	-6.5917935	0.8392269	0.3837556
H	-4.6357098	-3.3881420	1.9047456
H	-6.3746225	-3.0903045	1.7138071
H	-5.3532181	-3.3059012	0.2871781
H	-4.7596804	0.6746450	3.0875769
H	-3.9623320	-0.8618520	3.4695629
H	-5.7120133	-0.6996846	3.6787614
C	-4.6622068	0.2791929	-3.1822570
C	-4.0749145	-1.9504068	-2.1847415
C	-4.5638578	2.3398003	-1.9521753
C	-3.7519056	2.4726975	0.4262616
C	0.8047255	3.1209825	-0.0190616
H	0.8219608	2.6665904	-2.1179121
C	0.7370331	-0.2932220	-2.9142858
C	-1.2752350	1.1673041	-3.2656488
H	-1.1605234	-0.5020435	-1.9232991
H	0.5573436	3.3547720	2.0998141
C	-1.6293230	2.2124426	3.4942457
C	0.4411910	0.8184176	3.7206607
H	-1.3753204	0.2165442	2.7466774
C	-4.8589632	1.6547120	-3.1262489
H	-4.8734298	-0.2549245	-4.1050075
C	-3.0485547	-2.3545887	-3.2586685
C	-5.4303547	-2.6136801	-2.4945769
H	-3.7248447	-2.3342736	-1.2220697
H	-4.6953086	3.4178133	-1.9138138
C	-4.9905401	3.0489366	1.1359081
C	-2.7841055	3.6177339	0.0844591
H	-3.2478394	1.7878585	1.1141152
H	1.3509292	4.0525030	-0.1358892
H	1.3183651	-0.9194828	-2.2306094
H	0.4382204	-0.9171969	-3.7635366
H	1.3942100	0.4957621	-3.2943133
H	-2.1543899	1.6268349	-2.8107061
H	-0.6359394	1.9603650	-3.6698876
H	-1.6072193	0.5433469	-4.1014846
H	-2.5129076	2.4525674	2.8996922
H	-1.9620165	1.8651330	4.4779805
H	-1.0559208	3.1340438	3.6396702
H	1.0655076	0.0240448	3.3013423
H	1.0650846	1.7102133	3.8404484
H	0.1125021	0.4958086	4.7141124
H	-5.2254385	2.1917757	-3.9966194
H	-2.0590078	-1.9431627	-3.0442819
H	-2.9627846	-3.4458424	-3.3042948
H	-3.3555808	-1.9981395	-4.2478347
H	-6.1850362	-2.3579649	-1.7458094
H	-5.3234509	-3.7039075	-2.5164607
H	-5.8043009	-2.2904421	-3.4721908
H	-5.6631367	2.2666694	1.4944803
H	-4.6815028	3.6505687	1.9985671
H	-5.5542291	3.6984085	0.4568545
H	-1.9158436	3.2504939	-0.4661671
H	-3.2823171	4.3800954	-0.5245905
H	-2.4319323	4.1016239	1.0013341
Si	4.1881963	-0.3776466	0.6808067
C	4.4582830	-0.3252122	-1.1585905
C	3.9348890	1.2343633	1.5897116
C	4.7284141	-1.8724119	1.6656060
C	4.0273222	0.9994123	-1.8128433
H	3.9473397	-1.1805136	-1.6179118
H	5.5325061	-0.4955464	-1.3179533
H	4.6510748	1.9544687	1.1762888
H	2.9415241	1.6204291	1.3227341
C	4.0960613	1.1388172	3.1183820
H	5.6581755	-1.6088782	2.1821315
H	3.9894284	-2.0345298	2.4616675
C	4.9219022	-3.1519028	0.8323384
H	2.9724347	1.2219428	-1.6274212
H	4.6178360	1.8340819	-1.4262956
H	4.1772465	0.9549379	-2.8955457
H	3.4352127	0.3839902	3.5545925
H	3.8573790	2.0985452	3.5853384
H	5.1233553	0.8804007	3.3925196
H	5.6903441	-3.0124404	0.0663518
H	5.2259800	-3.9834205	1.4739959
H	3.9986938	-3.4519932	0.3221398
H	6.9723839	0.5410988	1.9350377
C	7.2257773	0.4042501	0.8869749
C	7.7080885	-0.8305698	0.4339842
C	7.1555108	1.4803862	-0.0025766
H	7.8019538	-1.6643966	1.1234153
C	8.0867284	-0.9892780	-0.8963972
C	7.5426501	1.3165950	-1.3326110
H	6.8119773	2.4517005	0.3412787

C	8.0039094	0.0802297	-1.8035971
H	8.4581658	-1.9521028	-1.2383584
H	7.4850674	2.1588951	-2.0170755
C	8.3961089	-0.1045940	-3.2470932
H	9.3818193	-0.5745716	-3.3284820
H	7.6821780	-0.7590093	-3.7619048
H	8.4215202	0.8519221	-3.7752907

TS7^{+} : TS for Si-to-B methyl shift
84
Energy = -1948.785783649

B	-0.8885730	0.0267879	0.7108068
N	-0.8889577	-1.2331139	0.1459292
N	-0.2495178	1.2543750	0.8234929
Si	-2.6007629	-1.4875733	0.4590352
C	0.1456300	-2.0723839	-0.4199284
Si	-0.2660530	2.4042850	2.1956219
C	0.5550027	1.6987863	-0.3312720
C	-3.7572215	-1.2198438	-0.9648155
C	-2.9989696	-2.9804856	1.4778341
C	-2.6600010	0.1205624	1.5860467
C	0.8644210	-2.9240504	0.4441077
C	0.3608635	-2.0624929	-1.8097939
F	0.1696540	1.4804175	3.4577597
C	-1.9361873	3.1186022	2.6111371
C	0.9589189	3.7593973	1.8880896
C	-0.0540232	2.5580309	-1.2657696
C	1.9165885	1.3477263	-0.4095148
H	-3.5095109	-0.3207468	-1.5357531
H	-4.7831680	-1.1235633	-0.5910814
H	-3.7330359	-2.0792617	-1.6430698
H	-2.4630301	-2.9953294	2.4301404
H	-2.7425283	-3.8892974	0.9221034
H	-4.0761372	-3.0059613	1.6794306
H	-3.5314941	-0.3175996	2.1097587
H	-2.0088039	0.2950597	2.4577239
H	-3.0106137	1.0582482	1.1583438
C	1.8291802	-3.7617312	-0.1264893
C	0.6177686	-3.0043914	1.9440037
C	1.3311531	-2.9281316	-2.3278285
C	-0.4349523	-1.1833560	-2.7567499
H	-2.4636121	3.4774666	1.7210570
H	-1.7674035	3.9859410	3.2613675
H	-2.5906566	2.4284606	3.1494138
H	1.1335115	4.2845115	2.8350866
H	1.9174674	3.4192697	1.4944085
H	0.5435336	4.4848872	1.1803191
C	0.7543989	3.1199514	-2.2606881
C	-1.5370513	2.8975026	-1.2385141
C	2.6811463	1.9470320	-1.4175842
C	2.5644910	0.3520464	0.5394760
C	2.0630868	-3.7700084	-1.4973865
H	2.4016134	-4.4218752	0.5187639
C	1.8487337	-2.5773280	2.7643788
C	0.1908219	-4.4288700	2.3489823
H	-0.1988461	-2.3209590	2.1966529
H	1.5127336	-2.9380757	-3.3986088
C	-1.3472328	-2.0380914	-3.6565240
C	0.4806631	-0.2908932	-3.6092711
H	-1.0673190	-0.5270398	-2.1509219
C	2.1141335	2.8375232	-2.3247985
H	0.3090827	3.7904547	-2.9900967
C	-2.2428591	2.3959263	-2.5117413
C	-1.7760263	4.4071847	-1.0569609
H	-1.9874525	2.3819188	-0.3842693
H	3.7366732	1.7065136	-1.4945026
C	3.3462563	1.0340824	1.6786597
C	3.4847091	-0.6246401	-0.2091735
H	1.7601274	-0.2344562	0.9930186
H	2.8150142	-4.4314147	-1.9174855
H	2.1158385	-1.5342183	2.5796281
H	1.6409632	-2.6908250	3.8334481
H	2.7149887	-3.2006593	2.5192194
H	-0.6527794	-4.7833064	1.7500297
H	1.0182410	-5.1325516	2.2109858
H	-0.0968715	-4.4504431	3.4054271
H	-1.9870907	-2.7029837	-3.0679344
H	-1.9859448	-1.3957744	-4.2722666
H	-0.7461944	-2.6616157	-4.3267586
H	1.1267628	0.3245478	-2.9793998
H	1.1134133	-0.8964451	-4.2665477
H	-0.1203113	0.3726724	-4.2395757
H	2.7292699	3.2937556	-3.0946869
H	-2.0984299	1.3203519	-2.6492025
H	-3.3173213	2.5993321	-2.4541207
H	-1.8488146	2.9004134	-3.3997809
H	-1.3025347	4.7839774	-0.1453885
H	-1.3678035	4.9695882	-1.9028262
H	-2.8494327	4.6156674	-0.9985552
H	2.6911457	1.5838255	2.3585418
H	3.8763060	0.2778151	2.2675597
H	4.0888083	1.7300142	1.2733092
H	2.9685586	-1.0887602	-1.0529479
H	4.3812130	-0.1192853	-0.5830699
H	3.8106067	-1.4162704	0.4718388

TS8^{+} : Si-F ring-closing via B-N rotation
84

Energy = -1948.802349406

B	0.0287160	0.2730360	-0.5645370
N	1.3574230	0.4043960	0.1189720
N	-1.1476190	0.1741930	0.2349020
C	0.0230100	0.1519300	-2.1350090
Si	1.8759520	1.4256800	1.3536680
C	2.4673500	-0.3644840	-0.4662290
Si	-1.3570110	-0.0529090	1.9859050
C	-2.4556890	0.1949230	-0.4381340
H	0.5404140	-0.7697940	-2.4352260
H	-0.9808860	0.1317020	-2.5650580
H	0.5798270	0.9663090	-2.6133110
C	3.3919120	0.9513710	2.2888430
C	0.8240790	2.7805060	2.0350530
F	0.1539290	0.0149880	2.6354440
C	2.5642430	-1.7416340	-0.2021690
C	3.4180760	0.3205470	-1.2490860
C	-2.0184970	-1.7121190	2.4828420
C	-2.3543260	1.2920790	2.7995220
C	-3.0551200	-0.9995660	-0.8942700
C	-3.0849090	1.4477850	-0.6293480
H	3.0629300	0.4177670	3.1911900
H	3.9521740	1.8363510	2.6064190
H	4.0385440	0.2913730	1.7063210
H	0.2732130	2.3591810	2.8813010
H	0.1211210	3.2011340	1.3120470
H	1.4635080	3.5805350	2.4239070
C	3.6903530	-2.4171480	-0.6888900
C	1.5129340	-2.4892760	0.6004940
C	4.5306740	-0.3950840	-1.7040520
C	3.2122920	1.7838410	-1.6360140
H	-3.0667430	-1.8382560	2.1999160
H	-1.9617690	-1.7563420	3.5785970
H	-1.4379930	-2.5482350	2.0815290
H	-1.9096290	2.2881240	2.7404270
H	-3.3580650	1.3426260	2.3635170
H	-2.4670230	1.0327350	3.8599130
C	-4.3103850	-0.9108870	-1.5132540
C	-2.3900030	-2.3674360	-0.8225990
C	-4.3330510	1.4801820	-1.2582480
C	-2.4091620	2.7503340	-0.2280280
C	4.6738520	-1.7503030	-1.4134150
H	3.7988940	-3.4800130	-0.4921970
C	1.9951080	-2.7434640	2.0406370
C	1.1020560	-3.8070430	-0.0764320
H	0.6225880	-1.8532920	0.6568710
H	5.2834600	0.1040630	-2.3053540
C	3.8671190	2.1425440	-2.9797430
C	3.6896480	2.7689740	-0.5614560
H	2.1327660	1.9358210	-1.7479910
C	-4.9498880	0.3095630	-1.6916560
H	-4.7846340	-1.8202840	-1.8724530
C	-1.9509070	-2.8345540	-2.2258300
C	-3.3141930	-3.4418370	-0.2181120
H	-1.4968220	-2.2789570	-0.1971790
H	-4.8274950	2.4343990	-1.4136960
C	-3.3864840	3.8145480	0.2927070
C	-1.5826050	3.3206590	-1.3968710
H	-1.7104540	2.5133530	0.5784760
H	5.5451280	-2.2915790	-1.7716510
H	2.2415940	-1.8082210	2.5509210
H	1.2148770	-3.2510210	2.6188890
H	2.8887870	-3.3775130	2.0374450
H	0.8060280	-3.6412240	-1.1166620
H	1.9216480	-4.5333820	-0.0687950
H	0.2568580	-4.2559870	0.4577620
H	3.5637100	1.4361880	-3.7587450
H	3.5593890	3.1483400	-3.2833680
H	4.9604240	2.1360170	-2.9143340
H	3.0164290	2.7925360	0.3363290
H	4.6896870	2.5417610	-0.1837470
H	3.6514170	3.8029660	-0.9194380
H	-5.9210860	0.3514730	-2.1771990
H	-1.2322180	-2.1512450	-2.6830760
H	-1.4922850	-3.8276810	-2.1624850
H	-2.8222280	-2.9076280	-2.8869360
H	-3.7721650	-3.1168070	0.7191180
